# Supplementary material for: Cohort study of cardiovascular safety of different COVID-19 vaccination doses among 46 million adults in England
Source: Nat Commun. 2024 Jul 31;15:6085. doi: 10.1038/s41467-024-49634-x (PMC11291640; doi:10.1038/s41467-024-49634-x)
Supplement: Supplementary file 1 — Supplementary Information [file 41467_2024_49634_MOESM1_ESM.pdf]

Cohort study of cardiovascular safety of different COVID-19 vaccination doses among 46 million adults in England

Contents – Supplementary Tables

Supplementary Table 1: Characterization of the population under study – dose 2 cohort 2
Supplementary Table 2: Characterization of the population under study – booster cohort 3
Supplementary Table 3: Definitions of the after-vaccination period and the corresponding period before or without vaccination in eligible individuals. The comparator group was chosen to include only those eligible for the specific vaccine brand and dose to assess the added risk of receiving it versus not. 4
Supplementary Table 4: Number of events, person-years and incidence rates for individual arterial and venous thrombotic events, and other cardiovascular events, for first and second vaccinations. 5
Supplementary Table 5: Number of events, person-years and incidence rates for individual arterial and venous thrombotic events, and other cardiovascular events, for booster vaccination with BNT-162b2. 6
Supplementary Table 6: Number of events, person-years and incidence rates for individual arterial and venous thrombotic events, and other cardiovascular events, for booster vaccination with mRNA-1273. 7
Supplementary Table 7: Number of events, person-years and incidence rates for individual arterial and venous thrombotic events, and other cardiovascular events, for booster vaccination with BNT-162b2/ mRNA-1273. 8
Supplementary Table 8: Hazard ratios and 95% CIs for individual arterial and venous thrombotic events, and other cardiovascular events, by time since first dose vaccination with ChAdOx1. 9
Supplementary Table 9: Hazard ratios and 95% CIs for individual arterial and venous thrombotic events, and other cardiovascular events, by time since first dose vaccination with BNT-162b2. 10
Supplementary Table 10: Hazard ratios and 95% CIs for individual arterial and venous thrombotic events, and other cardiovascular events, by time since first dose vaccination with mRNA-1273. 11
Supplementary Table 11: Hazard ratios and 95% CIs for individual arterial and venous thrombotic events, and other cardiovascular events, by time since second dose vaccination for those on a primary course of ChAdOx1 12
Supplementary Table 12: Hazard ratios and 95% CIs for individual arterial and venous thrombotic events, and other cardiovascular events, by time since second dose vaccination for those on a primary course of BNT-162b2. 13
Supplementary Table 13: Hazard ratios and 95% CIs for individual arterial and venous thrombotic events, and other cardiovascular events, by time since second dose vaccination for those on a primary course of mRNA-1273. 14
Supplementary Table 14: Hazard ratios and 95% CIs for individual arterial and venous thrombotic events, and other cardiovascular events, by time since booster vaccination with mRNA-1273 for those on a primary course of ChAdOx1. 15
Supplementary Table 15: Hazard ratios and 95% CIs for individual arterial and venous thrombotic events, and other cardiovascular events, by time since booster vaccination with BNT-162b2 for those on a primary course of ChAdOx1. 16
Supplementary Table 16: Hazard ratios and 95% CIs for individual arterial and venous thrombotic events, and other cardiovascular events, by time since booster vaccination with mRNA-1273 or BNT-162b2 for those on a primary course of ChAdOx1. 17
Supplementary Table 17: Hazard ratios and 95% CIs for individual arterial and venous thrombotic events, and other cardiovascular events, by time since booster vaccination with BNT-162b2 for those on a primary course of BNT-162b2. 18
Supplementary Table 18: Hazard ratios and 95% CIs for individual arterial and venous thrombotic events, and other cardiovascular events, by time since booster vaccination with mRNA-1273 for those on a primary course of BNT-162b2. 19
Supplementary Table 19: Hazard ratios and 95% CIs for individual arterial and venous thrombotic events, and other cardiovascular events, by time since booster vaccination with mRNA-1273 or BNT-162b2 for those on a primary course of BNT-162b2. 20
Supplementary Table 20: Hazard ratios and 95% CIs for individual arterial and venous thrombotic events, and other cardiovascular events, by time since booster vaccination with mRNA-1273 for those on a primary course of mRNA-1273 or BNT-162b2. 21
Supplementary Table 21: Hazard ratios and 95% CIs for individual arterial and venous thrombotic events, and other cardiovascular events, by time since booster vaccination with BNT-162b2 for those on a primary course of mRNA-1273 or BNT-162b2. 22
Supplementary Table 22: Hazard ratios and 95% CIs for individual arterial and venous thrombotic events, and other cardiovascular events, by time since booster vaccination with mRNA-1273 or BNT-162b2 for those on a primary course of mRNA-1273 or BNT-162b2. 23
Supplementary Table 23: Hazard ratios and 95% CIs for individual arterial and venous thrombotic events, and other cardiovascular events, by time since booster vaccination with mRNA-1273 for those on a primary course of ChAdOx1 or mRNA-1273 or BNT-162b2. 24
Supplementary Table 24: Hazard ratios and 95% CIs for individual arterial and venous thrombotic events, and other cardiovascular events, by time since booster vaccination with BNT-162b2 for those on a primary course of ChAdOx1 or mRNA-1273 or BNT-162b2. 25
Supplementary Table 25: Hazard ratios and 95% CIs for individual arterial and venous thrombotic events, and other cardiovascular events, by time since booster vaccination with mRNA-1273 or BNT-162b2 for those on a primary course of ChAdOx1 or mRNA-1273 or BNT-162b2. 26
Supplementary Table 26: Subgroup-specific maximally-adjusted hazard ratios and 95% CIs for composite arterial and composite venous events by time since first dose vaccination. 27
Supplementary Table 27: Subgroup-specific maximally-adjusted hazard ratios and 95% CIs for composite arterial and composite venous events by time since COVID-19 dose 1&2 vaccination. 28
Supplementary Table 28: Subgroup-specific maximally-adjusted hazard ratios and 95% CIs for composite arterial and composite venous events by time since booster vaccination with BNT-162b2 or mRNA-1273for those on a primary course of ChAdOx1. 29
Supplementary Table 29: Subgroup-specific maximally-adjusted hazard ratios and 95% CIs for composite arterial and composite venous events by time since booster with BNT-162b2 or mRNA-1273 for those on a primary course of BNT-162b2. 30
Supplementary Table 30: Associations of COVID-19 vaccination brand and dose with myocarditis and pericarditis until the CDC's Announcement on 17 May 2021. 31

Contents – Supplementary Figures

Supplementary Figure 1: Consort Flow Diagram for Population Inclusion/Exclusion Criteria* 32
Supplementary Figure 2: Subgroup-specific maximally-adjusted hazard ratios for a composite arterial event by time since dose 1 vaccination 33
Supplementary Figure 3: Subgroup-specific maximally-adjusted hazard ratios for a composite venous event by time since dose 1 vaccination 34
Supplementary Figure 4: Subgroup-specific maximally-adjusted hazard ratios for a composite arterial event by time since second dose vaccination 35
Supplementary Figure 5: Subgroup-specific maximally-adjusted hazard ratios for a composite venous event by time since second dose vaccination 36
Supplementary Figure 6: Subgroup-specific maximally-adjusted hazard ratios for a composite arterial event by time since booster vaccination 37
Supplementary Figure 7: Subgroup-specific maximally-adjusted hazard ratios for a composite venous event by time since booster vaccination 38
Supplementary Figure 8: Associations of COVID-19 vaccination brand and dose with myocarditis and pericarditis until the CDC's Announcement on 17 May 2021. 39

Supplementary Tables

Supplementary Table 1: Characterization of the population under study – dose 2 cohort[1]

		Those receiving dose 1 and eligible for dose 2 analysis	Vaccination status during dose 2 follow-up			
			Remains unvaccinated	ChAdOx1	BNT-162b2	mRNA-1273
**N (%)**	All	37249850	1396730 (3.75%)	18920225 (50.78%)	15961330 (42.85%)	971565 (2.61%)
**Sex (%)**	Male	17888920(48.02%)	719560(51.52%)	9228065(48.77%)	7412120(46.44%)	529175(54.47%)
	Female	19360930(51.98%)	677175(48.48%)	9692160(51.23%)	8549210(53.56%)	442385(45.53%)
**Age in years (%)**	18-29	5550865(14.90%)	480405(34.39%)	882970(4.67%)	3805665(23.84%)	381825(39.30%)
	30-39	5999655(16.11%)	361120(25.85%)	1423090(7.52%)	3854395(24.15%)	361050(37.16%)
	40-49	6070750(16.30%)	221850(15.88%)	4025920(21.28%)	1623400(10.17%)	199580(20.54%)
	50-59	6875270(18.46%)	136810(9.80%)	5233520(27.66%)	1480915(9.28%)	24025(2.47%)
	60-69	5530990(14.85%)	69350(4.97%)	3846470(20.33%)	1611345(10.10%)	3825(0.39%)
	70-79	4518335(12.13%)	48695(3.49%)	2660010(14.06%)	1808640(11.33%)	990(0.10%)
	80-89	2196915(5.90%)	48720(3.49%)	641655(3.39%)	1506310(9.44%)	230(0.02%)
	90+	507075(1.36%)	29785(2.13%)	206590(1.09%)	270660(1.70%)	40(0.00%)
**Ethnicity (%)**	Asian	2884895(7.74%)	162750(11.65%)	1283255(6.78%)	1371315(8.59%)	67575(6.96%)
	Black	1023140(2.75%)	95765(6.86%)	469890(2.48%)	431060(2.70%)	26425(2.72%)
	Mixed	468270(1.26%)	32965(2.36%)	189925(1.00%)	225715(1.41%)	19665(2.02%)
	Other	821435(2.21%)	45150(3.23%)	352840(1.86%)	390235(2.44%)	33210(3.42%)
	White	31194135(83.74%)	1026695(73.51%)	16202960(85.64%)	13174825(82.54%)	789655(81.28%)
	Unknown/Missing	857975(2.30%)	33405(2.39%)	421355(2.23%)	368180(2.31%)	35035(3.61%)
**Index of multiple deprivation (%) (lowest: most deprived; highest: last deprived).**	1-2	6449310(17.31%)	425815(30.49%)	3159100(16.70%)	2710950(16.98%)	153445(15.79%)
	3-4	7051080(18.93%)	322220(23.07%)	3482570(18.41%)	3057105(19.15%)	189185(19.47%)
	5-6	7396000(19.86%)	246955(17.68%)	3773515(19.94%)	3186060(19.96%)	189470(19.50%)
	7-8	7509665(20.16%)	196015(14.03%)	3931440(20.78%)	3195500(20.02%)	186710(19.22%)
	9-10	7498225(20.13%)	150785(10.80%)	4029670(21.30%)	3123915(19.57%)	193855(19.95%)
	Missing	1345575(3.61%)	54945(3.93%)	543935(2.87%)	687795(4.31%)	58900(6.06%)
**Smoking status (%)**	Current smoker	5538335(14.87%)	406905(29.13%)	2847490(15.05%)	2130020(13.34%)	153920(15.84%)
	Former smoker	9241400(24.81%)	241280(17.27%)	5200055(27.48%)	3647345(22.85%)	152720(15.72%)
	Never smoker	21262705(57.08%)	643395(46.06%)	10612005(56.09%)	9425100(59.05%)	582205(59.92%)
	Missing	1207410(3.24%)	105160(7.53%)	260670(1.38%)	758865(4.75%)	82715(8.51%)
**Medical history (%)**	AMI[2]	1093820(2.94%)	31955(2.29%)	559340(2.96%)	501770(3.14%)	755(0.08%)
	Diabetes	3692865(9.91%)	91845(6.58%)	1995145(10.55%)	1597505(10.01%)	8370(0.86%)
	Depression	8243045(22.13%)	376580(26.96%)	4503280(23.80%)	3208995(20.10%)	154190(15.87%)
	Obesity	4100435(11.01%)	131055(9.38%)	2293750(12.12%)	1633780(10.24%)	41850(4.31%)
	Cancer	6573585(17.65%)	199295(14.27%)	3353165(17.72%)	2895650(18.14%)	125475(12.91%)
	COPD[3]	1450370(3.89%)	48730(3.49%)	778620(4.12%)	621455(3.89%)	1565(0.16%)
	Liver disease	199445(0.54%)	10935(0.78%)	114865(0.61%)	73055(0.46%)	590(0.06%)
	Chronic kidney disease	2705645(7.26%)	88060(6.30%)	1327490(7.02%)	1285020(8.05%)	5075(0.52%)
	Dementia	476175(1.28%)	32680(2.34%)	256330(1.35%)	187000(1.17%)	165(0.02%)
	All stroke	783260(2.10%)	27395(1.96%)	415770(2.20%)	339285(2.13%)	810(0.08%)
	All VTE[4]	595620(1.60%)	26065(1.87%)	326365(1.72%)	241500(1.51%)	1690(0.17%)
	Thrombophilia	39750(0.11%)	1980(0.14%)	20235(0.11%)	17045(0.11%)	490(0.05%)
**Major surgery in the last year (%)**		3437715(9.23%)	168895(12.09%)	1640430(8.67%)	1571940(9.85%)	56450(5.81%)
**Number of unique diseases in the last year (%)**	0	29316520(78.70%)	1098255(78.63%)	14829750(78.38%)	12523280(78.46%)	865235(89.06%)
	1-5	7813000(20.97%)	290240(20.78%)	4024950(21.27%)	3391655(21.25%)	106155(10.93%)
	6+	120325(0.32%)	8230(0.59%)	65525(0.35%)	46395(0.29%)	175(0.02%)
**Region (%)**	Northwest	4707445(12.64%)	206320(14.77%)	2379905(12.58%)	2009445(12.59%)	111775(11.50%)
	Southeast	5674540(15.23%)	174660(12.50%)	2985210(15.78%)	2332460(14.61%)	182210(18.75%)
	London	5090755(13.67%)	237995(17.04%)	2323565(12.28%)	2366165(14.82%)	163030(16.78%)
	East of England	3439310(9.23%)	115845(8.29%)	1814770(9.59%)	1422585(8.91%)	86110(8.86%)
	Southwest	3167450(8.50%)	92920(6.65%)	1607885(8.50%)	1374400(8.61%)	92245(9.49%)
	Yorkshire and the Humber	3577500(9.60%)	140990(10.09%)	1876865(9.92%)	1453235(9.10%)	106410(10.95%)
	East Midlands	2679760(7.19%)	86955(6.23%)	1423665(7.52%)	1158690(7.26%)	10450(1.08%)
	West Midlands	3688035(9.90%)	154580(11.07%)	1913485(10.11%)	1553840(9.74%)	66130(6.81%)
	Northeast	1444085(3.88%)	58705(4.20%)	748500(3.96%)	602465(3.77%)	34415(3.54%)
	Missing	3780970(10.15%)	127760(9.15%)	1846375(9.76%)	1688050(10.58%)	118785(12.23%)
**Prior COVID-19 at index date**		2496910 (6.7%)	2496430(6.70%)	191945(13.74%)	1143330(6.04%)	1080500(6.77%)
**Medications taken in the last 3 months**	Antiplatelet	2341770(6.29%)	50545(3.62%)	1196220(6.32%)	1092935(6.85%)	2070(0.21%)
	Blood pressure lowering	8099995(21.75%)	137045(9.81%)	4493750(23.75%)	3454860(21.65%)	14340(1.48%)
	Lipid lowering	6475470(17.38%)	95400(6.83%)	3544760(18.74%)	2829820(17.73%)	5490(0.57%)
	Anticoagulant	1263200(3.39%)	35330(2.53%)	609895(3.22%)	617300(3.87%)	675(0.07%)
	COPD[3]	498145(1.34%)	17425(1.25%)	132500(0.70%)	320785(2.01%)	27435(2.82%)
	HRT[5]	513580(1.38%)	7435(0.53%)	348935(1.84%)	153750(0.96%)	3460(0.36%)
**Clinically vulnerable status**	Neither	27913300(74.94%)	1139475(81.58%)	13868865(73.30%)	11952700(74.89%)	952260(98.01%)
	Vulnerable	7372580(19.79%)	195030(13.96%)	3987980(21.08%)	3172470(19.88%)	17100(1.76%)
	Extremely vulnerable	1963965(5.27%)	62220(4.45%)	1063380(5.62%)	836160(5.24%)	2205(0.23%)

[1] All counts rounded to the nearest 5 and counts less than 10 displayed as '10'
[2] Acute myocardial infarction
[3] Combined oral contraceptives
[4] Venous Thromboembolic Event
[5] Hormone replacement therapy

Supplementary Table 2: Characterization of the population under study – booster cohort[1]

		Those receiving dose 2 and eligible for booster analysis	Vaccination status during booster follow-up			
			Remains unvaccinated	ChAdOx1	BNT-162b2	mRNA-1273
**N (%)**	All	35853120	7412330 (20.68%)	29885 (0.08%)	22085440 (61.59%)	6325465 (17.64%)
**Sex (%)**	Male	17169360(47.89%)	3736285(50.41%)	8745(29.26%)	10209690(46.23%)	3214640(50.82%)
	Female	18683760(52.11%)	3676045(49.59%)	21140(70.74%)	11875750(53.77%)	3110825(49.18%)
**Age in years (%)**	18-29	5045220(14.07%)	2339935(31.57%)	830(2.78%)	1897980(8.59%)	806475(12.75%)
	30-39	5641140(15.73%)	2014015(27.17%)	1480(4.95%)	2555375(11.57%)	1070270(16.92%)
	40-49	5844890(16.30%)	1356315(18.30%)	3165(10.59%)	3119305(14.12%)	1366105(21.60%)
	50-59	6741615(18.80%)	867965(11.71%)	5715(19.12%)	4210800(19.07%)	1657135(26.20%)
	60-69	5473745(15.27%)	394340(5.32%)	6000(20.08%)	4010445(18.16%)	1062960(16.80%)
	70-79	4471075(12.47%)	207090(2.79%)	6345(21.23%)	3971030(17.98%)	286610(4.53%)
	80-89	2134350(5.95%)	157045(2.12%)	4425(14.81%)	1911150(8.65%)	61730(0.98%)
	90+	501085(1.40%)	75625(1.02%)	1930(6.46%)	409355(1.85%)	14175(0.22%)
**Ethnicity (%)**	Asian	2722145(7.59%)	968180(13.06%)	1235(4.13%)	1387480(6.28%)	365250(5.77%)
	Black	927380(2.59%)	420175(5.67%)	1290(4.32%)	405225(1.83%)	100690(1.59%)
	Mixed	435305(1.21%)	155745(2.10%)	335(1.12%)	211630(0.96%)	67595(1.07%)
	Other	776285(2.17%)	256530(3.46%)	500(1.67%)	389265(1.76%)	129990(2.06%)
	White	30167440(84.14%)	5420120(73.12%)	26260(87.87%)	19230570(87.07%)	5490490(86.80%)
	Unknown/Missing	824565(2.30%)	191585(2.58%)	260(0.87%)	461270(2.09%)	171450(2.71%)
**Index of multiple deprivation (%) (lowest: most deprived; highest: last deprived).**	1-2	6052625(16.88%)	1902305(25.66%)	4990(16.70%)	3285255(14.88%)	860075(13.60%)
	3-4	6764755(18.87%)	1693300(22.84%)	6180(20.68%)	3942245(17.85%)	1123030(17.75%)
	5-6	7190695(20.06%)	1383720(18.67%)	6240(20.88%)	4504560(20.40%)	1296175(20.49%)
	7-8	7353800(20.51%)	1180665(15.93%)	6050(20.24%)	4780705(21.65%)	1386380(21.92%)
	9-10	7383720(20.59%)	977420(13.19%)	5710(19.11%)	4953800(22.43%)	1446790(22.87%)
	Missing	1107525(3.09%)	274920(3.71%)	715(2.39%)	618880(2.80%)	213010(3.37%)
**Smoking status (%)**	Current smoker	5111815(14.26%)	1610865(21.73%)	3655(12.23%)	2646775(11.98%)	850520(13.45%)
	Former smoker	9034100(25.20%)	1319225(17.80%)	9080(30.38%)	6207435(28.11%)	1498360(23.69%)
	Never smoker	20644655(57.58%)	3998635(53.95%)	16945(56.70%)	12830630(58.10%)	3798445(60.05%)
	Missing	1062550(2.96%)	483610(6.52%)	205(0.69%)	400595(1.81%)	178140(2.82%)
**Medical history (%)**	AMI[2]	1080380(3.01%)	118400(1.60%)	2100(7.03%)	861210(3.90%)	98670(1.56%)
	Diabetes	3707900(10.34%)	448405(6.05%)	6070(20.31%)	2807670(12.71%)	445755(7.05%)
	Depression	7938700(22.14%)	1578615(21.30%)	9540(31.92%)	4992600(22.61%)	1357945(21.47%)
	Obesity	4043865(11.28%)	631995(8.53%)	6085(20.36%)	2778290(12.58%)	627495(9.92%)
	Cancer	6774230(18.89%)	1138520(15.36%)	7300(24.43%)	4489645(20.33%)	1138765(18.00%)
	COPD[3]	1419320(3.96%)	171690(2.32%)	3495(11.69%)	1122390(5.08%)	121745(1.92%)
	Liver disease	193460(0.54%)	35640(0.48%)	400(1.34%)	134150(0.61%)	23270(0.37%)
	Chronic kidney disease	2675750(7.46%)	298285(4.02%)	6275(21.00%)	2164190(9.80%)	207000(3.27%)
	Dementia	465740(1.30%)	82695(1.12%)	1660(5.55%)	362440(1.64%)	18945(0.30%)
	All stroke	770990(2.15%)	91440(1.23%)	2050(6.86%)	608950(2.76%)	68550(1.08%)
	All VTE[4]	582505(1.62%)	79595(1.07%)	1400(4.68%)	436130(1.97%)	65380(1.03%)
	Thrombophilia	38600(0.11%)	6535(0.09%)	125(0.42%)	26350(0.12%)	5590(0.09%)
**Major surgery in the last year (%)**		3362750(9.38%)	631360(8.52%)	5150(17.23%)	2263465(10.25%)	462775(7.32%)
**Number of unique diseases in the last year (%)**	0	28248440 (78.8%)	6112540(82.46%)	18410(61.60%)	16858570(76.33%)	5258620(83.13%)
	1-5	7488270 (20.9%)	1278925(17.25%)	11095(37.13%)	5139995(23.27%)	1057700(16.72%)
	6+	117375 (0.33%)	20860(0.28%)	380(1.27%)	86880(0.39%)	9145(0.14%)
**Region (%)**	Northwest	4518635(12.60%)	1038860(14.02%)	3535(11.83%)	2816415(12.75%)	659825(10.43%)
	Southeast	5527815(15.42%)	926770(12.50%)	4835(16.18%)	3874390(17.54%)	721820(11.41%)
	London	4878390(13.61%)	1387180(18.71%)	7960(26.64%)	2681425(12.14%)	801825(12.68%)
	East of England	3348085(9.34%)	624965(8.43%)	2140(7.16%)	1948720(8.82%)	772260(12.21%)
	Southwest	3093925(8.63%)	493055(6.65%)	2920(9.77%)	1938650(8.78%)	659300(10.42%)
	Yorkshire and the Humber	3454215(9.63%)	722215(9.74%)	1765(5.91%)	2090985(9.47%)	639250(10.11%)
	East Midlands	2608510(7.28%)	491845(6.64%)	1655(5.54%)	1662850(7.53%)	452160(7.15%)
	West Midlands	3547765(9.90%)	771940(10.41%)	2110(7.06%)	2302170(10.42%)	471545(7.45%)
	Northeast	1391090(3.88%)	287400(3.88%)	880(2.94%)	700975(3.17%)	401835(6.35%)
	Missing	3484695(9.72%)	668115(9.01%)	2080(6.96%)	2068850(9.37%)	745650(11.79%)
**Prior COVID-19 at index date**		2792305 (7.79%)	928585(12.53%)	1840(6.16%)	1406635(6.37%)	455075(7.19%)
**Medications taken in the last 3 months**	Antiplatelet	2309730 (6.44%)	206265(2.78%)	4175(13.97%)	1891195(8.56%)	207940(3.29%)
	Blood pressure lowering	8040690 (22.4%)	671460(9.06%)	12155(40.67%)	6423250(29.08%)	933450(14.76%)
	Lipid lowering	6461885 (18.0%)	483320(6.52%)	8840(29.58%)	5280190(23.91%)	689315(10.90%)
	Anticoagulant	1261465 (3.52%)	114650(1.55%)	2735(9.15%)	1048740(4.75%)	95165(1.50%)
	COCP[3]	468320 (1.31%)	144360(1.95%)	155(0.52%)	231030(1.05%)	92775(1.47%)
	HRT[5]	536220 (1.50%)	48810(0.66%)	850(2.84%)	372085(1.68%)	114470(1.81%)
**Clinically vulnerable status**	Neither	26773975 (74.7%)	6295975(84.94%)	13825(46.26%)	15149610(68.60%)	5314410(84.02%)
	Vulnerable	7178100 (20.0%)	881140(11.89%)	9965(33.34%)	5408025(24.49%)	878420(13.89%)
	Extremely vulnerable	1902015 (5.30%)	235210(3.17%)	6090(20.38%)	1527810(6.92%)	132635(2.10%)

Supplementary Table 3: Definitions of the after-vaccination period and the corresponding period before or without vaccination in eligible individuals. Each comparison group included only individuals eligible to receive the vaccine brand and dose under consideration.

	Analysis	Post-vaccination	No vaccination
Dose 1	ChAdOx1	Post first dose of ChAdOx1	Unvaccinated or prior to the first dose of any vaccine
	BNT-162b2	Post first dose of BNT-162b2	
	mRNA-1273	Post first dose of mRNA-1273	
Dose 2	ChAdOx1	Post second dose of ChAdOx1, following a first dose of ChAdOx1	Between first dose of ChAdOx1 and second dose vaccination of any brand
	BNT-162b2	Post second dose of BNT-162b2, following a first dose of BNT-162b2	Between first dose of BNT-162b2 and second dose vaccination of any brand
	mRNA-1273	Post second dose of mRNA-1273, following a first dose of mRNA-1273	Between first dose of mRNA-1273 and second dose vaccination of any brand
Booster	Any booster dose (vs. ChAdOx1 primary course)	Post any mRNA booster vaccination (BNT-162b2 or mRNA-1273), following a primary course of ChAdOx1	Between second dose of ChAdOx1 and booster dose vaccination, following a primary course of ChAdOx1
	Any booster dose (vs. BNT-162b2 primary course)	Post any mRNA booster vaccination (BNT-162b2 or mRNA-1273), following a primary course of BNT-162b2	Between second dose of ChAdOx1 and booster dose vaccination, following a primary course of BNT-162b2
	Any booster dose (vs. mRNA primary course)	Post any mRNA booster vaccination (BNT-162b2 or mRNA-1273), following a primary course of either BNT-162b2 or mRNA-1273	Between second dose of ChAdOx1 and booster dose vaccination, following a primary course of either BNT-162b2 or mRNA-1273
	Any booster dose (vs. Any primary course)	Post any mRNA booster vaccination (BNT-162b2 or mRNA-1273), following a primary course of one of BNT-162b2 or mRNA-1273 or ChAdOx1	Between second dose of ChAdOx1 and booster dose vaccination, following a primary course of one of BNT-162b2 or mRNA-1273 or ChAdOx1
	BNT-162b2 booster dose (vs. ChAdOx1 primary course)	Post BNT-162b2 booster vaccination, following a primary course of ChAdOx1	Between second dose of ChAdOx1 and booster dose vaccination, following a primary course of ChAdOx1
	BNT-162b2 booster dose (vs. BNT-162b2 primary course)	Post BNT-162b2 booster vaccination, following a primary course of BNT-162b2	Between second dose of BNT-162b2 and booster dose vaccination, following a primary course of BNT-162b2
	BNT-162b2 booster dose (vs. mRNA primary course)	Post BNT-162b2 booster vaccination, following a primary course of either BNT-162b2 or mRNA-1273	Between second dose of either BNT-162b2 or mRNA-1273 and booster dose vaccination, following a primary course of either BNT-162b2 or mRNA-1273
	BNT-162b2 booster dose (vs. Any primary course)	Post BNT-162b2 booster vaccination, following a primary course of one of BNT-162b2 or mRNA-1273 or ChAdOx1	Between second dose of one of BNT-162b2 or mRNA-1273 or ChAdOx1 and booster dose vaccination, following a primary course of one of BNT-162b2 or mRNA-1273 or ChAdOx1
	mRNA-1273 booster dose (vs. ChAdOx1 primary course)	Post mRNA-1273 booster vaccination, following a primary course of ChAdOx1	Between second dose of ChAdOx1 and booster dose vaccination, following a primary course of ChAdOx1
	mRNA-1273 booster dose (vs. BNT-162b2 primary course)	Post mRNA-1273 booster vaccination, following a primary course of BNT-162b2	Between second dose of BNT-162b2 and booster dose vaccination, following a primary course of BNT-162b2
	mRNA-1273 booster dose (vs. mRNA primary course)	Post mRNA-1273 booster vaccination, following a primary course of either BNT-162b2 or mRNA-1273	Between second dose of either BNT-162b2 or mRNA-1273 and booster dose vaccination, following a primary course of either BNT-162b2 or mRNA-1273
	mRNA-1273 booster dose (vs Any primary course)	Post mRNA-1273 booster vaccination, following a primary course of one of BNT-162b2 or mRNA-1273 or ChAdOx1	Between second dose of one of BNT-162b2 or mRNA-1273 or ChAdOx1 and booster dose vaccination, following a primary course of one of BNT-162b2 or mRNA-1273 or ChAdOx1

Supplementary Table 4: Number of events, person-years and incidence rates for individual arterial and venous thrombotic events, and other cardiovascular events, for first and second vaccinations[6].

Vaccination	Event	Events/100K person-years		Incidence rate	
**Dose 1**		**No vaccination**	**After first dose**	**No vaccination**	**After first dose**
**ChAdOx1**	AMI[2]	37915/205.00	37675/ 95.87	184.95	392.97
	Ischaemic stroke	36720/205.00	35080/ 95.84	179.12	366.01
	Pulmonary embolism	11835/205.00	9695/ 95.81	57.73	101.19
	Lower limb DVT[7]	9075/205.00	7590/95.78	44.27	79.24
	ICVT[8]	370/205.00	290/96.28	1.80	3.01
	Portal vein thrombosis	185/205.00	175/95.79	0.90	1.83
	Any thrombocytopenia	1885/205.00	1760/95.98	9.20	18.34
	SAH and HS[9]	5235/205.00	4790/95.88	25.54	49.96
	Mesenteric thrombus	1515/205.00	1675/96.40	7.39	17.38
	Myocarditis	590/205.00	300/97.15	2.88	3.09
	Pericarditis	455/205.00	315/96.08	2.22	3.28
	Composite arterial	75655/205.00	73330/95.63	369.05	766.83
	Composite venous	21230/205.00	17525/96.09	103.56	182.39
**BNT-162b2**	AMI[2]	37915/205.00	28060/81.03	184.95	346.28
	Ischaemic stroke	36720/205.00	28535/81.06	179.12	352.04
	Pulmonary embolism	11835/205.00	6820/81.21	57.73	83.98
	Lower limb DVT[7]	9075/205.00	4895/81.37	44.27	60.16
	ICVT[8]	370/205.00	160/80.53	1.80	1.99
	Portal vein thrombosis	185/205.00	110/79.18	0.90	1.39
	Any thrombocytopenia	1885/205.00	1215/81.20	9.20	14.96
	SAH and HS[9]	5235/205.00	3575/81.60	25.54	43.81
	Mesenteric thrombus	1515/205.00	1310/81.12	7.39	16.15
	Myocarditis	590/205.00	315/81.44	2.88	3.87
	Pericarditis	455/205.00	270/78.92	2.22	3.42
	Composite arterial	75655/205.00	57050/80.99	369.05	704.38
	Composite venous	21230/205.00	11860/81.00	103.56	146.42
**mRNA-1273**	AMI[2]	37915/205.00	145/5.20	184.95	27.9
	Ischaemic stroke	36720/205.00	105/5.05	179.12	20.81
	Pulmonary embolism	11835/205.00	90/5.04	57.73	17.86
	Lower limb DVT[7]	9075/205.00	95/5.15	44.27	18.43
	ICVT[8]	370/205.00	10/5.09	1.80	1.97
	Portal vein thrombosis	185/205.00	10/4.33	0.90	2.31
	Any thrombocytopenia	1885/205.00	25/4.91	9.20	5.09
	SAH and HS[9]	5235/205.00	30/5.15	25.54	5.82
	Mesenteric thrombus	1515/205.00	10/5.03	7.39	1.99
	Myocarditis	590/205.00	50/4.91	2.88	10.19
	Pericarditis	455/205.00	20/5.26	2.22	3.8
	Composite arterial	75655/205.00	255/5.11	369.05	49.89
	Composite venous	21230/205.00	190/5.10	103.56	37.25
**Doses 1 and 2**		**After first dose**	**After second dose**	**After first dose**	**After second dose**
**ChAdOx1**	AMI[2]	19475/41.29	36210/93.48	471.69	387.35
	Ischaemic stroke	19440/41.25	33805/93.53	471.26	361.44
	Pulmonary embolism	4785/41.26	9000/93.63	115.98	96.12
	Lower limb DVT[7]	3730/41.28	6975/93.64	90.35	74.49
	ICVT[8]	190/41.29	170/93.60	4.6	1.82
	Portal vein thrombosis	85/41.48	145/93.49	2.05	1.55
	Any thrombocytopenia	1130/41.16	1400/93.77	27.45	14.93
	SAH and HS[9]	2385/41.31	4360/93.64	57.74	46.56
	Mesenteric thrombus	765/41.24	1555/93.64	18.55	16.61
	Myocarditis	145/41.23	285/93.75	3.52	3.04
	Pericarditis	160/41.70	280/93.36	3.84	3
	Composite arterial	39425/41.23	70485/93.36	956.17	755.01
	Composite venous	8685/41.27	16120/93.60	210.46	172.22
**BNT-162b2**	AMI[2]	13125/33.65	27295/72.57	390	376.12
	Ischaemic stroke	13575/33.62	27930/72.61	403.81	384.67
	Pulmonary embolism	3070/33.64	6545/72.75	91.26	89.96
	Lower limb DVT[7]	2115/33.62	4725/72.64	62.9	65.04
	ICVT[8]	70/33.60	145/73.05	2.08	1.98
	Portal vein thrombosis	50/33.67	105/72.40	1.48	1.45
	Any thrombocytopenia	675/33.70	1125/72.62	20.03	15.49
	SAH and HS[9]	1515/33.67	3440/72.72	45	47.31
	Mesenteric thrombus	565/33.70	1225/72.67	16.76	16.86
	Myocarditis	120/33.82	280/72.58	3.55	3.86
	Pericarditis	100/33.54	255/72.52	2.98	3.52
	Composite arterial	26975/33.63	55620/72.44	802.22	767.8
	Composite venous	5260/33.67	11400/72.69	156.22	156.83
**mRNA-1273**	AMI[2]	85/2.17	110/4.02	39.17	27.34
	Ischaemic stroke	70/2.16	90/4.06	32.39	22.14
	Pulmonary embolism	40/2.15	70/4.09	18.6	17.1
	Lower limb DVT[7]	50/2.17	60/4.03	23.04	14.88
	ICVT[8]	10/2.20	10/4.01	4.54	2.5
	Portal vein thrombosis	10/2.25	10/4.05	4.44	2.47
	Any thrombocytopenia	20/2.17	15/4.11	9.21	3.65
	SAH and HS[9]	10/2.29	25/3.99	4.37	6.27
	Mesenteric thrombus	10/2.22	10/4.19	4.51	2.39
	Myocarditis	15/2.13	45/4.07	7.04	11.05
	Pericarditis	10/2.19	15/4.07	4.57	3.68
	Composite arterial	160/2.17	200/4.06	73.66	49.24
	Composite venous	90/2.18	135/4.06	41.37	33.29

[6] Bold formatting identifies headers within the table, specifically at each vaccination dose, to clearly delineate comparison groups.

Supplementary Table 5: Number of events, person-years and incidence rates for individual arterial and venous thrombotic events, and other cardiovascular events, for booster vaccination with BNT-162b2.

**Vaccination**	**Event**	**Events/100K person-years**		**Incidence rate**	
		**Primary course only**	**After booster vaccination**	**Primary course only**	**After booster vaccination**
**Primary course**					
**Any**	AMI[2]	77890/182.46	19640/ 38.88	426.9	505.18
	Ischaemic stroke	78120/182.46	20050/ 38.92	428.16	515.15
	Pulmonary embolism	18580/182.66	4710/ 39.05	101.72	120.61
	Lower limb DVT[7]	13605/182.59	3105/ 39.05	74.51	79.51
	ICVT[8]	360/182.19	65/ 38.47	1.98	1.69
	Portal vein thrombosis	300/183.01	50/39.51	1.64	1.27
	Any thrombocytopenia	3340/182.60	680/ 39.10	18.29	17.39
	SAH and HS[9]	9110/182.67	2455/ 38.97	49.87	62.99
	Mesenteric thrombus	3300/182.62	845/ 39.49	18.07	21.4
	Myocarditis	675/182.54	120/ 39.15	3.7	3.06
	Pericarditis	615/182.84	110/ 39.39	3.36	2.79
	Composite arterial	157235/182.21	39735/ 38.72	862.93	1026.14
	Composite venous	32545/182.54	7855/ 38.97	178.29	201.54
**BNT-162b2/mRNA-1273**	AMI[2]	33945/80.40	10435/19.11	422.22	546
	Ischaemic stroke	35745/80.39	10985/19.11	444.63	574.85
	Pulmonary embolism	8010/80.43	2410/19.13	99.58	125.97
	Lower limb DVT[7]	5660/80.46	1550/19.23	70.34	80.59
	ICVT[8]	165/80.98	35/19.70	2.04	1.78
	Portal vein thrombosis	125/80.42	20/19.27	1.55	1.04
	Any thrombocytopenia	1535/80.42	340/19.18	19.09	17.72
	SAH and HS[9]	4115/80.43	1370/19.14	51.16	71.58
	Mesenteric thrombus	1505/80.57	485/19.22	18.68	25.23
	Myocarditis	345/80.19	60/19.17	4.3	3.13
	Pericarditis	295/80.22	55/19.26	3.68	2.86
	Composite arterial	70215/80.31	21425/19.04	874.28	1125.34
	Composite venous	13830/80.45	3975/19.15	171.91	207.62
**ChAdOx1**	AMI[2]	43945/102.06	9200/ 19.79	430.59	464.92
	Ischaemic stroke	42375/102.05	9065/ 19.78	415.23	458.31
	Pulmonary embolism	10570/102.23	2300/ 19.81	103.4	116.08
	Lower limb DVT[7]	7945/102.12	1555/ 19.82	77.8	78.44
	ICVT[8]	195/101.89	30/ 19.53	1.91	1.54
	Portal vein thrombosis	175/102.33	30/ 19.48	1.71	1.54
	Any thrombocytopenia	1800/102.15	340/ 19.81	17.62	17.16
	SAH and HS[9]	4990/102.14	1085/ 19.83	48.85	54.71
	Mesenteric thrombus	1795/102.18	360/ 19.82	17.57	18.16
	Myocarditis	330/102.26	60/ 20.15	3.23	2.98
	Pericarditis	320/102.42	55/ 19.94	3.12	2.76
	Composite arterial	87020/101.93	18310/ 19.76	853.73	926.59
	Composite venous	18715/102.13	3880/ 19.81	183.25	195.83
**BNT-162b2**	AMI[2]	33835/76.71	10430/18.87	441.1	552.7
	Ischaemic stroke	35650/76.69	10980/18.83	464.85	583.27
	Pulmonary embolism	7945/76.84	2405/18.92	103.39	127.08
	Lower limb DVT[7]	5600/76.76	1545/18.91	72.96	81.7
	ICVT[8]	160/76.41	35/18.65	2.09	1.88
	Portal vein thrombosis	120/76.40	20/18.89	1.57	1.06
	Any thrombocytopenia	1520/76.94	340/18.95	19.75	17.94
	SAH and HS[9]	4090/76.62	1365/18.89	53.38	72.27
	Mesenteric thrombus	1505/76.80	485/19.09	19.6	25.41
	Myocarditis	300/77.04	60/19.10	3.89	3.14
	Pericarditis	280/76.49	55/18.67	3.66	2.95
	Composite arterial	70005/76.60	21415/18.78	913.88	1140.44
	Composite venous	13700/76.75	3965/18.91	178.51	209.65

[7] Deep venous thrombosis
[8] Intracranial venous thrombosis
[9] Subarachnoid haemorrhage and haemorrhagic stroke

Supplementary Table 6: Number of events, person-years and incidence rates for individual arterial and venous thrombotic events, and other cardiovascular events, for booster vaccination with mRNA-1273.

Vaccination	Event	Events/100K person-years		Incidence rate	
		Primary course only	After booster vaccination	Primary course only	After booster vaccination
**Primary course**					
**Any**	AMI[2]	77890/182.41	1830/7.47	427.01	244.92
	Ischaemic stroke	78120/182.41	1345/7.46	428.26	180.32
	Pulmonary embolism	18580/182.60	430/7.47	101.75	57.55
	Lower limb DVT[7]	13605/182.66	320/7.51	74.48	42.59
	ICVT[8]	360/183.47	10/7.01	1.96	1.43
	Portal vein thrombosis	300/183.23	10/7.50	1.64	1.33
	Any thrombocytopenia	3340/182.65	50/7.41	18.29	6.75
	SAH and HS[9]	9110/182.86	150/7.42	49.82	20.21
	Mesenteric thrombus	3300/182.68	55/7.48	18.06	7.35
	Myocarditis	675/182.13	20/7.35	3.71	2.72
	Pericarditis	615/182.71	10/7.51	3.37	1.33
	Composite arterial	157235/182.22	3200/7.46	862.88	429.07
	Composite venous	32545/182.56	760/7.47	178.27	101.79
**BNT-162b2/mRNA-1273**	AMI[2]	33945/80.39	475/ 2.25	422.25	210.88
	Ischaemic stroke	35745/80.45	385/ 2.22	444.29	173.12
	Pulmonary embolism	8010/80.47	115/ 2.27	99.54	50.64
	Lower limb DVT[7]	5660/80.38	70/ 2.24	70.42	31.28
	ICVT[8]	165/80.45	10/ 2.23	2.05	4.48
	Portal vein thrombosis	125/79.95	10/ 2.26	1.56	4.43
	Any thrombocytopenia	1535/80.60	15/ 2.22	19.04	6.77
	SAH and HS[9]	4115/80.55	40/ 2.23	51.09	17.94
	Mesenteric thrombus	1505/80.45	20/ 2.24	18.71	8.92
	Myocarditis	345/80.61	10/ 2.20	4.28	4.54
	Pericarditis	295/80.23	10/ 2.28	3.68	4.39
	Composite arterial	70215/80.29	870/ 2.23	874.52	389.78
	Composite venous	13830/80.45	185/ 2.24	171.91	82.76
**ChAdOx1**	AMI[2]	43945/102.03	1355/5.23	430.71	258.97
	Ischaemic stroke	42375/102.03	960/5.24	415.31	183.38
	Pulmonary embolism	10570/102.13	310/5.21	103.49	59.49
	Lower limb DVT[7]	7945/102.13	250/5.22	77.79	47.86
	ICVT[8]	195/101.99	10/5.23	1.91	1.91
	Portal vein thrombosis	175/102.06	10/5.31	1.71	1.88
	Any thrombocytopenia	1800/102.04	35/5.16	17.64	6.78
	SAH and HS[9]	4990/102.24	110/5.19	48.81	21.18
	Mesenteric thrombus	1795/102.21	35/5.23	17.56	6.69
	Myocarditis	330/102.45	15/5.11	3.22	2.93
	Pericarditis	320/102.16	10/5.24	3.13	1.91
	Composite arterial	87020/101.95	2325/5.21	853.59	446.61
	Composite venous	18715/102.12	575/5.24	183.27	109.82
**BNT-162b2**	AMI[2]	33835/76.67	470/ 2.02	441.28	233.01
	Ischaemic stroke	35650/76.71	380/ 2.02	464.71	188.46
	Pulmonary embolism	7945/76.75	110/ 2.03	103.52	54.2
	Lower limb DVT[7]	5600/76.69	65/ 2.03	73.03	31.96
	ICVT[8]	160/77.25	10/ 1.95	2.07	5.13
	Portal vein thrombosis	120/77.51	10/ 1.91	1.55	5.23
	Any thrombocytopenia	1520/76.91	15/ 2.02	19.76	7.44
	SAH and HS[9]	4090/76.69	40/ 2.01	53.33	19.91
	Mesenteric thrombus	1505/76.69	20/ 2.05	19.62	9.77
	Myocarditis	300/76.49	10/ 1.96	3.92	5.1
	Pericarditis	280/76.54	10/ 1.91	3.66	5.23
	Composite arterial	70005/76.59	860/ 2.02	914	426.6
	Composite venous	13700/76.80	180/2.04	178.38	88.15

Supplementary Table 7: Number of events, person-years and incidence rates for individual arterial and venous thrombotic events, and other cardiovascular events, for booster vaccination with BNT-162b2/ mRNA-1273.

Vaccination	Event	Events/100K person-years		Incidence rate	
		Primary course only	After booster vaccination	Primary course only	After booster vaccination
**Primary course**					
**Any**	AMI[2]	77890/182.46	21470/ 46.36	426.89	463.11
	Ischaemic stroke	78120/182.43	21400/ 46.34	428.21	461.82
	Pulmonary embolism	18580/182.75	5135/ 46.51	101.67	101.41
	Lower limb DVT[7]	13605/182.56	3425/ 46.43	74.52	73.77
	ICVT[8]	360/182.76	75/ 46.59	1.97	1.61
	Portal vein thrombosis	300/183.14	60/ 46.34	1.64	1.29
	Any thrombocytopenia	3340/182.74	735/ 46.32	18.28	15.87
	SAH and HS[9]	9110/182.47	2605/ 46.48	49.93	56.04
	Mesenteric thrombus	3300/182.58	900/ 46.49	18.07	19.36
	Myocarditis	675/182.65	145/ 46.56	3.7	3.11
	Pericarditis	615/182.49	125/ 46.32	3.37	2.7
	Composite arterial	157235/182.21	42935/ 46.20	862.93	929.36
	Composite venous	32545/182.58	8615/ 46.37	178.25	185.79
**BNT-162b2/mRNA-1273**	AMI[2]	33945/80.41	10915/21.37	422.15	510.79
	Ischaemic stroke	35745/80.43	11370/21.33	444.45	532.94
	Pulmonary embolism	8010/80.55	2525/21.45	99.45	117.73
	Lower limb DVT[7]	5660/80.52	1615/21.38	70.29	75.56
	ICVT[8]	165/80.54	40/21.93	2.05	1.82
	Portal vein thrombosis	125/80.52	25/21.40	1.55	1.17
	Any thrombocytopenia	1535/80.59	355/21.31	19.05	16.66
	SAH and HS[9]	4115/80.56	1405/21.44	51.08	65.53
	Mesenteric thrombus	1505/80.60	505/21.54	18.67	23.44
	Myocarditis	345/80.88	70/21.78	4.27	3.21
	Pericarditis	295/80.13	60/21.37	3.68	2.81
	Composite arterial	70215/80.27	22300/21.25	874.73	1049.33
	Composite venous	13830/80.46	4165/21.39	171.88	194.72
**ChAdOx1**	AMI[2]	43945/102.06	10555/ 25.01	430.57	422.06
	Ischaemic stroke	42375/102.08	10030/ 25.00	415.12	401.21
	Pulmonary embolism	10570/102.15	2610/ 25.09	103.47	104.01
	Lower limb DVT[7]	7945/102.09	1805/ 25.07	77.82	71.99
	ICVT[8]	195/102.22	40/ 25.37	1.91	1.58
	Portal vein thrombosis	175/102.11	35/ 25.80	1.71	1.36
	Any thrombocytopenia	1800/102.22	380/ 25.10	17.61	15.14
	SAH and HS[9]	4990/102.17	1195/ 25.15	48.84	47.51
	Mesenteric thrombus	1795/102.08	395/ 25.10	17.58	15.74
	Myocarditis	330/102.35	75/ 25.11	3.22	2.99
	Pericarditis	320/102.15	65/ 25.06	3.13	2.59
	Composite arterial	87020/101.95	20635/ 24.94	853.54	827.32
	Composite venous	18715/102.08	4455/ 25.08	183.33	177.61
**BNT-162b2**	AMI[2]	33835/76.69	10900/20.86	441.19	522.5
	Ischaemic stroke	35650/76.64	11360/20.88	465.17	544.02
	Pulmonary embolism	7945/76.83	2515/20.93	103.4	120.15
	Lower limb DVT[7]	5600/76.68	1610/20.80	73.03	77.41
	ICVT[8]	160/76.07	40/20.92	2.1	1.91
	Portal vein thrombosis	120/76.52	25/21.39	1.57	1.17
	Any thrombocytopenia	1520/76.61	355/20.93	19.84	16.96
	SAH and HS[9]	4090/76.70	1405/20.90	53.32	67.24
	Mesenteric thrombus	1505/76.67	505/20.89	19.63	24.18
	Myocarditis	300/76.28	65/21.07	3.93	3.09
	Pericarditis	280/76.51	60/20.81	3.66	2.88
	Composite arterial	70005/76.59	22275/20.79	913.99	1071.28
	Composite venous	13700/76.73	4145/20.96	178.54	197.8

Supplementary Table 8: Hazard ratios and 95% CIs for individual arterial and venous thrombotic events, and other cardiovascular events, by time since first dose vaccination with ChAdOx1.

Outcome		Age and Sex adjusted HR (95% CI)						Fully Adjusted HR (95% CI)					
		Week 1	Week 2	Weeks 3-4	Weeks 5-12	Weeks 13-24	Weeks 25-26	Week 1	Week 2	Weeks 3-4	Weeks 5-12	Weeks 13-24	Weeks 25-26
**Arterial**	Composite arterial	0.90 (0.87,0.94)	1.06 (1.02,1.10)	1.09 (1.06,1.12)	1.17 (1.15,1.19)	1.26 (1.23,1.29)	1.32 (1.28,1.37)	0.84 (0.80,0.87)	0.97 (0.93,1.01)	0.97 (0.94,1.00)	1.00 (0.97,1.02)	0.99 (0.97,1.02)	0.98 (0.95,1.02)
	AMI[2]	0.94 (0.89,0.99)	1.03 (0.98,1.09)	1.11 (1.06,1.16)	1.22 (1.18,1.25)	1.31 (1.27,1.35)	1.41 (1.34,1.48)	0.87 (0.82,0.92)	0.95 (0.89,1.00)	0.99 (0.95,1.04)	1.03 (1.00,1.06)	1.02 (0.98,1.05)	1.02 (0.97,1.08)
	Ischaemic stroke	0.88 (0.83,0.93)	1.06 (1.00,1.12)	1.06 (1.01,1.10)	1.12 (1.09,1.16)	1.22 (1.18,1.27)	1.27 (1.20,1.34)	0.82 (0.77,0.87)	0.97 (0.92,1.03)	0.95 (0.91,0.99)	0.97 (0.93,1.00)	0.99 (0.95,1.03)	0.97 (0.91,1.03)
**Venous**	Composite venous	0.87 (0.80,0.94)	1.11 (1.03,1.20)	1.09 (1.03,1.15)	1.06 (1.02,1.10)	1.13 (1.09,1.18)	1.05 (0.98,1.13)	0.80 (0.74,0.87)	1.02 (0.94,1.09)	0.98 (0.93,1.04)	0.92 (0.88,0.96)	0.94 (0.90,0.98)	0.84 (0.78,0.90)
	Lower limb DVT[7]	0.89 (0.78,1.01)	1.09 (0.97,1.23)	1.14 (1.05,1.25)	1.10 (1.03,1.16)	1.11 (1.05,1.18)	1.00 (0.90,1.11)	0.82 (0.72,0.94)	1.00 (0.89,1.13)	1.04 (0.95,1.13)	0.96 (0.90,1.02)	0.93 (0.87,0.99)	0.81 (0.73,0.90)
	Pulmonary embolism	0.84 (0.76,0.93)	1.03 (0.93,1.13)	1.02 (0.94,1.10)	1.03 (0.98,1.09)	1.16 (1.09,1.22)	1.10 (1.00,1.21)	0.78 (0.70,0.86)	0.94 (0.85,1.03)	0.91 (0.85,0.99)	0.90 (0.85,0.95)	0.97 (0.91,1.02)	0.88 (0.80,0.97)
	ICVT[8]	1.39 (0.70,2.76)	6.08 (4.21,8.79)	4.09 (2.96,5.66)	1.37 (1.00,1.87)	1.49 (1.06,2.09)	1.72 (0.94,3.14)	1.37 (0.69,2.72)	5.92 (4.07,8.63)	3.91 (2.81,5.44)	1.25 (0.91,1.73)	1.34 (0.95,1.90)	1.60 (0.88,2.91)
	Mesenteric thrombus	0.64 (0.46,0.87)	1.09 (0.84,1.40)	1.07 (0.87,1.31)	1.15 (0.99,1.33)	1.38 (1.18,1.62)	1.54 (1.21,1.97)	0.56 (0.41,0.78)	0.94 (0.72,1.22)	0.91 (0.74,1.12)	0.90 (0.77,1.06)	0.98 (0.83,1.17)	0.99 (0.77,1.29)
	Portal vein thrombosis	0.86 (0.34,2.17)	1.16 (0.52,2.57)	1.06 (0.57,1.97)	1.21 (0.82,1.79)	1.51 (0.98,2.33)	1.96 (0.96,4.02)	0.61 (0.23,1.61)	0.80 (0.35,1.84)	0.70 (0.35,1.39)	0.78 (0.49,1.24)	0.76 (0.46,1.26)	0.88 (0.39,2.01)
**Other**	Any thrombocytopenia	1.45 (1.12,1.88)	2.53 (2.05,3.12)	2.00 (1.67,2.39)	1.79 (1.57,2.04)	2.16 (1.86,2.52)	1.98 (1.52,2.58)	1.22 (0.94,1.58)	2.07 (1.67,2.58)	1.60 (1.33,1.93)	1.24 (1.08,1.43)	1.28 (1.10,1.49)	1.04 (0.80,1.36)
	SAH and HS[9]	0.91 (0.78,1.06)	0.99 (0.86,1.16)	0.99 (0.89,1.11)	1.06 (0.98,1.15)	1.11 (1.02,1.20)	1.02 (0.89,1.18)	0.89 (0.76,1.04)	0.97 (0.83,1.13)	0.97 (0.86,1.09)	1.02 (0.94,1.10)	1.04 (0.95,1.13)	0.94 (0.81,1.08)
	Myocarditis	1.77 (1.06,2.96)	0.61 (0.27,1.37)	1.36 (0.90,2.05)	1.01 (0.77,1.34)	1.12 (0.87,1.45)	1.22 (0.77,1.95)	1.63 (0.98,2.72)	0.56 (0.25,1.26)	1.26 (0.84,1.90)	0.93 (0.70,1.22)	0.99 (0.77,1.28)	1.04 (0.66,1.65)
	Pericarditis	0.99 (0.50,1.96)	1.85 (1.11,3.09)	1.50 (0.98,2.30)	1.53 (1.18,1.98)	1.14 (0.88,1.49)	1.17 (0.71,1.94)	0.94 (0.48,1.86)	1.74 (1.04,2.91)	1.37 (0.89,2.11)	1.37 (1.05,1.78)	0.96 (0.73,1.26)	0.97 (0.60,1.59)

Supplementary Table 9: Hazard ratios and 95% CIs for individual arterial and venous thrombotic events, and other cardiovascular events, by time since first dose vaccination with BNT-162b2.

Outcome		Age and Sex adjusted HR (95% CI)						Fully Adjusted HR (95% CI)					
		Week 1	Week 2	Weeks 3-4	Weeks 5-12	Weeks 13-24	Weeks 25-26	Week 1	Week 2	Weeks 3-4	Weeks 5-12	Weeks 13-24	Weeks 25-26
**Arterial**	Composite arterial	0.80 (0.76,0.84)	0.90 (0.86,0.95)	0.96 (0.93,0.99)	1.04 (1.01,1.06)	1.13 (1.10,1.15)	1.13 (1.09,1.18)	0.73 (0.69,0.76)	0.80 (0.77,0.84)	0.83 (0.80,0.86)	0.85 (0.83,0.87)	0.90 (0.88,0.93)	0.91 (0.87,0.94)
	AMI[2]	0.88 (0.83,0.94)	1.00 (0.94,1.06)	1.01 (0.96,1.06)	1.12 (1.09,1.16)	1.22 (1.18,1.26)	1.25 (1.18,1.31)	0.76 (0.71,0.82)	0.85 (0.79,0.90)	0.83 (0.79,0.87)	0.88 (0.85,0.91)	0.93 (0.89,0.96)	0.95 (0.89,1.00)
	Ischaemic stroke	0.73 (0.68,0.77)	0.83 (0.78,0.88)	0.90 (0.86,0.95)	0.95 (0.92,0.98)	1.04 (1.01,1.08)	1.04 (0.98,1.10)	0.69 (0.65,0.74)	0.78 (0.74,0.84)	0.84 (0.80,0.88)	0.85 (0.82,0.88)	0.92 (0.88,0.96)	0.91 (0.86,0.97)
**Venous**	Composite venous	0.72 (0.65,0.79)	0.86 (0.78,0.94)	0.83 (0.78,0.89)	0.89 (0.86,0.93)	0.97 (0.93,1.01)	0.90 (0.84,0.98)	0.66 (0.60,0.73)	0.79 (0.72,0.87)	0.75 (0.70,0.81)	0.79 (0.76,0.82)	0.85 (0.81,0.88)	0.78 (0.72,0.84)
	Lower limb DVT[7]	0.75 (0.64,0.87)	0.91 (0.78,1.05)	0.81 (0.72,0.90)	0.89 (0.84,0.95)	0.92 (0.87,0.98)	0.88 (0.78,0.99)	0.71 (0.60,0.83)	0.85 (0.73,0.98)	0.75 (0.67,0.84)	0.80 (0.75,0.86)	0.82 (0.77,0.87)	0.78 (0.69,0.88)
	Pulmonary embolism	0.68 (0.60,0.78)	0.80 (0.71,0.90)	0.82 (0.75,0.90)	0.88 (0.83,0.93)	1.01 (0.95,1.06)	0.93 (0.83,1.03)	0.63 (0.55,0.72)	0.73 (0.65,0.83)	0.74 (0.68,0.81)	0.77 (0.73,0.82)	0.87 (0.82,0.93)	0.80 (0.72,0.89)
	ICVT[8]	0.17 (0.02,1.22)	1.91 (1.03,3.54)	1.21 (0.69,2.11)	0.90 (0.63,1.28)	1.29 (0.96,1.73)	1.15 (0.60,2.20)	0.16 (0.02,1.14)	1.68 (0.89,3.14)	1.07 (0.61,1.89)	0.79 (0.55,1.14)	1.12 (0.82,1.53)	1.02 (0.53,1.99)
	Mesenteric thrombus	0.62 (0.45,0.88)	0.79 (0.59,1.07)	0.82 (0.65,1.03)	1.00 (0.86,1.16)	1.22 (1.04,1.43)	1.11 (0.85,1.45)	0.55 (0.39,0.77)	0.69 (0.51,0.94)	0.67 (0.53,0.84)	0.75 (0.64,0.89)	0.87 (0.72,1.03)	0.76 (0.57,1.00)
	Portal vein thrombosis	0.89 (0.28,2.85)	0.52 (0.13,2.15)	0.82 (0.35,1.92)	1.41 (0.94,2.11)	1.55 (1.04,2.32)	0.61 (0.21,1.74)	0.60 (0.17,2.15)	0.39 (0.09,1.71)	0.59 (0.22,1.55)	1.05 (0.64,1.71)	1.17 (0.71,1.93)	0.45 (0.13,1.50)
**Other**	Any thrombocytopenia	1.61 (1.24,2.08)	0.96 (0.69,1.36)	1.47 (1.19,1.81)	1.43 (1.24,1.64)	1.66 (1.45,1.90)	1.67 (1.30,2.14)	1.25 (0.96,1.64)	0.74 (0.52,1.04)	1.09 (0.87,1.35)	0.97 (0.83,1.13)	1.08 (0.92,1.25)	1.03 (0.79,1.34)
	SAH and HS[9]	0.64 (0.53,0.78)	0.66 (0.54,0.80)	0.78 (0.68,0.89)	0.88 (0.81,0.96)	1.02 (0.94,1.11)	1.02 (0.87,1.18)	0.64 (0.53,0.78)	0.65 (0.54,0.80)	0.77 (0.68,0.88)	0.88 (0.80,0.96)	1.03 (0.94,1.13)	1.04 (0.89,1.21)
	Myocarditis	2.21 (1.38,3.55)	1.52 (0.86,2.66)	1.15 (0.72,1.81)	1.47 (1.16,1.85)	1.26 (1.00,1.58)	1.96 (1.33,2.89)	2.05 (1.28,3.29)	1.41 (0.81,2.48)	1.07 (0.67,1.70)	1.34 (1.05,1.70)	1.12 (0.89,1.41)	1.66 (1.12,2.47)
	Pericarditis	0.98 (0.46,2.07)	0.85 (0.38,1.93)	1.07 (0.63,1.82)	1.87 (1.46,2.40)	1.75 (1.38,2.21)	0.89 (0.44,1.78)	0.87 (0.41,1.86)	0.77 (0.34,1.74)	0.95 (0.56,1.63)	1.61 (1.24,2.08)	1.50 (1.17,1.92)	0.74 (0.37,1.50)

Supplementary Table 10: Hazard ratios and 95% CIs for individual arterial and venous thrombotic events, and other cardiovascular events, by time since first dose vaccination with mRNA-1273.

Outcome		Age and Sex adjusted HR (95% CI)						Fully Adjusted HR (95% CI)					
		Week 1	Week 2	Weeks 3-4	Weeks 5-12	Weeks 13-24	Weeks 25-26	Week 1	Week 2	Weeks 3-4	Weeks 5-12	Weeks 13-24	Weeks 25-26
**Arterial**	Composite arterial	0.74 (0.41, 1.34)	0.60 (0.31, 1.16)	1.09 (0.77, 1.55)	0.76 (0.61, 0.94)	0.66 (0.54, 0.80)	0.89 (0.58, 1.37)	0.74 (0.41, 1.34)	0.60 (0.31, 1.16)	1.08 (0.76, 1.53)	0.75 (0.60, 0.93)	0.64 (0.52, 0.78)	0.86 (0.56, 1.32)
	AMI[2]	0.69 (0.31, 1.53)	0.69 (0.31, 1.53)	1.16 (0.74, 1.80)	0.67 (0.50, 0.91)	0.69 (0.54, 0.90)	0.63 (0.33, 1.22)	0.71 (0.32, 1.58)	0.69 (0.31, 1.55)	1.17 (0.75, 1.82)	0.68 (0.50, 0.92)	0.68 (0.53, 0.89)	0.60 (0.31, 1.16)
	Ischaemic stroke	0.96 (0.43, 2.14)	0.48 (0.15, 1.49)	0.89 (0.49, 1.62)	0.82 (0.59, 1.13)	0.56 (0.40, 0.79)	1.28 (0.72, 2.26)	0.94 (0.42, 2.10)	0.48 (0.15, 1.49)	0.89 (0.49, 1.62)	0.80 (0.57, 1.12)	0.53 (0.38, 0.76)	1.18 (0.66, 2.09)
**Venous**	Composite venous	0.91 (0.47, 1.74)	0.90 (0.47, 1.73)	0.69 (0.41, 1.18)	0.69 (0.53, 0.90)	0.63 (0.50, 0.79)	1.05 (0.68, 1.64)	0.90 (0.47, 1.73)	0.90 (0.47, 1.73)	0.69 (0.41, 1.17)	0.68 (0.52, 0.89)	0.62 (0.49, 0.78)	1.03 (0.66, 1.60)
	Lower limb DVT[7]	0.17 (0.02, 1.24)	0.86 (0.36, 2.08)	0.78 (0.40, 1.50)	0.67 (0.47, 0.96)	0.53 (0.38, 0.74)	1.18 (0.67, 2.09)	0.17 (0.02, 1.23)	0.87 (0.36, 2.11)	0.77 (0.40, 1.48)	0.66 (0.47, 0.95)	0.52 (0.37, 0.73)	1.15 (0.65, 2.04)
	Pulmonary embolism	1.69 (0.80, 3.57)	0.96 (0.36, 2.57)	0.59 (0.25, 1.43)	0.73 (0.49, 1.07)	0.73 (0.53, 1.00)	0.89 (0.44, 1.80)	1.62 (0.77, 3.42)	0.91 (0.34, 2.43)	0.57 (0.23, 1.36)	0.69 (0.47, 1.02)	0.70 (0.51, 0.96)	0.84 (0.42, 1.70)
	ICVT[8]*			1.40 (0.33, 5.99)			0.59 (0.21, 1.67)			1.50 (0.36, 6.23)			0.57 (0.20, 1.63)
	Mesenteric thrombus*			1.96 (0.47, 8.13)			0.76 (0.28, 2.08)			1.72 (0.42, 7.09)			0.67 (0.24, 1.82)
	Portal vein thrombosis												
**Other**	Any thrombocytopenia			2.17 (1.01, 4.66)			1.22 (0.75, 1.98)			1.99 (0.92, 4.32)			1.12 (0.69, 1.83)
	SAH and HS[9]			0.80 (0.33, 1.95)			0.80 (0.53, 1.20)			0.83 (0.34, 2.03)			0.82 (0.54, 1.25)
	Myocarditis	5.57 (1.70,18.26)	1.63 (0.22,11.86)	0.87 (0.12, 6.42)	5.64 (3.61, 8.81)	1.95 (1.12, 3.40)	2.09 (0.49, 8.88)	4.64 (1.40,15.31)	1.52 (0.21,10.99)	0.87 (0.12, 6.45)	5.35 (3.37, 8.48)	1.78 (1.00, 3.16)	2.05 (0.49, 8.66)
	Pericarditis	1.75 (0.24,12.93)	1.91 (0.26,13.99)	2.07 (0.49, 8.79)	1.97 (0.87, 4.44)	1.83 (0.89, 3.79)	2.34 (0.56, 9.84)	2.01 (0.26,15.31)	1.85 (0.25,13.87)	2.11 (0.49, 9.06)	1.94 (0.88, 4.29)	1.95 (0.95, 4.01)	2.16 (0.52, 9.01)

*Time periods collapsed into weeks 0-4 and weeks 5-26.

Models could not be run for portal vein thrombosis.

Supplementary Table 11: Hazard ratios and 95% CIs for individual arterial and venous thrombotic events, and other cardiovascular events, by time since second dose vaccination for those on a primary course of ChAdOx1

**Outcome**		**Age and Sex adjusted HR (95% CI)**						**Fully Adjusted HR (95% CI)**					
		Week 1	Week 2	Weeks 3-4	Weeks 5-12	Weeks 13-24	Weeks 25-26	Week 1	Week 2	Weeks 3-4	Weeks 5-12	Weeks 13-24	Weeks 25-26
**Arterial**	Composite arterial	0.73 (0.70,0.76)	0.84 (0.81,0.88)	0.81 (0.78,0.84)	0.78 (0.76,0.81)	0.78 (0.75,0.81)	0.81 (0.77,0.85)	0.72 (0.69,0.75)	0.83 (0.80,0.87)	0.79 (0.77,0.82)	0.76 (0.74,0.78)	0.73 (0.70,0.76)	0.73 (0.70,0.77)
	AMI[2]	0.76 (0.71,0.80)	0.87 (0.82,0.92)	0.86 (0.82,0.89)	0.83 (0.80,0.87)	0.84 (0.80,0.89)	0.88 (0.82,0.95)	0.73 (0.69,0.78)	0.84 (0.79,0.89)	0.82 (0.78,0.85)	0.77 (0.74,0.80)	0.73 (0.70,0.77)	0.73 (0.68,0.79)
	Ischaemic stroke	0.71 (0.67,0.75)	0.82 (0.77,0.87)	0.77 (0.74,0.81)	0.75 (0.72,0.78)	0.75 (0.70,0.79)	0.76 (0.70,0.82)	0.71 (0.67,0.76)	0.83 (0.78,0.88)	0.78 (0.75,0.82)	0.76 (0.73,0.80)	0.76 (0.72,0.80)	0.77 (0.71,0.83)
**Venous**	Composite venous	0.74 (0.67,0.80)	0.86 (0.79,0.94)	0.82 (0.77,0.88)	0.75 (0.70,0.80)	0.75 (0.69,0.81)	0.80 (0.72,0.89)	0.72 (0.66,0.79)	0.84 (0.78,0.92)	0.80 (0.75,0.85)	0.71 (0.67,0.75)	0.68 (0.63,0.73)	0.69 (0.63,0.77)
	Lower limb DVT[7]	0.72 (0.64,0.83)	0.81 (0.72,0.92)	0.80 (0.72,0.88)	0.71 (0.64,0.77)	0.68 (0.61,0.77)	0.72 (0.61,0.84)	0.71 (0.63,0.81)	0.80 (0.70,0.90)	0.77 (0.70,0.86)	0.67 (0.61,0.73)	0.62 (0.55,0.69)	0.62 (0.54,0.73)
	Pulmonary embolism	0.76 (0.67,0.85)	0.90 (0.81,1.01)	0.83 (0.76,0.91)	0.78 (0.72,0.85)	0.79 (0.71,0.89)	0.85 (0.73,0.98)	0.75 (0.66,0.84)	0.88 (0.79,0.99)	0.81 (0.74,0.89)	0.74 (0.68,0.80)	0.71 (0.64,0.79)	0.73 (0.64,0.84)
	ICVT[8]	0.30 (0.11,0.84)	0.96 (0.51,1.80)	0.93 (0.56,1.56)	0.67 (0.40,1.12)	0.74 (0.37,1.48)	0.90 (0.33,2.44)	0.29 (0.11,0.82)	0.94 (0.50,1.76)	0.88 (0.53,1.48)	0.61 (0.36,1.04)	0.63 (0.31,1.28)	0.73 (0.27,1.96)
	Mesenteric thrombus	0.64 (0.47,0.87)	1.05 (0.81,1.36)	0.77 (0.61,0.97)	0.77 (0.62,0.96)	0.76 (0.58,1.01)	0.76 (0.52,1.09)	0.62 (0.45,0.84)	1.01 (0.78,1.31)	0.73 (0.58,0.92)	0.70 (0.57,0.87)	0.64 (0.49,0.83)	0.60 (0.42,0.85)
	Portal vein thrombosis	0.66 (0.25,1.71)	0.39 (0.11,1.37)	0.92 (0.46,1.85)	1.26 (0.67,2.38)	1.44 (0.55,3.75)	2.50 (0.70,8.87)	0.49 (0.18,1.34)	0.31 (0.09,1.09)	0.67 (0.32,1.40)	0.72 (0.36,1.43)	0.52 (0.19,1.42)	0.78 (0.22,2.74)
**Other**	Any thrombocytopenia	0.89 (0.69,1.14)	0.76 (0.57,1.01)	0.95 (0.76,1.18)	1.01 (0.81,1.25)	1.43 (1.05,1.96)	1.83 (1.18,2.82)	0.73 (0.57,0.94)	0.60 (0.46,0.79)	0.71 (0.57,0.87)	0.62 (0.52,0.75)	0.66 (0.51,0.86)	0.67 (0.46,0.96)
	SAH and HS[9]	0.61 (0.51,0.72)	0.76 (0.64,0.89)	0.75 (0.66,0.85)	0.68 (0.60,0.76)	0.60 (0.52,0.69)	0.59 (0.49,0.72)	0.62 (0.52,0.74)	0.77 (0.66,0.91)	0.78 (0.68,0.88)	0.71 (0.63,0.80)	0.64 (0.56,0.74)	0.64 (0.53,0.77)
	Myocarditis	0.49 (0.23,1.07)	0.75 (0.40,1.40)	0.64 (0.38,1.07)	0.75 (0.51,1.12)	0.68 (0.40,1.13)	0.73 (0.36,1.48)	0.48 (0.22,1.04)	0.75 (0.40,1.40)	0.63 (0.38,1.05)	0.72 (0.50,1.05)	0.64 (0.40,1.02)	0.67 (0.35,1.29)
	Pericarditis	1.34 (0.84,2.16)	0.80 (0.44,1.45)	0.69 (0.43,1.10)	0.50 (0.33,0.75)	0.49 (0.29,0.81)	0.59 (0.28,1.21)	1.21 (0.76,1.95)	0.68 (0.38,1.24)	0.58 (0.36,0.91)	0.39 (0.26,0.57)	0.32 (0.19,0.51)	0.33 (0.17,0.65)

Supplementary Table 12: Hazard ratios and 95% CIs for individual arterial and venous thrombotic events, and other cardiovascular events, by time since second dose vaccination for those on a primary course of BNT-162b2.

Outcome		Age and Sex adjusted HR (95% CI)						Fully Adjusted HR (95% CI)					
		Week 1	Week 2	Weeks 3-4	Weeks 5-12	Weeks 13-24	Weeks 25-26	Week 1	Week 2	Weeks 3-4	Weeks 5-12	Weeks 13-24	Weeks 25-26
**Arterial**	Composite arterial	0.71 (0.68,0.75)	0.80 (0.77,0.84)	0.81 (0.78,0.84)	0.80 (0.78,0.83)	0.80 (0.77,0.83)	0.80 (0.76,0.84)	0.73 (0.69,0.76)	0.82 (0.78,0.85)	0.82 (0.79,0.85)	0.81 (0.78,0.83)	0.80 (0.77,0.83)	0.80 (0.76,0.85)
	AMI[2]	0.70 (0.65,0.75)	0.85 (0.79,0.90)	0.83 (0.79,0.88)	0.82 (0.79,0.86)	0.85 (0.81,0.89)	0.86 (0.80,0.93)	0.71 (0.66,0.76)	0.85 (0.80,0.91)	0.84 (0.79,0.88)	0.81 (0.78,0.85)	0.82 (0.78,0.87)	0.83 (0.77,0.90)
	Ischaemic stroke	0.73 (0.68,0.78)	0.76 (0.71,0.81)	0.78 (0.74,0.82)	0.79 (0.75,0.82)	0.76 (0.72,0.80)	0.75 (0.70,0.81)	0.74 (0.69,0.79)	0.77 (0.72,0.82)	0.79 (0.75,0.83)	0.80 (0.76,0.84)	0.77 (0.72,0.81)	0.76 (0.71,0.83)
**Venous**	Composite venous	0.73 (0.66,0.81)	0.82 (0.74,0.91)	0.77 (0.71,0.84)	0.79 (0.74,0.84)	0.83 (0.77,0.89)	0.82 (0.74,0.91)	0.73 (0.66,0.81)	0.82 (0.74,0.90)	0.77 (0.71,0.83)	0.76 (0.72,0.81)	0.77 (0.72,0.83)	0.74 (0.67,0.83)
	Lower limb DVT[7]	0.74 (0.63,0.87)	0.84 (0.72,0.98)	0.83 (0.73,0.94)	0.81 (0.74,0.89)	0.82 (0.73,0.91)	0.77 (0.65,0.91)	0.75 (0.63,0.88)	0.85 (0.73,0.99)	0.84 (0.74,0.95)	0.82 (0.74,0.90)	0.81 (0.73,0.91)	0.77 (0.65,0.91)
	Pulmonary embolism	0.72 (0.63,0.83)	0.79 (0.69,0.91)	0.74 (0.66,0.82)	0.76 (0.70,0.83)	0.83 (0.75,0.92)	0.84 (0.73,0.97)	0.73 (0.63,0.84)	0.80 (0.69,0.91)	0.74 (0.66,0.82)	0.74 (0.68,0.81)	0.79 (0.71,0.87)	0.78 (0.68,0.90)
	ICVT[8]	0.47 (0.17,1.30)	1.16 (0.55,2.44)	0.71 (0.36,1.37)	0.74 (0.47,1.16)	0.88 (0.53,1.47)	0.34 (0.11,1.06)	0.51 (0.18,1.43)	1.28 (0.61,2.70)	0.76 (0.38,1.50)	0.77 (0.49,1.21)	0.87 (0.52,1.45)	0.32 (0.10,0.99)
	Mesenteric thrombus	0.55 (0.38,0.80)	0.71 (0.52,0.99)	0.86 (0.67,1.11)	0.66 (0.53,0.83)	0.71 (0.54,0.92)	0.70 (0.49,1.02)	0.54 (0.38,0.79)	0.70 (0.51,0.97)	0.84 (0.65,1.08)	0.63 (0.50,0.79)	0.64 (0.49,0.83)	0.63 (0.43,0.91)
	Portal vein thrombosis	0.68 (0.23,2.01)	0.33 (0.07,1.49)	0.64 (0.23,1.76)	0.93 (0.48,1.81)	0.89 (0.41,1.91)	0.70 (0.22,2.22)	0.57 (0.17,1.97)	0.28 (0.06,1.28)	0.61 (0.21,1.81)	0.79 (0.36,1.72)	0.60 (0.22,1.70)	0.48 (0.11,2.06)
**Other**	Any thrombocytopenia	0.84 (0.62,1.13)	0.77 (0.56,1.05)	1.00 (0.80,1.25)	0.89 (0.74,1.06)	1.18 (0.95,1.47)	1.39 (0.99,1.96)	0.78 (0.58,1.06)	0.72 (0.52,0.98)	0.92 (0.73,1.16)	0.74 (0.62,0.89)	0.87 (0.70,1.09)	0.94 (0.67,1.33)
	SAH and HS[9]	0.69 (0.56,0.85)	0.83 (0.68,1.00)	0.95 (0.82,1.10)	0.86 (0.76,0.98)	0.81 (0.69,0.94)	0.73 (0.59,0.91)	0.70 (0.57,0.86)	0.85 (0.70,1.02)	0.97 (0.84,1.13)	0.89 (0.78,1.01)	0.84 (0.73,0.98)	0.77 (0.62,0.96)
	Myocarditis	3.21 (2.10,4.92)	1.60 (0.93,2.77)	0.97 (0.58,1.61)	1.07 (0.77,1.49)	0.92 (0.65,1.31)	1.32 (0.71,2.43)	3.14 (2.04,4.85)	1.63 (0.94,2.82)	0.98 (0.59,1.63)	1.11 (0.79,1.56)	0.95 (0.66,1.38)	1.42 (0.76,2.65)
	Pericarditis	0.91 (0.43,1.90)	1.44 (0.80,2.61)	2.37 (1.59,3.54)	1.38 (0.98,1.93)	1.18 (0.79,1.77)	1.65 (0.84,3.23)	0.86 (0.41,1.81)	1.45 (0.80,2.63)	2.42 (1.62,3.62)	1.34 (0.96,1.88)	1.16 (0.77,1.72)	1.61 (0.83,3.13)

Supplementary Table 13: Hazard ratios and 95% CIs for individual arterial and venous thrombotic events, and other cardiovascular events, by time since second dose vaccination for those on a primary course of mRNA-1273.

Outcome		Age and Sex adjusted HR (95% CI)						Fully Adjusted HR (95% CI)					
		Week 1	Week 2	Weeks 3-4	Weeks 5-12	Weeks 13-24	Weeks 25-26	Week 1	Week 2	Weeks 3-4	Weeks 5-12	Weeks 13-24	Weeks 25-26
**Arterial**	Composite arterial	0.56 (0.26, 1.21)	1.38 (0.78, 2.42)	0.47 (0.24, 0.91)	0.64 (0.43, 0.94)	0.74 (0.47, 1.17)	0.35 (0.14, 0.92)	0.78 (0.36, 1.67)	1.60 (0.90, 2.85)	0.62 (0.32, 1.23)	0.81 (0.52, 1.27)	1.09 (0.64, 1.83)	0.59 (0.22, 1.62)
	AMI[2]	0.98 (0.38, 2.54)	2.08 (1.02, 4.25)	0.45 (0.17, 1.19)	0.69 (0.37, 1.29)	0.52 (0.27, 0.98)	0.36 (0.11, 1.16)	1.05 (0.40, 2.75)	2.39 (1.11, 5.17)	0.59 (0.23, 1.51)	0.86 (0.44, 1.69)	0.75 (0.34, 1.66)	0.53 (0.14, 2.07)
	Ischaemic stroke	0.22 (0.05, 1.01)	0.76 (0.25, 2.34)	0.50 (0.18, 1.39)	0.66 (0.35, 1.24)	1.01 (0.49, 2.11)	0.12 (0.01, 0.97)	0.39 (0.08, 1.89)	1.13 (0.38, 3.35)	0.56 (0.19, 1.67)	0.69 (0.35, 1.34)	1.11 (0.55, 2.22)	0.13 (0.02, 0.83)
**Venous**	Composite venous	0.65 (0.28, 1.52)	0.21 (0.05, 0.88)	0.81 (0.44, 1.49)	0.61 (0.39, 0.95)	0.57 (0.36, 0.90)	0.25 (0.06, 1.05)	0.74 (0.31, 1.77)	0.24 (0.06, 0.99)	0.85 (0.45, 1.59)	0.72 (0.45, 1.16)	0.81 (0.46, 1.45)	0.41 (0.08, 2.00)
	Lower limb DVT[7]	0.66 (0.23, 1.89)	0.16 (0.02, 1.23)	0.46 (0.18, 1.20)	0.38 (0.20, 0.71)	0.40 (0.20, 0.77)	0.20 (0.02, 1.67)	0.95 (0.32, 2.78)	0.20 (0.03, 1.56)	0.56 (0.20, 1.53)	0.49 (0.24, 0.99)	0.52 (0.25, 1.08)	0.30 (0.04, 2.38)
	Pulmonary embolism	0.64 (0.15, 2.82)	0.33 (0.04, 2.52)	1.48 (0.64, 3.43)	0.94 (0.47, 1.88)	0.80 (0.39, 1.63)	0.32 (0.04, 2.72)	0.65 (0.14, 3.02)	0.27 (0.03, 2.45)	1.54 (0.68, 3.51)	1.01 (0.50, 2.07)	0.96 (0.46, 1.97)	0.36 (0.04, 3.01)
	ICVT[8]												
	Mesenteric thrombus												
	Portal vein thrombosis												
**Other**	Any thrombocytopenia*			1.17 (0.31, 4.37)			0.92 (0.15, 5.45)			1.08 (0.24, 4.84)			0.91 (0.11, 7.52)
	SAH and HS[9]*			0.36 (0.06, 2.28)			1.02 (0.31, 3.33)			0.25 (0.04, 1.63)			0.70 (0.24, 2.10)
	Myocarditis*			7.62 (2.82,20.58)			0.47 (0.14, 1.50)			10.8 (3.79, 30.83)			0.65 (0.20, 2.11)
	Pericarditis*			1.93 (0.58, 6.44)			1.06 (0.27, 4.11)			1.40 (0.30, 6.64)			0.54 (0.10, 2.98)

*Time periods collapsed into weeks 0-4 and weeks 5-26.
Models could not be run for ICVT, mesenteric thrombus, portal vein thrombosis.

Supplementary Table 14: Hazard ratios and 95% CIs for individual arterial and venous thrombotic events, and other cardiovascular events, by time since booster vaccination with mRNA-1273 for those on a primary course of ChAdOx1.

Outcome		Age and Sex adjusted HR (95% CI)					Fully Adjusted HR (95% CI)				
		Week 1	Week 2	Weeks 3-4	Weeks 5-12	Weeks 13-24	Week 1	Week 2	Weeks 3-4	Weeks 5-12	Weeks 13-24
**Arterial**	Composite arterial*		0.64 (0.60,0.68)		0.53 (0.49,0.57)			0.77 (0.73, 0.82)		0.67 (0.62, 0.72)	
	AMI[2]*		0.67 (0.62,0.73)		0.58 (0.52,0.64)			0.79 (0.73, 0.86)		0.71 (0.64, 0.79)	
	Ischaemic stroke*		0.60 (0.54,0.66)		0.45 (0.40,0.51)			0.75 (0.68, 0.82)		0.61 (0.53, 0.69)	
**Venous**	Composite venous*		0.54 (0.48,0.61)		0.50 (0.43,0.59)			0.58 (0.51, 0.66)		0.55 (0.47, 0.65)	
	Lower limb DVT[7]*		0.60 (0.50,0.72)		0.54 (0.42,0.68)			0.64 (0.53, 0.77)		0.58 (0.46, 0.74)	
	Pulmonary embolism*		0.48 (0.40,0.57)		0.48 (0.39,0.59)			0.52 (0.44, 0.62)		0.54 (0.44, 0.66)	
	ICVT[8]*		0.86 (0.32,2.30)		0.82 (0.25,2.72)			0.92 (0.35, 2.43)		0.92 (0.29, 2.93)	
	Mesenteric thrombus*		0.45 (0.28,0.71)		0.36 (0.19,0.66)			0.52 (0.33, 0.82)		0.41 (0.22, 0.77)	
	Portal vein thrombosis										
**Other**	Any thrombocytopenia*		0.79 (0.49,1.27)		0.56 (0.27,1.18)			0.90 (0.56, 1.44)		0.63 (0.30, 1.32)	
	SAH and HS[9]	0.31 (0.17,0.58)	0.53 (0.33,0.86)	0.56 (0.39,0.81)	0.52 (0.36,0.74)	NA (NA, NA)					
	Myocarditis*		0.67 (0.31,1.46)		0.72 (0.24,2.16)			0.72 (0.33, 1.56)		0.79 (0.26, 2.36)	
	Pericarditis*		0.63 (0.24,1.60)		0.12 (0.01,0.95)			0.63 (0.25, 1.62)		0.12 (0.01, 0.98)	

*Time periods collapsed into weeks 0-4 and weeks 5-24.
Models could not be run for portal vein thrombosis and part of SAH and HS.
NA (NA-NA) Could not obtain estimate for this time interval

Supplementary Table 15: Hazard ratios and 95% CIs for individual arterial and venous thrombotic events, and other cardiovascular events, by time since booster vaccination with BNT-162b2 for those on a primary course of ChAdOx1.

**Outcome**		**Age and Sex adjusted HR (95% CI)**					**Fully Adjusted HR (95% CI)**				
		Week 1	Week 2	Weeks 3-4	Weeks 5-12	Weeks 13-24	Weeks 1	Week 2	Week 3-4	Weeks 5-12	Weeks 13-24
**Arterial**	Composite arterial	0.77 (0.73,0.80)	0.79 (0.75,0.83)	0.79 (0.76,0.82)	0.73 (0.70,0.75)	0.64 (0.60,0.69)	0.79 (0.75, 0.83)	0.82 (0.78, 0.86)	0.83 (0.80, 0.86)	0.78 (0.75, 0.81)	0.71 (0.66, 0.76)
	AMI[2]	0.76 (0.71,0.81)	0.81 (0.76,0.87)	0.79 (0.75,0.83)	0.73 (0.70,0.77)	0.62 (0.55,0.69)	0.76 (0.71, 0.82)	0.82 (0.76, 0.87)	0.80 (0.76, 0.84)	0.75 (0.72, 0.79)	0.63 (0.57, 0.71)
	Ischaemic stroke	0.78 (0.73,0.84)	0.78 (0.73,0.84)	0.80 (0.76,0.85)	0.74 (0.70,0.78)	0.69 (0.62,0.76)	0.82 (0.76, 0.88)	0.83 (0.77, 0.89)	0.86 (0.82, 0.91)	0.84 (0.80, 0.88)	0.81 (0.73, 0.90)
**Venous**	Composite venous	0.69 (0.62,0.77)	0.77 (0.69,0.85)	0.75 (0.69,0.81)	0.68 (0.63,0.73)	0.68 (0.58,0.80)	0.67 (0.60, 0.75)	0.74 (0.67, 0.83)	0.73 (0.67, 0.79)	0.64 (0.60, 0.69)	0.63 (0.54, 0.74)
	Lower limb DVT[7]	0.73 (0.61,0.86)	0.90 (0.77,1.05)	0.83 (0.74,0.94)	0.69 (0.62,0.78)	0.84 (0.66,1.07)	0.71 (0.60, 0.84)	0.87 (0.75, 1.02)	0.81 (0.71, 0.91)	0.66 (0.59, 0.74)	0.76 (0.60, 0.97)
	Pulmonary embolism	0.65 (0.57,0.75)	0.68 (0.59,0.78)	0.68 (0.61,0.76)	0.64 (0.58,0.70)	0.56 (0.46,0.70)	0.64 (0.55, 0.73)	0.66 (0.58, 0.76)	0.66 (0.60, 0.74)	0.61 (0.56, 0.67)	0.53 (0.43, 0.65)
	ICVT[8]*		0.75 (0.38,1.46)		0.86 (0.38,1.98)			0.72 (0.37,1.40)		0.76 (0.35,1.68)	
	Mesenteric thrombus	0.56 (0.38,0.81)	0.63 (0.44,0.90)	0.53 (0.39,0.71)	0.60 (0.47,0.76)	0.75 (0.48,1.17)	0.56 (0.38, 0.82)	0.64 (0.45, 0.92)	0.55 (0.41, 0.74)	0.65 (0.51, 0.82)	0.84 (0.53, 1.31)
	Portal vein thrombosis	0.68 (0.20,2.30)	0.22 (0.03,1.69)	0.57 (0.21,1.53)	0.69 (0.32,1.50)	0.79 (0.14,4.41)	0.64 (0.18, 2.32)	0.16 (0.02, 1.42)	0.42 (0.14, 1.26)	0.50 (0.21, 1.18)	0.58 (0.08, 4.19)
**Other**	Any thrombocytopenia	1.49 (1.08,2.06)	1.42 (1.01,2.00)	1.47 (1.11,1.94)	1.56 (1.19,2.05)	3.76 (2.19,6.47)	1.26 (0.92, 1.73)	1.17 (0.83, 1.64)	1.16 (0.88, 1.53)	1.09 (0.84, 1.41)	2.16 (1.26, 3.69)
	SAH and HS[9]	0.65 (0.53,0.81)	0.71 (0.58,0.87)	0.66 (0.57,0.78)	0.73 (0.64,0.83)	0.73 (0.55,0.98)	0.68 (0.55, 0.84)	0.74 (0.60, 0.91)	0.70 (0.60, 0.82)	0.80 (0.70, 0.91)	0.82 (0.61, 1.09)
	Myocarditis	1.42 (0.74,2.73)	1.05 (0.49,2.24)	0.97 (0.53,1.79)	0.75 (0.43,1.33)	0.38 (0.05,3.04)	1.32 (0.68, 2.56)	0.99 (0.46, 2.11)	0.90 (0.49, 1.65)	0.65 (0.37, 1.15)	0.30 (0.04, 2.36)
	Pericarditis	0.56 (0.20,1.53)	1.39 (0.69,2.81)	0.41 (0.17,0.96)	1.01 (0.58,1.76)	0.35 (0.04,2.81)	0.52 (0.19, 1.43)	1.26 (0.62, 2.55)	0.37 (0.16, 0.87)	0.82 (0.47, 1.42)	0.25 (0.03, 1.96)

*Time periods collapsed into weeks 0-4 and weeks 5-24.

Supplementary Table 16: Hazard ratios and 95% CIs for individual arterial and venous thrombotic events, and other cardiovascular events, by time since booster vaccination with mRNA-1273 or BNT-162b2 for those on a primary course of ChAdOx1.

Outcome		Age and Sex adjusted HR (95% CI)					Fully Adjusted HR (95% CI)				
		Week 1	Week 2	Weeks 3-4	Weeks 5-12	Weeks 13-24	Week 1	Week 2	Weeks 3-4	Weeks 5-12	Weeks 13-24
**Arterial**	Composite arterial	0.75 (0.72,0.79)	0.78 (0.74,0.82)	0.78 (0.75,0.81)	0.73 (0.70,0.75)	0.66 (0.62,0.71)	0.79 (0.75,0.83)	0.82 (0.79,0.86)	0.83 (0.80,0.86)	0.78 (0.76,0.81)	0.73 (0.67,0.78)
	AMI[2]	0.74 (0.70,0.79)	0.78 (0.74,0.84)	0.78 (0.74,0.82)	0.73 (0.70,0.76)	0.62 (0.56,0.69)	0.77 (0.72,0.82)	0.81 (0.76,0.86)	0.81 (0.77,0.85)	0.75 (0.72,0.79)	0.64 (0.58,0.71)
	Ischaemic stroke	0.77 (0.72,0.82)	0.77 (0.72,0.83)	0.78 (0.74,0.82)	0.73 (0.70,0.77)	0.71 (0.64,0.79)	0.82 (0.76,0.87)	0.83 (0.78,0.89)	0.84 (0.80,0.89)	0.82 (0.78,0.86)	0.81 (0.73,0.90)
**Venous**	Composite venous	0.66 (0.60,0.73)	0.76 (0.69,0.83)	0.71 (0.66,0.77)	0.67 (0.63,0.72)	0.67 (0.58,0.79)	0.66 (0.59,0.72)	0.75 (0.68,0.82)	0.70 (0.65,0.76)	0.64 (0.60,0.69)	0.63 (0.54,0.74)
	Lower limb DVT[7]	0.69 (0.59,0.80)	0.89 (0.77,1.03)	0.81 (0.72,0.92)	0.71 (0.64,0.80)	0.89 (0.70,1.13)	0.68 (0.58,0.80)	0.89 (0.77,1.03)	0.81 (0.72,0.91)	0.70 (0.63,0.78)	0.84 (0.66,1.06)
	Pulmonary embolism	0.63 (0.55,0.72)	0.66 (0.58,0.75)	0.64 (0.58,0.71)	0.64 (0.58,0.70)	0.57 (0.47,0.71)	0.63 (0.55,0.71)	0.66 (0.58,0.74)	0.64 (0.57,0.70)	0.62 (0.56,0.68)	0.55 (0.44,0.67)
	ICVT[8]*		0.78 (0.43,1.42)			0.83 (0.39,1.78)			0.76 (0.41,1.38)		0.78 (0.36,1.68)
	Mesenteric thrombus	0.51 (0.35,0.74)	0.55 (0.38,0.79)	0.58 (0.44,0.75)	0.59 (0.46,0.75)	0.83 (0.53,1.31)	0.52 (0.36,0.75)	0.56 (0.39,0.80)	0.60 (0.46,0.79)	0.61 (0.48,0.77)	0.85 (0.54,1.34)
	Portal vein thrombosis	0.62 (0.21,1.80)	0.45 (0.14,1.48)	0.58 (0.26,1.31)	0.44 (0.20,0.98)	0.45 (0.09,2.34)	0.61 (0.20,1.91)	0.45 (0.13,1.59)	0.68 (0.28,1.63)	0.42 (0.19,0.94)	0.27 (0.04,1.76)
**Other**	Any thrombocytopenia	1.33 (0.97,1.81)	1.30 (0.94,1.81)	1.44 (1.10,1.88)	1.53 (1.17,2.00)	3.87 (2.27,6.59)	1.14 (0.84,1.55)	1.09 (0.79,1.50)	1.14 (0.88,1.48)	1.05 (0.81,1.36)	2.17 (1.27,3.71)
	SAH and HS[9]	0.59 (0.49,0.73)	0.68 (0.56,0.82)	0.65 (0.56,0.76)	0.73 (0.64,0.83)	0.80 (0.60,1.07)	0.64 (0.52,0.78)	0.73 (0.61,0.89)	0.71 (0.61,0.83)	0.82 (0.72,0.94)	0.93 (0.70,1.24)
	Myocarditis	1.38 (0.77,2.46)	1.04 (0.53,2.04)	0.83 (0.45,1.52)	0.80 (0.46,1.39)	0.47 (0.06,3.75)	1.34 (0.76,2.37)	1.01 (0.52,1.97)	0.78 (0.43,1.43)	0.70 (0.41,1.21)	0.39 (0.05,3.07)
	Pericarditis	0.41 (0.15,1.13)	1.20 (0.61,2.36)	0.59 (0.30,1.14)	0.88 (0.50,1.55)	0.33 (0.04,2.63)	0.40 (0.14,1.10)	1.17 (0.60,2.28)	0.55 (0.29,1.07)	0.79 (0.46,1.37)	0.26 (0.03,1.98)

*Time periods collapsed into weeks 0-4 and weeks 5-24.

Supplementary Table 17: Hazard ratios and 95% CIs for individual arterial and venous thrombotic events, and other cardiovascular events, by time since booster vaccination with BNT-162b2 for those on a primary course of BNT-162b2.

		**Age and Sex adjusted HR (95% CI)**					**Fully Adjusted HR (95% CI)**				
**Outcome**		Week 1	Week 2	Weeks 3-4	Weeks 5-12	Weeks 13-24	Week 1	Week 2	Weeks 3-4	Weeks 5-12	Weeks 13-24
**Arterial**	Composite arterial	0.66 (0.63, 0.70)	0.74 (0.71, 0.78)	0.75 (0.72, 0.78)	0.70 (0.68, 0.73)	0.63 (0.60, 0.67)	0.69 (0.65, 0.73)	0.78 (0.74, 0.83)	0.80 (0.77, 0.83)	0.78 (0.75, 0.81)	0.73 (0.69, 0.77)
	AMI[2]	0.66 (0.61, 0.71)	0.76 (0.70, 0.81)	0.79 (0.75, 0.84)	0.72 (0.68, 0.75)	0.63 (0.58, 0.68)	0.68 (0.63, 0.74)	0.79 (0.73, 0.85)	0.84 (0.79, 0.89)	0.78 (0.75, 0.83)	0.71 (0.66, 0.77)
	Ischaemic stroke	0.67 (0.62, 0.72)	0.74 (0.69, 0.79)	0.71 (0.67, 0.75)	0.70 (0.66, 0.73)	0.65 (0.60, 0.70)	0.71 (0.65, 0.76)	0.79 (0.73, 0.85)	0.78 (0.73, 0.82)	0.80 (0.76, 0.84)	0.78 (0.72, 0.85)
**Venous**	Composite venous	0.63 (0.56, 0.71)	0.66 (0.59, 0.74)	0.64 (0.59, 0.70)	0.61 (0.56, 0.65)	0.54 (0.48, 0.61)	0.63 (0.56, 0.71)	0.66 (0.59, 0.74)	0.65 (0.59, 0.71)	0.62 (0.57, 0.66)	0.56 (0.49, 0.63)
	Lower limb DVT[7]	0.71 (0.59, 0.86)	0.77 (0.64, 0.91)	0.68 (0.59, 0.78)	0.63 (0.56, 0.71)	0.54 (0.44, 0.66)	0.71 (0.59, 0.86)	0.76 (0.64, 0.91)	0.68 (0.59, 0.78)	0.63 (0.56, 0.71)	0.55 (0.45, 0.67)
	Pulmonary embolism	0.56 (0.48, 0.66)	0.59 (0.51, 0.69)	0.62 (0.55, 0.70)	0.58 (0.53, 0.64)	0.54 (0.46, 0.63)	0.56 (0.48, 0.66)	0.59 (0.51, 0.69)	0.62 (0.55, 0.70)	0.59 (0.53, 0.65)	0.55 (0.47, 0.65)
	ICVT[8]*	0.50 (0.12, 2.11)	0.56 (0.13, 2.34)	0.74 (0.31, 1.78)	0.76 (0.41, 1.40)	0.83 (0.32, 2.13)	0.45 (0.11, 1.89)	0.54 (0.13, 2.30)	0.68 (0.28, 1.66)	0.70 (0.37, 1.31)	0.76 (0.29, 1.99)
	Mesenteric thrombus	0.48 (0.33, 0.71)	0.45 (0.30, 0.67)	0.66 (0.51, 0.85)	0.54 (0.43, 0.68)	0.41 (0.28, 0.60)	0.49 (0.33, 0.73)	0.46 (0.31, 0.68)	0.68 (0.53, 0.88)	0.56 (0.45, 0.71)	0.43 (0.29, 0.64)
	Portal vein thrombosis		0.25 (0.09, 0.72)		0.51 (0.23, 1.14)			0.27 (0.09, 0.80)		0.48 (0.19, 1.20)	
**Other**	Any thrombocytopenia	0.84 (0.57, 1.24)	0.56 (0.34, 0.91)	1.12 (0.84, 1.49)	0.86 (0.67, 1.11)	1.20 (0.80, 1.81)	0.80 (0.54, 1.18)	0.52 (0.32, 0.85)	1.04 (0.78, 1.37)	0.77 (0.60, 0.99)	1.06 (0.71, 1.60)
	SAH and HS[9]	0.71 (0.58, 0.88)	0.83 (0.68, 1.02)	0.85 (0.73, 0.99)	0.73 (0.63, 0.84)	0.63 (0.49, 0.79)	0.74 (0.60, 0.91)	0.87 (0.72, 1.07)	0.90 (0.77, 1.05)	0.80 (0.69, 0.92)	0.71 (0.56, 0.91)
	Myocarditis	1.83 (1.01, 3.32)	1.05 (0.49, 2.25)	1.24 (0.69, 2.23)	0.88 (0.53, 1.48)	0.34 (0.08, 1.50)	1.79 (0.98, 3.25)	1.03 (0.48, 2.22)	1.23 (0.68, 2.21)	0.86 (0.51, 1.44)	0.34 (0.08, 1.51)
	Pericarditis	0.98 (0.39, 2.47)	1.99 (0.97, 4.10)	1.17 (0.59, 2.31)	1.32 (0.78, 2.24)	1.33 (0.42, 4.23)	0.97 (0.38, 2.44)	1.99 (0.96, 4.09)	1.16 (0.58, 2.31)	1.30 (0.76, 2.21)	1.30 (0.41, 4.18)

*Time periods collapsed into weeks 0-4 and weeks 5-24.

Supplementary Table 18: Hazard ratios and 95% CIs for individual arterial and venous thrombotic events, and other cardiovascular events, by time since booster vaccination with mRNA-1273 for those on a primary course of BNT-162b2.

		Age and Sex adjusted HR (95% CI)					Fully Adjusted HR (95% CI)				
**Outcome**		Week 1	Week 2	Weeks 3-4	Weeks 5-12	Weeks 13-24	Week 1	Week 2	Weeks 3-4	Weeks 5-12	Weeks 13-24
**Arterial**	Composite arterial	0.63 (0.52, 0.78)	0.91 (0.77, 1.09)	0.82 (0.71, 0.94)	0.77 (0.69, 0.87)	0.88 (0.28, 2.79)	0.67 (0.55, 0.82)	0.97 (0.81, 1.15)	0.88 (0.77, 1.01)	0.85 (0.75, 0.96)	1.21 (0.38, 3.86)
	AMI[2]		0.86 (0.75, 0.98)		0.80 (0.68, 0.94)			0.90 (0.79, 1.04)		0.86 (0.73, 1.02)	
	Ischaemic stroke	0.46 (0.33, 0.65)	0.90 (0.70, 1.16)	0.75 (0.61, 0.93)	0.70 (0.59, 0.84)	NA (NA, NA)	0.48 (0.34, 0.68)	0.95 (0.74, 1.22)	0.80 (0.65, 0.98)	0.77 (0.64, 0.93)	2.29 (0.67, 7.82)
**Venous**	Composite venous		0.60 (0.49, 0.74)		0.56 (0.44, 0.72)			0.62 (0.50, 0.76)		0.58 (0.45, 0.74)	
	Lower limb DVT[7]		0.53 (0.39, 0.74)		0.48 (0.31, 0.72)			0.52 (0.38, 0.73)		0.46 (0.30, 0.69)	
	Pulmonary embolism		0.66 (0.50, 0.87)		0.67 (0.49, 0.92)			0.66 (0.50, 0.87)		0.67 (0.49, 0.92)	
	ICVT[8]*		0.28 (0.04, 1.86)		0.40 (0.05, 3.22)			0.28 (0.04, 1.87)		0.40 (0.05, 3.20)	
	Mesenteric thrombus		0.69 (0.36, 1.33)		0.68 (0.31, 1.45)			0.69 (0.35, 1.33)		0.66 (0.30, 1.44)	
	Portal vein thrombosis		0.65 (0.08, 5.17)		0.81 (0.08, 8.10)			0.66 (0.08, 5.19)		0.82 (0.08, 7.94)	
**Other**	Any thrombocytopenia		1.04 (0.59, 1.84)		0.13 (0.02, 0.98)			0.98 (0.56, 1.72)		0.11 (0.01, 0.80)	
	SAH and HS[9]		0.71 (0.46, 1.09)		0.64 (0.36, 1.14)			0.76 (0.49, 1.18)		0.70 (0.40, 1.25)	
	Myocarditis		0.92 (0.36, 2.34)		0.55 (0.08, 4.00)			0.87 (0.34, 2.21)		0.52 (0.07, 3.77)	
	Pericarditis		2.23 (0.71, 7.00)		1.06 (0.09, 12.46)			2.33 (0.74, 7.35)		1.08 (0.09, 12.70)	

*Time periods collapsed into weeks 0-4 and weeks 5-24.

Supplementary Table 19: Hazard ratios and 95% CIs for individual arterial and venous thrombotic events, and other cardiovascular events, by time since booster vaccination with mRNA-1273 or BNT-162b2 for those on a primary course of BNT-162b2.

		Age and Sex adjusted HR (95% CI)					Fully Adjusted HR (95% CI)				
**Outcome**		Week 1	Week 2	Weeks 3-4	Weeks 5-12	Weeks 13-24	Week 1	Week 2	Weeks 3-4	Weeks 5-12	Weeks 13-24
**Arterial**	Composite arterial	0.66 (0.62, 0.69)	0.75 (0.71, 0.79)	0.75 (0.72, 0.78)	0.70 (0.68, 0.73)	0.63 (0.59, 0.66)	0.69 (0.65, 0.73)	0.79 (0.75, 0.83)	0.80 (0.77, 0.84)	0.78 (0.75, 0.81)	0.73 (0.68, 0.77)
	AMI[2]	0.66 (0.62, 0.72)	0.76 (0.71, 0.82)	0.79 (0.75, 0.84)	0.71 (0.68, 0.75)	0.62 (0.58, 0.68)	0.69 (0.64, 0.74)	0.79 (0.74, 0.85)	0.83 (0.79, 0.88)	0.77 (0.73, 0.81)	0.70 (0.64, 0.76)
	Ischaemic stroke	0.65 (0.61, 0.70)	0.75 (0.69, 0.80)	0.71 (0.67, 0.75)	0.69 (0.66, 0.73)	0.65 (0.60, 0.70)	0.69 (0.64, 0.74)	0.80 (0.74, 0.85)	0.77 (0.73, 0.82)	0.79 (0.75, 0.83)	0.77 (0.71, 0.84)
**Venous**	Composite venous	0.62 (0.56, 0.70)	0.65 (0.58, 0.73)	0.64 (0.58, 0.69)	0.61 (0.56, 0.65)	0.54 (0.48, 0.61)	0.63 (0.56, 0.70)	0.66 (0.59, 0.73)	0.64 (0.59, 0.70)	0.61 (0.57, 0.66)	0.56 (0.49, 0.63)
	Lower limb DVT[7]	0.72 (0.60, 0.86)	0.75 (0.63, 0.89)	0.66 (0.58, 0.76)	0.64 (0.57, 0.72)	0.57 (0.47, 0.69)	0.72 (0.61, 0.86)	0.75 (0.63, 0.89)	0.66 (0.58, 0.76)	0.64 (0.57, 0.72)	0.59 (0.48, 0.72)
	Pulmonary embolism	0.56 (0.48, 0.65)	0.59 (0.51, 0.69)	0.63 (0.56, 0.70)	0.59 (0.53, 0.65)	0.53 (0.46, 0.62)	0.56 (0.48, 0.65)	0.60 (0.51, 0.69)	0.63 (0.56, 0.71)	0.59 (0.54, 0.65)	0.54 (0.46, 0.64)
	ICVT[8]*	0.43 (0.10, 1.81)	0.65 (0.20, 2.14)	0.63 (0.26, 1.55)	0.78 (0.42, 1.47)	0.93 (0.35, 2.47)	0.39 (0.09, 1.63)	0.61 (0.19, 1.98)	0.61 (0.25, 1.48)	0.69 (0.37, 1.29)	0.81 (0.30, 2.20)
	Mesenteric thrombus	0.46 (0.32, 0.68)	0.51 (0.36, 0.73)	0.64 (0.50, 0.82)	0.54 (0.43, 0.68)	0.42 (0.29, 0.61)	0.47 (0.32, 0.70)	0.53 (0.37, 0.76)	0.66 (0.51, 0.85)	0.57 (0.45, 0.72)	0.45 (0.31, 0.66)
	Portal vein thrombosis		0.28 (0.11, 0.74)		0.51 (0.23, 1.12)			0.24 (0.08, 0.68)		0.40 (0.17, 0.94)	
**Other**	Any thrombocytopenia	0.90 (0.63, 1.29)	0.65 (0.42, 1.01)	1.08 (0.82, 1.43)	0.85 (0.66, 1.09)	1.28 (0.85, 1.92)	0.84 (0.58, 1.20)	0.59 (0.38, 0.92)	0.97 (0.73, 1.27)	0.71 (0.55, 0.91)	1.02 (0.68, 1.54)
	SAH and HS[9]	0.73 (0.59, 0.89)	0.82 (0.67, 1.00)	0.87 (0.75, 1.01)	0.75 (0.65, 0.86)	0.66 (0.52, 0.84)	0.75 (0.61, 0.92)	0.85 (0.70, 1.04)	0.91 (0.78, 1.07)	0.81 (0.70, 0.93)	0.73 (0.58, 0.92)
	Myocarditis	1.87 (1.10, 3.18)	0.99 (0.48, 2.04)	1.07 (0.59, 1.94)	0.88 (0.53, 1.48)	0.37 (0.08, 1.64)	1.83 (1.07, 3.11)	0.96 (0.46, 1.98)	1.05 (0.58, 1.91)	0.85 (0.51, 1.43)	0.36 (0.08, 1.59)
	Pericarditis	0.88 (0.35, 2.18)	2.13 (1.09, 4.16)	1.29 (0.68, 2.43)	1.31 (0.78, 2.21)	1.45 (0.47, 4.51)	0.87 (0.35, 2.17)	2.14 (1.10, 4.17)	1.29 (0.68, 2.43)	1.33 (0.79, 2.24)	1.50 (0.48, 4.69)

*Time periods collapsed into weeks 0-4 and weeks 5-24.

Supplementary Table 20: Hazard ratios and 95% CIs for individual arterial and venous thrombotic events, and other cardiovascular events, by time since booster vaccination with mRNA-1273 for those on a primary course of mRNA-1273 or BNT-162b2.

Outcome		Age and Sex adjusted HR (95% CI)					Fully Adjusted HR (95% CI)				
		Week 1	Week 2	Weeks 3-4	Weeks 5-12	Weeks 13-24	Week 1	Week 2	Weeks 3-4	Weeks 5-12	Weeks 13-24
**Arterial**	Composite arterial	0.63 (0.51, 0.77)	0.92 (0.78, 1.10)	0.82 (0.71, 0.94)	0.79 (0.70, 0.89)	0.96 (0.30, 3.04)	0.66 (0.54, 0.81)	0.98 (0.83, 1.17)	0.88 (0.76, 1.01)	0.86 (0.76, 0.97)	1.04 (0.32, 3.38)
	AMI[2]*		0.84 (0.73, 0.96)		0.81 (0.69, 0.95)			0.89 (0.78, 1.02)		0.86 (0.73, 1.02)	
	Ischaemic stroke	0.46 (0.33, 0.64)	0.92 (0.72, 1.18)	0.77 (0.63, 0.94)	0.73 (0.61, 0.87)	NA (NA, NA)	0.49 (0.35, 0.68)	0.98 (0.77, 1.26)	0.83 (0.68, 1.02)	0.81 (0.67, 0.97)	1.80 (0.53, 6.14)
**Venous**	Composite venous*		0.62 (0.51, 0.76)		0.60 (0.47, 0.76)			0.61 (0.50, 0.75)		0.58 (0.45, 0.74)	
	Lower limb DVT[7]*		0.53 (0.38, 0.73)		0.52 (0.34, 0.78)			0.53 (0.38, 0.73)		0.51 (0.34, 0.76)	
	Pulmonary embolism*		0.67 (0.52, 0.88)		0.67 (0.49, 0.92)			0.70 (0.53, 0.91)		0.70 (0.51, 0.96)	
	ICVT[8]*		0.28 (0.04, 2.05)		0.36 (0.05, 2.81)			0.26 (0.04, 1.79)		0.27 (0.04, 1.94)	
	Mesenteric thrombus*		0.66 (0.34, 1.28)		0.67 (0.32, 1.43)			0.68 (0.35, 1.32)		0.68 (0.31, 1.46)	
	Portal vein thrombosis*		0.59 (0.08, 4.67)		0.66 (0.08, 5.28)			0.58 (0.07, 4.65)		0.64 (0.08, 5.08)	
**Other**	Any thrombocytopenia*		1.11 (0.64, 1.95)		0.14 (0.02, 1.02)			1.05 (0.59, 1.85)		0.12 (0.02, 0.90)	
	SAH and HS[9]*		0.67 (0.44, 1.03)		0.65 (0.36, 1.16)			0.72 (0.47, 1.11)		0.71 (0.40, 1.27)	
	Myocarditis*		0.94 (0.40, 2.19)		0.47 (0.06, 3.61)			0.90 (0.39, 2.11)		0.46 (0.06, 3.50)	
	Pericarditis*		2.02 (0.68, 6.03)		0.98 (0.10,10.16)			2.04 (0.68, 6.07)		1.00 (0.10,10.31)	

*Time periods collapsed into weeks 0-4 and weeks 5-24.

Supplementary Table 21: Hazard ratios and 95% CIs for individual arterial and venous thrombotic events, and other cardiovascular events, by time since booster vaccination with BNT-162b2 for those on a primary course of mRNA-1273 or BNT-162b2.

Outcome		Age and Sex adjusted HR (95% CI)					Fully Adjusted HR (95% CI)				
		Week 1	Week 2	Weeks 3-4	Weeks 5-12	Weeks 13-24	Week 1	Week 2	Weeks 3-4	Weeks 5-12	Weeks 13-24
**Arterial**	Composite arterial	0.66 (0.63,0.70)	0.75 (0.71,0.79)	0.76 (0.73, 0.79)	0.71 (0.69,0.74)	0.64 (0.60,0.68)	0.70 (0.66,0.74)	0.79 (0.75,0.84)	0.81 (0.78,0.85)	0.80 (0.77,0.83)	0.75 (0.70,0.79)
	AMI[2]	0.66 (0.61,0.72)	0.76 (0.71,0.82)	0.80 (0.76,0.85)	0.73 (0.69,0.77)	0.63 (0.59,0.69)	0.69 (0.64,0.74)	0.80 (0.74,0.86)	0.85 (0.80,0.90)	0.79 (0.75,0.83)	0.71 (0.65,0.77)
	Ischaemic stroke	0.67 (0.62,0.72)	0.74 (0.69,0.79)	0.71 (0.67,0.75)	0.70 (0.67,0.74)	0.65 (0.60,0.71)	0.71 (0.65,0.76)	0.79 (0.73,0.85)	0.77 (0.73,0.82)	0.80 (0.75,0.84)	0.77 (0.71,0.84)
**Venous**	Composite venous	0.63 (0.56,0.71)	0.67 (0.60,0.75)	0.65 (0.59,0.71)	0.61 (0.57,0.66)	0.55 (0.49,0.62)	0.63 (0.56,0.71)	0.67 (0.59,0.75)	0.64 (0.59,0.70)	0.61 (0.57,0.66)	0.56 (0.49,0.63)
	Lower limb DVT[7]	0.72 (0.60,0.86)	0.78 (0.65,0.93)	0.68 (0.59,0.79)	0.65 (0.58,0.73)	0.57 (0.46,0.69)	0.72 (0.60,0.86)	0.78 (0.65,0.93)	0.68 (0.59,0.79)	0.65 (0.58,0.73)	0.58 (0.48,0.71)
	Pulmonary embolism	0.57 (0.49,0.67)	0.60 (0.52,0.70)	0.63 (0.56,0.71)	0.59 (0.54,0.65)	0.55 (0.47,0.65)	0.58 (0.49,0.67)	0.61 (0.52,0.71)	0.64 (0.57,0.71)	0.60 (0.54,0.66)	0.56 (0.48,0.66)
	ICVT[8]	0.50 (0.12,2.09)	0.52 (0.12,2.16)	0.69 (0.29,1.67)	0.75 (0.41,1.38)	0.85 (0.34,2.17)	0.53 (0.13,2.19)	0.51 (0.12,2.15)	0.70 (0.29,1.68)	0.72 (0.39,1.34)	0.88 (0.34,2.26)
	Mesenteric thrombus	0.48 (0.33,0.71)	0.45 (0.31,0.67)	0.67 (0.52,0.87)	0.56 (0.45,0.70)	0.43 (0.29,0.63)	0.49 (0.33,0.72)	0.46 (0.31,0.69)	0.69 (0.53,0.89)	0.58 (0.46,0.73)	0.45 (0.31,0.67)
	Portal vein thrombosis*	0.23 (0.08,0.68)			0.45 (0.20,1.00)		0.21 (0.07,0.69)			0.38 (0.17,0.87)	
**Other**	Any thrombocytopenia	0.87 (0.59,1.28)	0.57 (0.35,0.93)	1.16 (0.87,1.53)	0.91 (0.71,1.17)	1.31 (0.87,1.98)	0.77 (0.52,1.13)	0.50 (0.30,0.81)	1.00 (0.75,1.32)	0.74 (0.57,0.95)	1.05 (0.69,1.59)
	SAH and HS[9]	0.72 (0.58,0.88)	0.84 (0.69,1.02)	0.85 (0.73,1.00)	0.73 (0.64,0.85)	0.63 (0.50,0.80)	0.74 (0.60,0.92)	0.88 (0.72,1.07)	0.90 (0.77,1.05)	0.80 (0.69,0.92)	0.71 (0.56,0.90)
	Myocarditis	1.88 (1.03,3.44)	1.06 (0.49,2.30)	1.32 (0.73,2.39)	1.04 (0.62,1.73)	0.43 (0.10,1.92)	1.93 (1.06,3.53)	1.11 (0.51,2.40)	1.38 (0.76,2.48)	1.06 (0.63,1.78)	0.45 (0.10,1.99)
	Pericarditis	0.98 (0.39,2.44)	1.92 (0.94,3.93)	1.14 (0.58,2.24)	1.32 (0.79,2.20)	1.39 (0.45,4.32)	0.96 (0.38,2.39)	1.83 (0.90,3.75)	1.10 (0.56,2.18)	1.28 (0.76,2.15)	1.33 (0.43,4.14)

*Time periods collapsed into weeks 0-4 and weeks 5-24.

Supplementary Table 22: Hazard ratios and 95% CIs for individual arterial and venous thrombotic events, and other cardiovascular events, by time since booster vaccination with mRNA-1273 or BNT-162b2 for those on a primary course of mRNA-1273 or BNT-162b2.

Outcome		Age and Sex adjusted HR (95% CI)					Fully Adjusted HR (95% CI)				
		Week 1	Week 2	Weeks 3-4	Weeks 5-12	Weeks 13-24	Week 1	Week 2	Weeks 3-4	Weeks 5-12	Weeks 13-24
**Arterial**	Composite arterial	0.66 (0.63,0.70)	0.76 (0.72,0.80)	0.76 (0.73,0.79)	0.71 (0.69,0.74)	0.64 (0.60,0.67)	0.69 (0.66,0.73)	0.80 (0.76,0.84)	0.81 (0.78,0.84)	0.79 (0.76,0.81)	0.73 (0.69,0.77)
	AMI[2]	0.67 (0.62,0.72)	0.77 (0.72,0.83)	0.80 (0.76,0.85)	0.72 (0.69,0.76)	0.63 (0.58,0.68)	0.70 (0.65,0.75)	0.81 (0.75,0.87)	0.85 (0.81,0.90)	0.80 (0.76,0.84)	0.71 (0.66,0.77)
	Ischaemic stroke	0.66 (0.61,0.71)	0.75 (0.70,0.81)	0.72 (0.68,0.76)	0.70 (0.67,0.74)	0.65 (0.60,0.71)	0.70 (0.65,0.75)	0.80 (0.75,0.86)	0.78 (0.74,0.83)	0.80 (0.76,0.84)	0.78 (0.72,0.84)
**Venous**	Composite venous	0.63 (0.56,0.71)	0.66 (0.59,0.74)	0.64 (0.59,0.70)	0.62 (0.57,0.66)	0.55 (0.49,0.63)	0.63 (0.56,0.71)	0.66 (0.59,0.74)	0.64 (0.59,0.70)	0.62 (0.57,0.67)	0.57 (0.50,0.64)
	Lower limb DVT[7]	0.72 (0.60,0.86)	0.76 (0.64,0.90)	0.67 (0.58,0.76)	0.65 (0.58,0.73)	0.57 (0.47,0.70)	0.72 (0.60,0.85)	0.76 (0.64,0.90)	0.66 (0.58,0.76)	0.65 (0.58,0.73)	0.58 (0.48,0.71)
	Pulmonary embolism	0.57 (0.49,0.66)	0.60 (0.52,0.69)	0.63 (0.56,0.70)	0.59 (0.53,0.64)	0.53 (0.46,0.62)	0.56 (0.48,0.66)	0.60 (0.52,0.69)	0.63 (0.56,0.70)	0.58 (0.53,0.64)	0.54 (0.46,0.63)
	ICVT[8]	0.39 (0.09,1.64)	0.61 (0.18,2.04)	0.56 (0.23,1.37)	0.71 (0.39,1.31)	0.88 (0.34,2.28)	0.42 (0.10,1.76)	0.61 (0.19,2.01)	0.60 (0.25,1.46)	0.77 (0.42,1.42)	0.91 (0.35,2.34)
	Mesenteric thrombus	0.46 (0.32,0.68)	0.51 (0.36,0.74)	0.65 (0.51,0.84)	0.56 (0.44,0.70)	0.43 (0.30,0.63)	0.48 (0.33,0.71)	0.54 (0.37,0.77)	0.69 (0.54,0.89)	0.61 (0.48,0.76)	0.49 (0.33,0.71)
	Portal vein thrombosis*	0.25 (0.10,0.65)			0.44 (0.20,0.94)		0.25 (0.10,0.66)			0.39 (0.17,0.88)	
**Other**	Any thrombocytopenia	0.93 (0.64,1.34)	0.67 (0.43,1.04)	1.14 (0.87,1.51)	0.91 (0.70,1.16)	1.39 (0.93,2.09)	0.85 (0.59,1.22)	0.60 (0.39,0.93)	1.02 (0.77,1.35)	0.76 (0.59,0.97)	1.10 (0.73,1.66)
	SAH and HS[9]	0.72 (0.58,0.88)	0.81 (0.67,0.99)	0.86 (0.74,1.00)	0.74 (0.64,0.86)	0.66 (0.52,0.83)	0.75 (0.61,0.93)	0.85 (0.70,1.04)	0.92 (0.79,1.07)	0.82 (0.71,0.95)	0.75 (0.60,0.96)
	Myocarditis	1.96 (1.18,3.25)	0.98 (0.47,2.04)	1.12 (0.62,2.01)	0.98 (0.59,1.62)	0.45 (0.10,1.97)	1.93 (1.16,3.21)	0.96 (0.46,2.00)	1.07 (0.59,1.96)	0.95 (0.57,1.58)	0.41 (0.09,1.80)
	Pericarditis	0.85 (0.34,2.10)	2.04 (1.06,3.94)	1.23 (0.66,2.32)	1.29 (0.78,2.15)	1.50 (0.49,4.61)	0.88 (0.35,2.20)	2.05 (1.07,3.93)	1.29 (0.69,2.42)	1.37 (0.82,2.28)	1.68 (0.54,5.21)

*Time periods collapsed into weeks 0-4 and weeks 5-24.

Supplementary Table 23: Hazard ratios and 95% CIs for individual arterial and venous thrombotic events, and other cardiovascular events, by time since booster vaccination with mRNA-1273 for those on a primary course of ChAdOx1 or mRNA-1273 or BNT-162b2.

Outcome		Age and Sex adjusted HR (95% CI)					Fully Adjusted HR (95% CI)				
		Week 1	Week 2	Weeks 3-4	Weeks 5-12	Weeks 13-24	Week 1	Week 2	Weeks 3-4	Weeks 5-12	Weeks 13-24
**Arterial**	Composite arterial	0.65 (0.59,0.71)	0.72 (0.66,0.79)	0.67 (0.62,0.72)	0.59 (0.56,0.64)	0.43 (0.14,1.35)	0.75 (0.68, 0.82)	0.84 (0.77, 0.92)	0.79 (0.74, 0.85)	0.73 (0.68, 0.78)	0.55 (0.18, 1.74)
	AMI[2]*	0.71 (0.66,0.76)			0.64 (0.59,0.70)		0.82 (0.76, 0.88)			0.75 (0.69, 0.82)	
	Ischaemic stroke	0.61 (0.53,0.70)	0.73 (0.64,0.84)	0.58 (0.52,0.65)	0.52 (0.47,0.57)	0.82 (0.26,2.59)	0.71 (0.61, 0.82)	0.86 (0.75, 0.99)	0.69 (0.62, 0.78)	0.65 (0.58, 0.72)	0.88 (0.27, 2.90)
**Venous**	Composite venous*	0.57 (0.52,0.64)			0.54 (0.48,0.62)		0.61 (0.55, 0.68)			0.58 (0.51, 0.66)	
	Lower limb DVT[7]*	0.59 (0.51,0.70)			0.55 (0.45,0.68)		0.62 (0.53, 0.73)			0.59 (0.48, 0.72)	
	Pulmonary embolism*	0.54 (0.47,0.62)			0.54 (0.46,0.65)		0.58 (0.50, 0.67)			0.59 (0.49, 0.70)	
	ICVT[8]*	0.72 (0.30,1.68)			0.69 (0.27,1.78)		0.74 (0.32, 1.75)			0.70 (0.27, 1.80)	
	Mesenteric thrombus*	0.48 (0.33,0.71)			0.44 (0.27,0.71)		0.54 (0.36, 0.79)			0.50 (0.31, 0.81)	
	Portal vein thrombosis	0.76 (0.34,1.71)			0.09 (0.01,0.76)		0.90 (0.40, 2.02)			0.10 (0.01, 0.79)	
**Other**	Any thrombocytopenia*	0.87 (0.61,1.25)			0.44 (0.23,0.82)		0.93 (0.65, 1.32)			0.45 (0.24, 0.84)	
	SAH and HS[9]	0.40 (0.25,0.65)	0.49 (0.31,0.76)	0.63 (0.46,0.86)	0.56 (0.42,0.76)	NA (NA, NA)	0.45 (0.28, 0.73)	0.55 (0.36, 0.87)	0.74 (0.54, 1.00)	0.68 (0.51, 0.92)	4.55 (0.59,35.00)
	Myocarditis*	0.84 (0.48,1.47)			0.75 (0.30,1.88)		0.86 (0.49, 1.51)			0.74 (0.29, 1.84)	
	Pericarditis*	0.97 (0.48,1.93)			0.30 (0.06,1.42)		1.01 (0.51, 2.02)			0.32 (0.07, 1.52)	

*Time periods collapsed into weeks 0-4 and weeks 5-24.
NA (NA-NA) Could not obtain estimate for this time interval

Supplementary Table 24: Hazard ratios and 95% CIs for individual arterial and venous thrombotic events, and other cardiovascular events, by time since booster vaccination with BNT-162b2 for those on a primary course of ChAdOx1 or mRNA-1273 or BNT-162b2.

Outcome		Age and Sex adjusted HR (95% CI)					Fully Adjusted HR (95% CI)				
		Week 1	Week 2	Weeks 3-4	Weeks 5-12	Weeks 13-24	Week 1	Week 2	Weeks 3-4	Weeks 5-12	Weeks 13-24
**Arterial**	Composite arterial	0.73 (0.70,0.75)	0.79 (0.76,0.81)	0.79 (0.77,0.81)	0.74 (0.72,0.76)	0.65 (0.63,0.68)	0.75 (0.72,0.78)	0.82 (0.79,0.85)	0.83 (0.81,0.86)	0.80 (0.78,0.82)	0.75 (0.72,0.78)
	AMI[2]	0.73 (0.69,0.77)	0.81 (0.77,0.85)	0.82 (0.79,0.85)	0.75 (0.73,0.78)	0.66 (0.62,0.70)	0.74 (0.70,0.77)	0.82 (0.78,0.86)	0.83 (0.80,0.87)	0.78 (0.76,0.81)	0.71 (0.67,0.75)
	Ischaemic stroke	0.73 (0.69,0.77)	0.77 (0.73,0.81)	0.77 (0.74,0.80)	0.73 (0.70,0.75)	0.66 (0.62,0.70)	0.77 (0.73,0.81)	0.82 (0.78,0.86)	0.83 (0.80,0.87)	0.84 (0.81,0.87)	0.82 (0.77,0.87)
**Venous**	Composite venous	0.67 (0.62,0.73)	0.73 (0.68,0.79)	0.72 (0.68,0.76)	0.66 (0.63,0.69)	0.60 (0.54,0.66)	0.66 (0.61,0.72)	0.72 (0.67,0.78)	0.71 (0.67,0.75)	0.65 (0.62,0.68)	0.60 (0.54,0.66)
	Lower limb DVT[7]	0.73 (0.65,0.83)	0.86 (0.77,0.96)	0.79 (0.72,0.86)	0.70 (0.65,0.76)	0.67 (0.57,0.77)	0.72 (0.64,0.82)	0.85 (0.76,0.96)	0.78 (0.71,0.86)	0.70 (0.64,0.75)	0.67 (0.58,0.78)
	Pulmonary embolism	0.63 (0.57,0.70)	0.66 (0.59,0.73)	0.67 (0.62,0.73)	0.63 (0.59,0.67)	0.56 (0.49,0.63)	0.62 (0.56,0.69)	0.65 (0.59,0.72)	0.66 (0.61,0.72)	0.62 (0.58,0.66)	0.55 (0.49,0.62)
	ICVT[8]	0.49 (0.18,1.35)	0.78 (0.34,1.77)	0.75 (0.40,1.42)	0.83 (0.51,1.33)	0.89 (0.37,2.17)	0.47 (0.17,1.29)	0.73 (0.32,1.65)	0.68 (0.36,1.29)	0.71 (0.43,1.15)	0.72 (0.29,1.75)
	Mesenteric thrombus	0.52 (0.40,0.68)	0.54 (0.42,0.71)	0.63 (0.52,0.76)	0.63 (0.54,0.74)	0.56 (0.42,0.74)	0.52 (0.40,0.68)	0.55 (0.42,0.71)	0.64 (0.53,0.77)	0.65 (0.55,0.76)	0.60 (0.45,0.79)
	Portal vein thrombosis	0.57 (0.23,1.40)	0.12 (0.02,0.86)	0.39 (0.17,0.87)	0.57 (0.33,0.97)	0.29 (0.08,1.06)	0.57 (0.22,1.47)	0.10 (0.01,0.76)	0.39 (0.17,0.92)	0.62 (0.34,1.14)	0.37 (0.09,1.52)
**Other**	Any thrombocytopenia	1.16 (0.91,1.49)	0.98 (0.74,1.28)	1.30 (1.07,1.58)	1.18 (0.99,1.41)	2.00 (1.46,2.75)	0.99 (0.78,1.27)	0.82 (0.62,1.07)	1.06 (0.87,1.28)	0.88 (0.74,1.05)	1.40 (1.02,1.92)
	SAH and HS[9]	0.69 (0.60,0.80)	0.78 (0.68,0.90)	0.77 (0.69,0.86)	0.75 (0.68,0.82)	0.65 (0.55,0.78)	0.72 (0.62,0.84)	0.82 (0.72,0.95)	0.82 (0.74,0.92)	0.83 (0.75,0.91)	0.76 (0.64,0.91)
	Myocarditis	1.73 (1.12,2.69)	1.10 (0.64,1.89)	1.18 (0.77,1.79)	0.94 (0.65,1.36)	0.42 (0.13,1.39)	1.65 (1.07,2.57)	1.06 (0.62,1.82)	1.11 (0.73,1.69)	0.85 (0.59,1.24)	0.37 (0.11,1.24)
	Pericarditis	0.79 (0.40,1.55)	1.75 (1.07,2.87)	0.77 (0.45,1.30)	1.27 (0.88,1.83)	0.87 (0.34,2.20)	0.78 (0.40,1.54)	1.73 (1.05,2.83)	0.76 (0.44,1.28)	1.22 (0.85,1.76)	0.85 (0.34,2.14)

Supplementary Table 25: Hazard ratios and 95% CIs for individual arterial and venous thrombotic events, and other cardiovascular events, by time since booster vaccination with mRNA-1273 or BNT-162b2 for those on a primary course of ChAdOx1 or mRNA-1273 or BNT-162b2.

**Outcome**		**Age and Sex adjusted HR (95% CI)**					**Fully Adjusted HR (95% CI)**				
		Week 1	Week 2	Weeks 3-4	Weeks 5-12	Weeks 13-24	Week 1	Week 2	Weeks 3-4	Weeks 5-12	Weeks 13-24
**Arterial thrombotic events**	Composite arterial	0.72 (0.69,0.74)	0.78 (0.75,0.81)	0.78 (0.76,0.80)	0.73 (0.72,0.75)	0.66 (0.63,0.69)	0.75 (0.72,0.77)	0.82 (0.79,0.84)	0.82 (0.80,0.85)	0.79 (0.77,0.81)	0.74 (0.71,0.77)
	AMI[2]	0.72 (0.69,0.76)	0.79 (0.75,0.83)	0.80 (0.78,0.83)	0.75 (0.72,0.77)	0.66 (0.62,0.70)	0.74 (0.71,0.78)	0.82 (0.78,0.85)	0.83 (0.80,0.86)	0.78 (0.75,0.80)	0.70 (0.66,0.74)
	Ischaemic stroke	0.72 (0.68,0.75)	0.77 (0.73,0.81)	0.75 (0.73,0.78)	0.73 (0.70,0.75)	0.68 (0.64,0.72)	0.76 (0.73,0.80)	0.82 (0.78,0.86)	0.82 (0.79,0.85)	0.82 (0.79,0.85)	0.80 (0.75,0.85)
**Venous thromboembolic events**	Composite venous	0.66 (0.61,0.71)	0.73 (0.68,0.78)	0.70 (0.66,0.74)	0.67 (0.64,0.70)	0.61 (0.56,0.67)	0.66 (0.61,0.71)	0.72 (0.67,0.78)	0.69 (0.66,0.73)	0.65 (0.62,0.69)	0.60 (0.55,0.66)
	Lower limb DVT[7]	0.70 (0.63,0.79)	0.84 (0.76,0.94)	0.77 (0.70,0.84)	0.70 (0.65,0.76)	0.68 (0.59,0.79)	0.70 (0.62,0.79)	0.84 (0.75,0.94)	0.76 (0.70,0.83)	0.69 (0.64,0.75)	0.67 (0.58,0.78)
	Pulmonary embolism	0.62 (0.56,0.68)	0.65 (0.59,0.71)	0.65 (0.61,0.70)	0.63 (0.59,0.67)	0.56 (0.50,0.63)	0.62 (0.56,0.68)	0.65 (0.59,0.71)	0.65 (0.61,0.70)	0.62 (0.58,0.66)	0.56 (0.50,0.63)
	ICVT[8]	0.59 (0.25,1.35)	0.92 (0.47,1.83)	0.62 (0.33,1.16)	0.81 (0.51,1.29)	0.86 (0.35,2.08)	0.54 (0.23,1.24)	0.84 (0.42,1.66)	0.55 (0.29,1.02)	0.66 (0.42,1.06)	0.68 (0.28,1.67)
	Mesenteric thrombus	0.50 (0.38,0.65)	0.55 (0.43,0.71)	0.65 (0.54,0.78)	0.63 (0.54,0.74)	0.58 (0.44,0.76)	0.51 (0.39,0.66)	0.56 (0.44,0.73)	0.67 (0.56,0.80)	0.65 (0.56,0.77)	0.62 (0.46,0.82)
	Portal vein thrombosis	0.55 (0.24,1.27)	0.27 (0.08,0.87)	0.50 (0.26,0.97)	0.53 (0.32,0.90)	0.31 (0.08,1.15)	0.51 (0.21,1.23)	0.27 (0.08,0.88)	0.47 (0.23,0.93)	0.43 (0.25,0.73)	0.24 (0.06,1.01)
**Other cardiovascular events**	Any thrombocytopenia	1.13 (0.89,1.42)	0.98 (0.76,1.27)	1.29 (1.06,1.55)	1.19 (1.00,1.41)	2.21 (1.61,3.03)	1.01 (0.80,1.28)	0.86 (0.67,1.11)	1.11 (0.92,1.33)	0.93 (0.78,1.10)	1.57 (1.15,2.15)
	SAH and HS[9]	0.66 (0.57,0.76)	0.75 (0.65,0.86)	0.76 (0.68,0.84)	0.74 (0.68,0.82)	0.68 (0.57,0.80)	0.69 (0.59,0.79)	0.79 (0.69,0.90)	0.80 (0.72,0.89)	0.81 (0.74,0.89)	0.77 (0.64,0.91)
	Myocarditis	1.71 (1.17,2.50)	1.04 (0.64,1.70)	0.96 (0.63,1.46)	0.91 (0.63,1.30)	0.41 (0.12,1.36)	1.69 (1.15,2.47)	1.05 (0.64,1.71)	0.95 (0.63,1.45)	0.87 (0.60,1.24)	0.40 (0.12,1.34)
	Pericarditis	0.64 (0.32,1.26)	1.69 (1.05,2.70)	0.94 (0.60,1.50)	1.27 (0.88,1.84)	1.02 (0.40,2.59)	0.61 (0.31,1.20)	1.60 (1.00,2.54)	0.88 (0.56,1.39)	1.11 (0.77,1.59)	0.83 (0.33,2.10)

Supplementary Table 26: Subgroup-specific maximally-adjusted hazard ratios and 95% CIs for composite arterial and composite venous events by time since first dose vaccination.

Vaccination course	Event	Stratum	Week 1	Week 2	Weeks 3-4	Weeks 5-12	Weeks 13-24	Weeks 25-26
Dose 1: BNT-162b2	Composite arterial	Age group: <40	0.97 (0.72,1.30)	0.94 (0.70,1.27)	0.90 (0.72,1.13)	1.16 (1.02,1.32)	1.00 (0.87,1.15)	0.93 (0.72,1.21)
		Age group: 40-59	0.87 (0.78,0.99)	0.99 (0.88,1.11)	1.00 (0.92,1.09)	0.91 (0.85,0.96)	0.94 (0.88,1.00)	0.99 (0.89,1.10)
		Age group: 60-79	0.75 (0.70,0.81)	0.84 (0.79,0.90)	0.86 (0.81,0.90)	0.88 (0.84,0.92)	0.94 (0.90,0.98)	0.96 (0.90,1.03)
		Age group: >=80	0.62 (0.58,0.67)	0.67 (0.62,0.72)	0.72 (0.68,0.77)	0.77 (0.73,0.80)	0.85 (0.81,0.90)	0.89 (0.82,0.96)
		Ethnicity: White	0.72 (0.68,0.75)	0.79 (0.76,0.83)	0.81 (0.78,0.84)	0.82 (0.80,0.84)	0.86 (0.83,0.88)	0.85 (0.81,0.88)
		Ethnicity: Black or Black British	0.51 (0.34,0.78)	0.71 (0.50,1.02)	0.95 (0.76,1.20)	0.92 (0.79,1.07)	0.84 (0.73,0.97)	0.83 (0.62,1.10)
		Ethnicity: Asian or Asian British	0.79 (0.65,0.96)	0.97 (0.81,1.16)	0.99 (0.87,1.13)	0.99 (0.90,1.08)	1.07 (0.98,1.17)	1.25 (1.07,1.47)
		Ethnicity: Other Ethnic Groups	0.79 (0.49,1.27)	0.54 (0.30,0.95)	0.92 (0.66,1.28)	0.96 (0.77,1.21)	1.14 (0.92,1.42)	1.14 (0.77,1.68)
		Ethnicity: Mixed	0.90 (0.51,1.56)	1.08 (0.63,1.84)	1.02 (0.68,1.53)	0.90 (0.69,1.17)	0.80 (0.61,1.06)	0.92 (0.56,1.54)
		Ethnicity: Unknown	1.01 (0.63,1.63)	0.82 (0.47,1.42)	1.20 (0.86,1.69)	1.40 (1.12,1.75)	1.99 (1.59,2.49)	1.73 (1.17,2.56)
		No prior history of COVID-19	0.73 (0.70,0.77)	0.82 (0.78,0.86)	0.84 (0.81,0.87)	0.86 (0.84,0.88)	0.91 (0.89,0.94)	0.92 (0.88,0.96)
		Prior history of COVID-19	0.63 (0.48,0.83)	0.53 (0.39,0.73)	0.77 (0.63,0.94)	0.78 (0.68,0.90)	0.89 (0.75,1.04)	0.79 (0.60,1.05)
		No prior history of event	0.70 (0.66,0.75)	0.80 (0.76,0.86)	0.86 (0.82,0.90)	0.89 (0.86,0.92)	0.94 (0.91,0.97)	0.97 (0.92,1.02)
		Prior history of event	0.76 (0.71,0.81)	0.82 (0.77,0.87)	0.83 (0.79,0.87)	0.84 (0.81,0.87)	0.95 (0.92,0.99)	0.99 (0.93,1.05)
		Sex: Female	0.68 (0.63,0.73)	0.74 (0.69,0.80)	0.79 (0.75,0.83)	0.82 (0.79,0.85)	0.83 (0.80,0.87)	0.83 (0.78,0.89)
		Sex: Male	0.78 (0.73,0.82)	0.87 (0.82,0.92)	0.89 (0.85,0.93)	0.91 (0.88,0.94)	0.99 (0.96,1.02)	1.01 (0.96,1.06)
	Composite venous	Age group: <40	0.66 (0.50,0.88)	0.85 (0.66,1.09)	0.75 (0.62,0.91)	0.70 (0.62,0.78)	0.65 (0.58,0.72)	0.53 (0.42,0.66)
		Age group: 40-59	0.77 (0.60,0.97)	0.94 (0.76,1.17)	0.91 (0.77,1.07)	0.92 (0.84,1.02)	0.92 (0.84,1.01)	0.84 (0.70,1.02)
		Age group: 60-79	0.66 (0.57,0.78)	0.74 (0.64,0.86)	0.62 (0.55,0.70)	0.68 (0.63,0.75)	0.90 (0.82,0.98)	0.87 (0.75,1.01)
		Age group: >=80	0.50 (0.41,0.61)	0.58 (0.48,0.71)	0.62 (0.54,0.72)	0.61 (0.54,0.69)	0.84 (0.75,0.95)	0.92 (0.77,1.11)
		Ethnicity: White	0.65 (0.59,0.72)	0.76 (0.69,0.84)	0.73 (0.68,0.79)	0.76 (0.73,0.80)	0.80 (0.77,0.84)	0.75 (0.69,0.82)
		Ethnicity: Black or Black British	0.89 (0.49,1.63)	1.13 (0.66,1.93)	0.77 (0.48,1.22)	0.92 (0.72,1.17)	0.95 (0.77,1.18)	0.89 (0.56,1.41)
		Ethnicity: Asian or Asian British	0.51 (0.25,1.04)	1.02 (0.60,1.73)	1.18 (0.83,1.69)	0.96 (0.76,1.21)	1.07 (0.86,1.34)	0.56 (0.31,0.98)
		Ethnicity: Other Ethnic Groups	1.03 (0.40,2.64)	1.42 (0.63,3.18)	0.59 (0.24,1.47)	0.89 (0.57,1.39)	1.08 (0.74,1.57)	1.10 (0.51,2.38)
		Ethnicity: Mixed	0.63 (0.20,1.99)	0.88 (0.32,2.44)	0.68 (0.30,1.57)	0.62 (0.39,0.99)	0.61 (0.39,0.93)	0.55 (0.23,1.32)
		Ethnicity: Unknown	0.98 (0.35,2.71)	0.55 (0.13,2.29)	1.30 (0.64,2.64)	1.42 (0.92,2.17)	1.80 (1.21,2.69)	1.48 (0.61,3.56)
		No prior history of COVID-19	0.68 (0.62,0.76)	0.80 (0.73,0.88)	0.76 (0.71,0.82)	0.80 (0.77,0.84)	0.86 (0.82,0.90)	0.79 (0.73,0.86)
		Prior history of COVID-19	0.32 (0.15,0.68)	0.63 (0.36,1.10)	0.80 (0.55,1.17)	0.66 (0.51,0.86)	0.72 (0.55,0.95)	0.62 (0.36,1.07)
		No prior history of event	0.66 (0.59,0.73)	0.80 (0.73,0.89)	0.76 (0.70,0.81)	0.78 (0.74,0.82)	0.84 (0.81,0.88)	0.75 (0.69,0.82)
		Prior history of event	0.68 (0.53,0.88)	0.69 (0.53,0.89)	0.71 (0.59,0.85)	0.77 (0.69,0.86)	0.77 (0.69,0.86)	0.87 (0.72,1.06)
		Sex: Female	0.71 (0.62,0.81)	0.73 (0.64,0.84)	0.73 (0.66,0.81)	0.78 (0.74,0.83)	0.82 (0.77,0.87)	0.76 (0.68,0.84)
		Sex: Male	0.63 (0.54,0.74)	0.88 (0.77,1.01)	0.81 (0.73,0.89)	0.82 (0.77,0.88)	0.90 (0.84,0.96)	0.82 (0.73,0.92)
Dose 1: ChAdOx1	Composite arterial	Age group: <40	0.97 (0.67,1.39)	1.11 (0.79,1.57)	1.10 (0.84,1.42)	1.24 (1.05,1.48)	0.99 (0.83,1.18)	1.08 (0.80,1.45)
		Age group: 40-59	0.82 (0.76,0.89)	1.04 (0.96,1.12)	1.03 (0.97,1.09)	0.98 (0.93,1.02)	1.00 (0.95,1.05)	1.01 (0.93,1.08)
		Age group: 60-79	0.82 (0.77,0.86)	0.90 (0.86,0.96)	0.91 (0.87,0.95)	0.94 (0.91,0.98)	0.97 (0.94,1.01)	1.00 (0.95,1.06)
		Age group: >=80	0.73 (0.67,0.80)	0.83 (0.76,0.91)	0.85 (0.79,0.92)	0.90 (0.85,0.95)	0.97 (0.91,1.03)	0.95 (0.87,1.04)
		Ethnicity: White	0.81 (0.77,0.84)	0.94 (0.90,0.98)	0.93 (0.90,0.96)	0.94 (0.92,0.97)	0.94 (0.91,0.96)	0.92 (0.89,0.96)
		Ethnicity: Black or Black British	0.98 (0.76,1.27)	1.13 (0.89,1.45)	0.99 (0.81,1.20)	1.08 (0.95,1.22)	0.93 (0.82,1.06)	0.82 (0.65,1.04)
		Ethnicity: Asian or Asian British	0.99 (0.84,1.16)	1.09 (0.93,1.27)	1.23 (1.09,1.38)	1.24 (1.14,1.35)	1.27 (1.16,1.39)	1.19 (1.03,1.37)
		Ethnicity: Other Ethnic Groups	1.21 (0.85,1.70)	1.17 (0.82,1.67)	1.06 (0.80,1.41)	1.22 (1.01,1.47)	1.16 (0.95,1.41)	1.31 (0.95,1.80)
		Ethnicity: Mixed	0.93 (0.58,1.51)	0.96 (0.59,1.56)	1.00 (0.70,1.45)	1.00 (0.78,1.27)	1.25 (0.98,1.59)	1.38 (0.92,2.09)
		Ethnicity: Unknown	1.10 (0.74,1.64)	0.95 (0.62,1.45)	1.94 (1.51,2.49)	1.94 (1.62,2.33)	1.99 (1.65,2.40)	2.49 (1.86,3.33)
		No prior history of COVID-19	0.83 (0.80,0.86)	0.97 (0.93,1.01)	0.97 (0.94,1.00)	1.00 (0.98,1.02)	1.00 (0.97,1.02)	0.99 (0.95,1.03)
		Prior history of COVID-19	0.93 (0.78,1.13)	0.83 (0.68,1.02)	0.87 (0.74,1.02)	0.83 (0.73,0.95)	0.82 (0.70,0.95)	0.87 (0.69,1.10)
		No prior history of event	0.85 (0.81,0.90)	1.04 (0.99,1.10)	1.05 (1.01,1.09)	1.04 (1.01,1.07)	1.04 (1.01,1.07)	1.03 (0.99,1.08)
		Prior history of event	0.81 (0.76,0.86)	0.87 (0.82,0.92)	0.86 (0.82,0.90)	0.93 (0.90,0.96)	0.97 (0.93,1.01)	0.99 (0.93,1.05)
		Sex: Female	0.78 (0.73,0.84)	0.91 (0.86,0.97)	0.90 (0.86,0.95)	0.91 (0.88,0.95)	0.90 (0.86,0.94)	0.86 (0.81,0.92)
		Sex: Male	0.87 (0.83,0.92)	1.00 (0.96,1.06)	1.02 (0.98,1.06)	1.06 (1.03,1.09)	1.08 (1.05,1.12)	1.11 (1.05,1.16)
	Composite venous	Age group: <40	1.17 (0.87,1.59)	2.02 (1.59,2.58)	1.33 (1.08,1.65)	1.09 (0.95,1.24)	0.90 (0.79,1.02)	0.70 (0.54,0.90)
		Age group: 40-59	0.64 (0.55,0.76)	1.12 (0.99,1.28)	1.14 (1.03,1.26)	0.95 (0.89,1.03)	0.94 (0.87,1.01)	0.86 (0.75,0.97)
		Age group: 60-79	0.76 (0.68,0.86)	0.83 (0.74,0.92)	0.74 (0.68,0.82)	0.78 (0.72,0.84)	0.83 (0.74,0.94)	0.76 (0.68,0.86)
		Age group: >=80	0.78 (0.64,0.97)	0.70 (0.56,0.88)	0.75 (0.63,0.90)	0.81 (0.70,0.94)	1.03 (0.90,1.19)	1.06 (0.85,1.32)
		Ethnicity: White	0.80 (0.73,0.87)	1.01 (0.93,1.09)	0.97 (0.91,1.03)	0.90 (0.86,0.94)	0.92 (0.88,0.97)	0.83 (0.77,0.89)
		Ethnicity: Black or Black British	0.93 (0.56,1.57)	1.06 (0.65,1.73)	1.26 (0.89,1.78)	1.17 (0.94,1.44)	0.87 (0.71,1.07)	0.86 (0.58,1.27)
		Ethnicity: Asian or Asian British	0.83 (0.50,1.38)	0.85 (0.51,1.43)	0.87 (0.60,1.26)	1.05 (0.84,1.31)	1.03 (0.82,1.31)	0.62 (0.38,1.03)
		Ethnicity: Other Ethnic Groups	0.69 (0.24,1.97)	1.58 (0.75,3.33)	0.85 (0.43,1.69)	0.96 (0.66,1.41)	0.91 (0.63,1.31)	0.30 (0.10,0.84)
		Ethnicity: Mixed	0.40 (0.10,1.64)	1.15 (0.50,2.66)	0.29 (0.09,0.92)	0.84 (0.56,1.27)	0.72 (0.49,1.06)	1.18 (0.64,2.19)
		Ethnicity: Unknown	1.39 (0.67,2.85)	1.07 (0.46,2.50)	1.91 (1.15,3.16)	1.58 (1.12,2.22)	2.11 (1.50,2.98)	2.21 (1.24,3.93)
		No prior history of COVID-19	0.80 (0.73,0.87)	1.01 (0.94,1.09)	0.97 (0.92,1.03)	0.91 (0.88,0.95)	0.93 (0.89,0.98)	0.83 (0.78,0.90)
		Prior history of COVID-19	0.89 (0.59,1.33)	1.10 (0.75,1.61)	1.09 (0.81,1.46)	1.04 (0.84,1.30)	1.04 (0.81,1.33)	0.88 (0.57,1.36)
		No prior history of event	0.83 (0.76,0.90)	1.02 (0.94,1.11)	0.96 (0.91,1.03)	0.93 (0.89,0.97)	0.94 (0.90,0.99)	0.82 (0.76,0.88)
		Prior history of event	0.64 (0.51,0.80)	0.93 (0.77,1.12)	0.99 (0.86,1.14)	0.78 (0.71,0.87)	0.83 (0.74,0.92)	0.86 (0.73,1.03)
		Sex: Female	0.82 (0.73,0.92)	1.01 (0.91,1.12)	0.97 (0.89,1.05)	0.88 (0.83,0.93)	0.90 (0.85,0.96)	0.83 (0.75,0.92)
		Sex: Male	0.81 (0.72,0.91)	1.05 (0.95,1.17)	1.03 (0.95,1.12)	1.01 (0.95,1.07)	1.04 (0.97,1.10)	0.91 (0.82,1.00)

Supplementary Table 27: Subgroup-specific maximally-adjusted hazard ratios and 95% CIs for composite arterial and composite venous events by time since COVID-19 dose 1&2 vaccination.

Vaccination course	Event	Stratum	Week 1	Week 2	Weeks 3-4	Weeks 5-12	Weeks 13-24	Weeks 25-26
**Doses 1&2: BNT-162b2**	Composite arterial	Age group: <40	1.02 (0.73,1.42)	1.22 (0.89,1.66)	0.85 (0.65,1.13)	1.03 (0.84,1.27)	1.11 (0.86,1.43)	1.33 (0.88,2.00)
		Age group: 40-59	0.73 (0.64,0.83)	0.82 (0.72,0.93)	0.85 (0.77,0.94)	0.78 (0.72,0.84)	0.70 (0.64,0.77)	0.63 (0.55,0.72)
		Age group: 60-79	0.72 (0.67,0.78)	0.82 (0.76,0.88)	0.77 (0.73,0.83)	0.70 (0.66,0.75)	0.65 (0.60,0.70)	0.62 (0.57,0.68)
		Age group: >=80	0.72 (0.67,0.78)	0.79 (0.73,0.85)	0.84 (0.79,0.89)	0.86 (0.82,0.90)	0.83 (0.78,0.88)	0.82 (0.75,0.89)
		Ethnicity: White	0.72 (0.69,0.76)	0.81 (0.78,0.86)	0.81 (0.78,0.85)	0.81 (0.78,0.84)	0.81 (0.78,0.84)	0.81 (0.76,0.86)
		Ethnicity: Black or Black British	0.71 (0.48,1.05)	0.99 (0.70,1.40)	0.86 (0.65,1.14)	0.73 (0.58,0.91)	0.73 (0.57,0.92)	0.71 (0.49,1.05)
		Ethnicity: Asian or Asian British	0.75 (0.62,0.92)	0.84 (0.69,1.02)	0.91 (0.78,1.05)	0.82 (0.73,0.92)	0.74 (0.65,0.85)	0.75 (0.62,0.92)
		Ethnicity: Other Ethnic Groups	1.08 (0.70,1.66)	0.94 (0.59,1.48)	0.77 (0.52,1.13)	0.80 (0.60,1.06)	0.72 (0.52,0.99)	0.82 (0.50,1.35)
		Ethnicity: Mixed	0.51 (0.25,1.04)	0.27 (0.10,0.68)	0.56 (0.33,0.95)	0.71 (0.47,1.05)	0.76 (0.50,1.16)	0.86 (0.43,1.73)
		Ethnicity: Unknown	0.65 (0.39,1.08)	0.59 (0.34,1.03)	0.74 (0.50,1.10)	0.84 (0.61,1.14)	0.60 (0.41,0.86)	0.42 (0.24,0.75)
		No prior history of COVID-19	0.72 (0.69,0.76)	0.81 (0.77,0.85)	0.81 (0.78,0.84)	0.80 (0.78,0.83)	0.79 (0.76,0.82)	0.79 (0.75,0.84)
		Prior history of COVID-19	0.73 (0.57,0.93)	0.93 (0.74,1.16)	0.87 (0.72,1.05)	0.88 (0.76,1.03)	0.81 (0.67,0.96)	0.81 (0.62,1.06)
		No prior history of event	0.70 (0.65,0.75)	0.84 (0.79,0.90)	0.83 (0.79,0.88)	0.83 (0.79,0.86)	0.80 (0.76,0.84)	0.76 (0.71,0.81)
		Prior history of event	0.77 (0.72,0.82)	0.80 (0.75,0.86)	0.82 (0.78,0.86)	0.78 (0.74,0.81)	0.74 (0.70,0.78)	0.78 (0.72,0.84)
		Sex: Female	0.69 (0.64,0.75)	0.79 (0.73,0.85)	0.81 (0.76,0.86)	0.77 (0.73,0.81)	0.74 (0.69,0.78)	0.75 (0.69,0.81)
		Sex: Male	0.76 (0.71,0.81)	0.85 (0.80,0.90)	0.84 (0.80,0.88)	0.86 (0.83,0.90)	0.89 (0.85,0.94)	0.90 (0.84,0.97)
	Composite venous	Age group: <40	0.76 (0.56,1.03)	0.92 (0.69,1.22)	0.60 (0.46,0.77)	0.74 (0.63,0.87)	0.78 (0.66,0.94)	0.76 (0.54,1.07)
		Age group: 40-59	0.79 (0.62,1.02)	0.93 (0.73,1.17)	0.79 (0.65,0.95)	0.77 (0.67,0.88)	0.71 (0.62,0.83)	0.67 (0.54,0.85)
		Age group: 60-79	0.74 (0.62,0.88)	0.73 (0.61,0.87)	0.68 (0.58,0.79)	0.64 (0.55,0.75)	0.64 (0.54,0.76)	0.61 (0.49,0.75)
		Age group: >=80	0.61 (0.50,0.75)	0.72 (0.60,0.87)	0.78 (0.67,0.92)	0.75 (0.65,0.85)	0.71 (0.61,0.83)	0.69 (0.56,0.86)
		Ethnicity: White	0.73 (0.65,0.82)	0.84 (0.75,0.93)	0.77 (0.71,0.84)	0.78 (0.73,0.83)	0.80 (0.74,0.87)	0.77 (0.69,0.87)
		Ethnicity: Black or Black British	0.48 (0.19,1.18)	0.89 (0.46,1.73)	0.85 (0.51,1.40)	0.64 (0.45,0.93)	0.70 (0.49,1.01)	0.42 (0.19,0.92)
		Ethnicity: Asian or Asian British	0.79 (0.42,1.46)	0.33 (0.14,0.77)	0.76 (0.49,1.19)	0.68 (0.50,0.94)	0.63 (0.44,0.89)	0.65 (0.36,1.19)
		Ethnicity: Other Ethnic Groups	1.03 (0.37,2.88)	0.16 (0.02,1.31)	0.37 (0.13,1.08)	0.64 (0.35,1.18)	0.45 (0.21,0.97)	0.60 (0.19,1.96)
		Ethnicity: Mixed	1.11 (0.32,3.84)	2.02 (0.73,5.62)	0.86 (0.33,2.25)	0.78 (0.36,1.69)	0.72 (0.31,1.72)	0.53 (0.11,2.68)
		Ethnicity: Unknown	0.72 (0.18,2.82)	0.91 (0.28,2.91)	1.17 (0.56,2.46)	0.86 (0.45,1.62)	0.62 (0.27,1.43)	0.72 (0.21,2.44)
		No prior history of COVID-19	0.73 (0.66,0.82)	0.82 (0.74,0.91)	0.77 (0.71,0.84)	0.78 (0.73,0.84)	0.80 (0.74,0.86)	0.77 (0.68,0.86)
		Prior history of COVID-19	0.69 (0.43,1.09)	0.88 (0.58,1.34)	0.84 (0.61,1.16)	0.63 (0.49,0.81)	0.66 (0.50,0.86)	0.84 (0.55,1.28)
		No prior history of event	0.74 (0.66,0.83)	0.80 (0.72,0.90)	0.76 (0.70,0.83)	0.77 (0.72,0.82)	0.76 (0.70,0.82)	0.71 (0.63,0.80)
		Prior history of event	0.68 (0.51,0.89)	0.92 (0.72,1.17)	0.81 (0.66,0.99)	0.72 (0.61,0.85)	0.74 (0.62,0.88)	0.82 (0.64,1.05)
		Sex: Female	0.72 (0.62,0.83)	0.81 (0.71,0.93)	0.76 (0.68,0.85)	0.75 (0.69,0.82)	0.74 (0.67,0.82)	0.71 (0.62,0.82)
		Sex: Male	0.76 (0.65,0.89)	0.83 (0.72,0.97)	0.78 (0.69,0.89)	0.81 (0.74,0.90)	0.88 (0.79,0.99)	0.87 (0.73,1.03)
**Doses 1&2: ChAdOx1**	Composite arterial	Age group: <40	0.56 (0.37,0.86)	0.89 (0.63,1.28)	0.63 (0.46,0.87)	0.65 (0.49,0.85)	0.54 (0.38,0.76)	0.68 (0.43,1.07)
		Age group: 40-59	0.70 (0.64,0.77)	0.82 (0.75,0.89)	0.80 (0.74,0.85)	0.81 (0.76,0.86)	0.81 (0.75,0.88)	0.83 (0.74,0.92)
		Age group: 60-79	0.73 (0.69,0.77)	0.83 (0.78,0.88)	0.79 (0.75,0.83)	0.72 (0.69,0.75)	0.68 (0.64,0.72)	0.66 (0.61,0.71)
		Age group: >=80	0.70 (0.64,0.77)	0.77 (0.70,0.84)	0.70 (0.65,0.75)	0.69 (0.64,0.73)	0.66 (0.62,0.72)	0.67 (0.60,0.74)
		Ethnicity: White	0.71 (0.68,0.75)	0.84 (0.80,0.87)	0.79 (0.77,0.82)	0.76 (0.74,0.79)	0.74 (0.71,0.77)	0.74 (0.70,0.78)
		Ethnicity: Black or Black British	0.75 (0.57,0.99)	0.67 (0.50,0.89)	0.72 (0.58,0.89)	0.72 (0.61,0.85)	0.65 (0.53,0.79)	0.58 (0.43,0.79)
		Ethnicity: Asian or Asian British	0.71 (0.60,0.83)	0.80 (0.69,0.94)	0.80 (0.71,0.91)	0.70 (0.63,0.78)	0.65 (0.57,0.73)	0.66 (0.55,0.78)
		Ethnicity: Other Ethnic Groups	0.56 (0.36,0.86)	0.81 (0.56,1.17)	0.64 (0.47,0.87)	0.63 (0.49,0.82)	0.73 (0.52,1.01)	0.77 (0.50,1.20)
		Ethnicity: Mixed	1.09 (0.68,1.77)	0.98 (0.60,1.59)	1.09 (0.76,1.58)	0.97 (0.70,1.36)	0.89 (0.58,1.36)	1.02 (0.57,1.83)
		Ethnicity: Unknown	0.90 (0.64,1.27)	0.85 (0.59,1.21)	0.84 (0.63,1.12)	0.75 (0.57,0.98)	0.72 (0.51,1.02)	0.69 (0.43,1.10)
		No prior history of COVID-19	0.72 (0.69,0.75)	0.83 (0.80,0.87)	0.79 (0.76,0.82)	0.76 (0.74,0.78)	0.73 (0.70,0.76)	0.73 (0.69,0.77)
		Prior history of COVID-19	0.71 (0.61,0.83)	0.77 (0.66,0.90)	0.80 (0.72,0.90)	0.68 (0.61,0.75)	0.68 (0.60,0.77)	0.68 (0.57,0.81)
		No prior history of event	0.68 (0.64,0.72)	0.82 (0.78,0.87)	0.83 (0.79,0.86)	0.74 (0.72,0.77)	0.68 (0.65,0.71)	0.63 (0.60,0.68)
		Prior history of event	0.74 (0.70,0.79)	0.81 (0.76,0.86)	0.71 (0.68,0.75)	0.72 (0.69,0.75)	0.71 (0.68,0.75)	0.76 (0.71,0.82)
		Sex: Female	0.70 (0.66,0.75)	0.82 (0.77,0.88)	0.77 (0.73,0.81)	0.75 (0.71,0.79)	0.73 (0.69,0.78)	0.75 (0.69,0.81)
		Sex: Male	0.74 (0.70,0.78)	0.85 (0.81,0.89)	0.82 (0.79,0.86)	0.79 (0.76,0.82)	0.77 (0.73,0.81)	0.77 (0.72,0.83)
	Composite venous	Age group: <40	0.60 (0.41,0.88)	0.89 (0.64,1.22)	0.59 (0.43,0.80)	0.49 (0.39,0.61)	0.48 (0.37,0.62)	0.44 (0.30,0.66)
		Age group: 40-59	0.71 (0.60,0.83)	0.86 (0.74,1.01)	0.85 (0.75,0.96)	0.70 (0.63,0.79)	0.73 (0.64,0.84)	0.71 (0.59,0.85)
		Age group: 60-79	0.69 (0.61,0.79)	0.75 (0.66,0.85)	0.68 (0.61,0.76)	0.61 (0.55,0.67)	0.52 (0.46,0.59)	0.55 (0.47,0.64)
		Age group: >=80	0.85 (0.68,1.06)	0.89 (0.72,1.11)	0.82 (0.68,0.99)	0.68 (0.58,0.80)	0.62 (0.52,0.74)	0.60 (0.47,0.77)
		Ethnicity: White	0.73 (0.67,0.80)	0.84 (0.77,0.92)	0.79 (0.73,0.84)	0.70 (0.66,0.75)	0.67 (0.61,0.72)	0.68 (0.61,0.76)
		Ethnicity: Black or Black British	0.60 (0.31,1.14)	0.71 (0.39,1.30)	0.89 (0.58,1.37)	0.83 (0.59,1.15)	0.75 (0.51,1.10)	0.45 (0.24,0.83)
		Ethnicity: Asian or Asian British	0.56 (0.30,1.04)	0.77 (0.44,1.34)	0.95 (0.64,1.40)	0.67 (0.48,0.95)	0.55 (0.36,0.86)	0.67 (0.36,1.25)
		Ethnicity: Other Ethnic Groups	0.26 (0.06,1.12)	1.20 (0.55,2.60)	0.85 (0.42,1.72)	0.79 (0.43,1.45)	0.72 (0.33,1.57)	0.81 (0.24,2.75)
		Ethnicity: Mixed	1.37 (0.59,3.21)	0.49 (0.14,1.69)	0.56 (0.24,1.30)	0.47 (0.22,1.02)	0.54 (0.23,1.31)	0.73 (0.22,2.48)
		Ethnicity: Unknown	0.73 (0.33,1.62)	1.10 (0.55,2.19)	0.94 (0.52,1.69)	0.95 (0.56,1.64)	0.74 (0.37,1.46)	0.83 (0.34,2.03)
		No prior history of COVID-19	0.71 (0.65,0.78)	0.82 (0.75,0.90)	0.80 (0.75,0.86)	0.70 (0.66,0.74)	0.65 (0.60,0.71)	0.67 (0.60,0.74)
		Prior history of COVID-19	0.90 (0.67,1.20)	1.08 (0.82,1.43)	0.71 (0.55,0.92)	0.81 (0.66,0.99)	0.85 (0.66,1.10)	0.84 (0.58,1.21)
		No prior history of event	0.73 (0.67,0.80)	0.83 (0.76,0.91)	0.78 (0.73,0.84)	0.70 (0.65,0.74)	0.65 (0.60,0.70)	0.64 (0.57,0.71)
		Prior history of event	0.65 (0.52,0.82)	0.87 (0.71,1.06)	0.79 (0.68,0.93)	0.66 (0.58,0.76)	0.63 (0.54,0.74)	0.72 (0.58,0.91)
		Sex: Female	0.71 (0.63,0.81)	0.80 (0.71,0.90)	0.78 (0.71,0.86)	0.68 (0.62,0.74)	0.64 (0.58,0.71)	0.65 (0.57,0.75)
		Sex: Male	0.74 (0.65,0.83)	0.88 (0.79,0.99)	0.81 (0.74,0.89)	0.75 (0.69,0.81)	0.72 (0.65,0.81)	0.75 (0.65,0.87)

Supplementary Table 28: Subgroup-specific maximally-adjusted hazard ratios and 95% CIs for composite arterial and composite venous events by time since booster vaccination with BNT-162b2 or mRNA-1273for those on a primary course of ChAdOx1.

Vaccination course	Event	Stratum	Week 1	Week 2	Weeks 3-4	Weeks 5-12	Weeks 13-24
				Weeks 1-4			Weeks 5-24
**Doses 1&2: ChAdOx1; Booster: BNT-162b2**	Composite arterial	Age group: <40	0.88 (0.50, 1.57)	0.52 (0.26, 1.07)	0.53 (0.31, 0.90)	0.50 (0.31, 0.80)	1.01 (0.30, 3.36)
		Age group: 40-59	0.70 (0.62, 0.79)	0.79 (0.70, 0.89)	0.83 (0.75, 0.91)	0.82 (0.75, 0.90)	0.85 (0.64, 1.12)
		Age group: 60-79	0.79 (0.74, 0.84)	0.81 (0.76, 0.86)	0.80 (0.76, 0.84)	0.73 (0.70, 0.77)	0.65 (0.59, 0.72)
		Age group: >=80	0.78 (0.70, 0.86)	0.79 (0.71, 0.88)	0.80 (0.73, 0.86)	0.75 (0.70, 0.80)	0.70 (0.62, 0.80)
		Ethnicity: White	0.78 (0.74, 0.83)	0.82 (0.77, 0.86)	0.82 (0.79, 0.85)	0.78 (0.75, 0.80)	0.69 (0.64, 0.75)
		Ethnicity: Black or Black British	0.96 (0.65, 1.41)	0.90 (0.60, 1.35)	0.91 (0.67, 1.24)	0.82 (0.63, 1.07)	1.12 (0.61, 2.05)
		Ethnicity: Asian or Asian British	0.78 (0.63, 0.97)	0.89 (0.72, 1.09)	0.87 (0.74, 1.02)	0.75 (0.65, 0.85)	0.80 (0.58, 1.11)
		Ethnicity: Other Ethnic Groups	0.81 (0.50, 1.32)	0.80 (0.49, 1.30)	0.78 (0.53, 1.14)	0.67 (0.49, 0.92)	0.51 (0.21, 1.26)
		Ethnicity: Mixed	0.63 (0.29, 1.38)	0.46 (0.18, 1.15)	1.00 (0.61, 1.63)	0.70 (0.43, 1.14)	0.56 (0.16, 1.99)
		Ethnicity: Unknown	0.57 (0.35, 0.94)	0.50 (0.29, 0.87)	0.69 (0.48, 0.99)	0.70 (0.51, 0.97)	0.72 (0.33, 1.57)
		No prior history of COVID-19	0.78 (0.74,0.82)	0.82 (0.78,0.86)	0.83 (0.79,0.86)	0.78 (0.75,0.81)	0.71 (0.65,0.76)
		Prior history of COVID-19	0.90 (0.75,1.08)	0.81 (0.67,0.99)	0.83 (0.72,0.96)	0.76 (0.67,0.86)	0.72 (0.54,0.95)
		No prior history of event	0.70 (0.66,0.75)	0.78 (0.74,0.84)	0.76 (0.72,0.80)	0.71 (0.68,0.74)	0.63 (0.57,0.69)
		Prior history of event	0.89 (0.82,0.95)	0.84 (0.78,0.91)	0.89 (0.84,0.95)	0.85 (0.81,0.89)	0.80 (0.72,0.90)
		Sex: Female	0.83 (0.77,0.90)	0.80 (0.74,0.87)	0.85 (0.80,0.90)	0.80 (0.76,0.85)	0.70 (0.62,0.79)
		Sex: Male	0.77 (0.72,0.82)	0.85 (0.80,0.90)	0.84 (0.80,0.88)	0.81 (0.77,0.84)	0.76 (0.69,0.84)
	Composite venous	Age group: <40	0.81 (0.46, 1.41)	1.15 (0.71, 1.88)	0.96 (0.65, 1.41)	0.68 (0.47, 0.99)	1.24 (0.48, 3.17)
		Age group: 40-59	0.71 (0.57, 0.89)	0.91 (0.75, 1.12)	0.75 (0.63, 0.89)	0.79 (0.68, 0.92)	0.57 (0.33, 0.97)
		Age group: 60-79	0.62 (0.53, 0.71)	0.62 (0.54, 0.71)	0.64 (0.58, 0.72)	0.53 (0.48, 0.59)	0.58 (0.47, 0.72)
		Age group: >=80	0.55 (0.42, 0.73)	0.59 (0.45, 0.77)	0.56 (0.46, 0.69)	0.53 (0.45, 0.62)	0.46 (0.33, 0.65)
		Ethnicity: White	0.68 (0.61, 0.76)	0.74 (0.67, 0.83)	0.72 (0.67, 0.79)	0.65 (0.60, 0.70)	0.64 (0.55, 0.76)
		Ethnicity: Black or Black British		0.69 (0.44, 1.09)			0.49 (0.29, 0.82)
		Ethnicity: Asian or Asian British	0.37 (0.14, 1.00)	1.15 (0.62, 2.16)	0.72 (0.40, 1.28)	0.75 (0.48, 1.20)	0.51 (0.11, 2.45)
		Ethnicity: Other Ethnic Groups		0.78 (0.33, 1.82)			0.89 (0.36, 2.20)
		Ethnicity: Mixed	1.14 (0.28, 4.62)	1.35 (0.35, 5.11)	1.75 (0.80, 3.83)	0.32 (0.12, 0.85)	0.84 (0.14, 5.11)
		Ethnicity:	0.22 (0.05, 0.92)	0.43 (0.15, 1.19)	0.42 (0.20, 0.88)	0.40 (0.21, 0.74)	0.81 (0.26, 2.49)
		No prior history of COVID-19	0.66 (0.59,0.74)	0.72 (0.65,0.80)	0.71 (0.66,0.77)	0.64 (0.59,0.69)	0.62 (0.52,0.73)
		Prior history of COVID-19	0.84 (0.57,1.25)	1.03 (0.72,1.48)	0.87 (0.64,1.17)	0.61 (0.47,0.81)	0.66 (0.35,1.23)
		No prior history of event	0.67 (0.60,0.75)	0.72 (0.64,0.80)	0.72 (0.66,0.79)	0.62 (0.57,0.67)	0.59 (0.50,0.70)
		Prior history of event	0.63 (0.47,0.85)	0.86 (0.66,1.12)	0.68 (0.55,0.85)	0.70 (0.58,0.83)	0.74 (0.49,1.11)
		Sex: Female	0.74 (0.64,0.85)	0.77 (0.67,0.89)	0.76 (0.68,0.84)	0.66 (0.60,0.73)	0.54 (0.43,0.67)
		Sex: Male	0.62 (0.52,0.72)	0.73 (0.63,0.85)	0.71 (0.64,0.80)	0.66 (0.59,0.73)	0.81 (0.65,1.01)
**Doses 1&2: ChAdOx1; Booster: mRNA-1273**	Composite arterial	Age group: <40		0.72 (0.40, 1.27)			0.46 (0.19, 1.10)
		Age group: 40-59		0.84 (0.76, 0.94)			0.73 (0.63,0.85)
		Age group: 60-79		0.72 (0.66, 0.79)			0.60 (0.54, 0.67)
		Age group: >=80		0.80 (0.65, 0.99)			0.88 (0.70, 1.10)
		Ethnicity: White		0.76 (0.71, 0.81)			0.66 (0.61, 0.72)
		Ethnicity: Black or Black British		0.72 (0.42, 1.21)			0.51 (0.24, 1.07)
		Ethnicity: Asian or Asian British		0.74 (0.57, 0.96)			0.81 (0.59, 1.11)
		Ethnicity: Other Ethnic Groups		1.07 (0.64, 1.79)			0.75 (0.37, 1.54)
		Ethnicity: Mixed		0.57 (0.24, 1.34)			0.17 (0.04, 0.78)
		Ethnicity: Unknown		0.81 (0.54, 1.23)			0.44 (0.24, 0.81)
		No prior history of COVID-19		0.76 (0.71, 0.81)			0.67 (0.61, 0.72)
		Prior history of COVID-19		0.86 (0.68, 1.10)			0.62 (0.45, 0.86)
		No prior history of event		0.71 (0.66, 0.77)			0.61 (0.56, 0.67)
		Prior history of event		0.88 (0.79, 0.98)			0.74 (0.64, 0.84)
		Sex: Female		0.76 (0.68, 0.84)			0.65 (0.56, 0.74)
		Sex: Male		0.78 (0.72, 0.84)			0.68 (0.62, 0.75)
	Composite venous	Age group: <40		0.67 (0.41, 1.11)			0.69 (0.36, 1.33)
		Age group: 40-59		0.63 (0.52, 0.77)			0.64 (0.50, 0.82)
		Age group: 60-79		0.51 (0.42, 0.61)			0.40 (0.32, 0.50)
		Age group: >=80		0.49 (0.27, 0.88)			0.81 (0.46, 1.42)
		Ethnicity: White		0.58 (0.51, 0.66)			0.54 (0.46, 0.63)
		Ethnicity: Black or Black British		0.61 (0.24, 1.58)			0.52 (0.15, 1.80)
		Ethnicity: Asian or Asian British		0.31 (0.10, 1.01)			0.37 (0.09, 1.62)
		Ethnicity: Other Ethnic Groups		0.97 (0.23, 4.03)			2.05 (0.44, 9.59)
		Ethnicity: Mixed		1.78 (0.54, 5.83)			1.16 (0.19, 7.08)
		Ethnicity: Unknown		0.35 (0.13, 0.94)			0.26 (0.08, 0.78)
		No prior history of COVID-19		0.58 (0.51,0.65)			0.56 (0.48,0.66)
		Prior history of COVID-19		0.75 (0.48,1.18)			0.45 (0.22,0.91)
		No prior history of event		0.55 (0.48,0.62)			0.51 (0.43,0.61)
		Prior history of event		0.83 (0.61,1.15)			0.75 (0.50,1.12)
		Sex: Female		0.54 (0.45,0.65)			0.55 (0.44,0.69)
		Sex: Male		0.64 (0.55,0.76)			0.58 (0.47,0.72)

Supplementary Table 29: Subgroup-specific maximally-adjusted hazard ratios and 95% CIs for composite arterial and composite venous events by time since booster with BNT-162b2 or mRNA-1273 for those on a primary course of BNT-162b2[10].

Vaccination course	Event	Stratum	Week 1	Week 2	Weeks 3-4	Weeks 5-12	Weeks 13-24
				Weeks 1-4		Weeks 5-24	
**Doses 1&2: BNT-162b2; Booster: BNT-162b2**	Composite arterial	Age group: <40	0.71 (0.40, 1.29)	1.02 (0.60, 1.75)	0.71 (0.45, 1.12)	1.17 (0.79, 1.73)	1.04 (0.44, 2.45)
		Age group: 40-59	0.67 (0.56, 0.80)	0.78 (0.66, 0.92)	0.85 (0.75, 0.96)	0.79 (0.71, 0.87)	0.70 (0.56, 0.88)
		Age group: 60-79	0.67 (0.62, 0.73)	0.79 (0.73, 0.86)	0.77 (0.72, 0.82)	0.73 (0.69, 0.78)	0.68 (0.61, 0.75)
		Age group: >=80	0.65 (0.60, 0.70)	0.69 (0.64, 0.75)	0.71 (0.67, 0.76)	0.68 (0.64, 0.72)	0.63 (0.58, 0.68)
		Ethnicity: White	0.68 (0.64, 0.72)	0.79 (0.74, 0.83)	0.79 (0.76, 0.83)	0.77 (0.74, 0.80)	0.72 (0.68, 0.76)
		Ethnicity: Black or Black British	0.96 (0.63, 1.48)	0.86 (0.55, 1.34)	0.73 (0.51, 1.05)	0.75 (0.58, 0.98)	1.03 (0.64, 1.66)
		Ethnicity: Asian or Asian British	0.75 (0.59, 0.96)	0.72 (0.56, 0.93)	0.83 (0.69, 1.00)	0.88 (0.76, 1.01)	0.71 (0.54, 0.92)
		Ethnicity: Other Ethnic Groups	0.75 (0.42, 1.33)	0.61 (0.33, 1.15)	0.77 (0.49, 1.20)	0.90 (0.64, 1.25)	0.85 (0.45, 1.61)
		Ethnicity: Mixed	0.50 (0.15, 1.70)	0.60 (0.22, 1.66)	1.36 (0.76, 2.44)	1.00 (0.57, 1.74)	2.04 (0.90, 4.64)
		Ethnicity: Unknown	0.59 (0.32, 1.09)	0.65 (0.36, 1.17)	0.81 (0.53, 1.23)	0.59 (0.41, 0.84)	0.46 (0.24, 0.86)
		No prior history of COVID-19	0.69 (0.65, 0.73)	0.78 (0.74, 0.83)	0.79 (0.76, 0.83)	0.78 (0.75, 0.81)	0.73 (0.69, 0.77)
		Prior history of COVID-19	0.83 (0.64, 1.08)	0.82 (0.63, 1.08)	1.02 (0.84, 1.23)	0.87 (0.74, 1.03)	0.80 (0.59, 1.07)
		No prior history of event	0.62 (0.58, 0.67)	0.72 (0.68, 0.78)	0.76 (0.72, 0.80)	0.73 (0.69, 0.76)	0.65 (0.60, 0.70)
		Prior history of event	0.77 (0.71, 0.83)	0.85 (0.78, 0.91)	0.84 (0.79, 0.89)	0.82 (0.78, 0.87)	0.83 (0.76, 0.91)
		Sex: Female	0.67 (0.62, 0.73)	0.76 (0.70, 0.82)	0.81 (0.76, 0.86)	0.76 (0.72, 0.80)	0.71 (0.65, 0.78)
		Sex: Male	0.72 (0.67, 0.77)	0.83 (0.77, 0.89)	0.82 (0.78, 0.87)	0.84 (0.80, 0.88)	0.79 (0.73, 0.86)
	Composite venous	Age group: <40	0.61 (0.35, 1.09)	1.01 (0.64, 1.59)	0.90 (0.62, 1.30)	0.99 (0.71, 1.37)	0.74 (0.32, 1.75)
		Age group: 40-59	0.85 (0.63, 1.14)	0.65 (0.47, 0.91)	0.72 (0.57, 0.92)	0.66 (0.54, 0.79)	0.60 (0.42, 0.86)
		Age group: 60-79	0.61 (0.51, 0.73)	0.56 (0.47, 0.68)	0.54 (0.47, 0.62)	0.46 (0.40, 0.52)	0.48 (0.38, 0.59)
		Age group: >=80	0.44 (0.36, 0.55)	0.54 (0.44, 0.65)	0.46 (0.40, 0.54)	0.42 (0.37, 0.48)	0.35 (0.29, 0.43)
		Ethnicity: White	0.63 (0.56, 0.72)	0.65 (0.58, 0.73)	0.65 (0.59, 0.71)	0.60 (0.56, 0.65)	0.55 (0.49, 0.63)
		Ethnicity: Black or Black British	0.45 (0.14, 1.44)	0.49 (0.15, 1.60)	0.57 (0.26, 1.24)	0.81 (0.47, 1.40)	0.41 (0.12, 1.39)
		Ethnicity: Asian or Asian British	0.45 (0.16, 1.22)	0.44 (0.16, 1.22)	0.60 (0.31, 1.16)	0.55 (0.34, 0.89)	0.52 (0.20, 1.35)
		Ethnicity: Other Ethnic Groups		0.48 (0.16, 1.45)		0.94 (0.40, 2.22)	
		Ethnicity: Mixed	1.62 (0.30, 8.84)	1.81 (0.34, 9.72)	1.42 (0.34, 5.85)	0.84 (0.24, 2.94)	0.96 (0.09, 10.43)
		Ethnicity: Unknown	0.46 (0.14, 1.56)	1.13 (0.45, 2.79)	0.17 (0.04, 0.72)	0.44 (0.21, 0.94)	0.21 (0.04, 1.22)
		No prior history of COVID-19	0.62 (0.55, 0.70)	0.66 (0.58, 0.74)	0.64 (0.58, 0.70)	0.60 (0.56, 0.65)	0.54 (0.48, 0.62)
		Prior history of COVID-19	0.81 (0.48, 1.39)	0.65 (0.36, 1.17)	0.67 (0.43, 1.05)	0.70 (0.51, 0.97)	0.63 (0.33, 1.19)
		No prior history of event	0.60 (0.53, 0.69)	0.65 (0.57, 0.74)	0.62 (0.57, 0.69)	0.59 (0.55, 0.64)	0.53 (0.46, 0.60)
		Prior history of event	0.72 (0.54, 0.96)	0.66 (0.49, 0.89)	0.70 (0.56, 0.88)	0.64 (0.53, 0.77)	0.66 (0.47, 0.92)
		Sex: Female	0.58 (0.50, 0.69)	0.60 (0.51, 0.70)	0.60 (0.53, 0.67)	0.58 (0.53, 0.64)	0.57 (0.48, 0.68)
		Sex: Male	0.68 (0.57, 0.81)	0.74 (0.62, 0.88)	0.70 (0.61, 0.80)	0.64 (0.58, 0.72)	0.53 (0.44, 0.65)
**Doses 1&2: BNT-162b2; Booster: mRNA-1273**	Composite arterial	Age group: <40		0.38 (0.21, 0.69)		0.19 (0.07, 0.53)	
		Age group: 40-59	0.63 (0.42, 0.94)	0.96 (0.68, 1.34)	0.83 (0.63,1.09)	0.79 (0.61, 1.01)	13.78 (1.83,103.78)
		Age group: 60-79		0.70 (0.61, 0.82)		0.65 (0.55, 0.78)	
		Age group: >=80	0.81 (0.54, 1.22)	0.95 (0.65, 1.40)	0.68 (0.49, 0.96)	0.67 (0.51, 0.87)	0.94 (0.23, 3.85)
		Ethnicity: White	0.68 (0.55, 0.84)	0.93 (0.77, 1.12)	0.88 (0.76, 1.02)	0.85 (0.75, 0.96)	1.19 (0.37, 3.80)
		Ethnicity: Black or Black British		1.07 (0.50, 2.28)		1.33 (0.57, 3.10)	
		Ethnicity: Asian or Asian British		0.83 (0.53, 1.32)		0.70 (0.38, 1.30)	
		Ethnicity: Other Ethnic Groups		1.31 (0.53, 3.25)		1.68 (0.57, 4.90)	
		Ethnicity: Mixed		2.13 (0.74, 6.14)		1.28 (0.26, 6.24)	
		Ethnicity: Unknown		0.54 (0.19, 1.56)		0.11 (0.01, 0.96)	
		No prior history of COVID-19	0.68 (0.55,0.83)	0.96 (0.80,1.15)	0.88 (0.76,1.01)	0.85 (0.75,0.96)	1.12 (0.35,3.59)
		Prior history of COVID-19		1.21 (0.79, 1.86)		0.96 (0.53, 1.75)	
		No prior history of event	0.59 (0.45, 0.78)	0.86 (0.68, 1.09)	0.82 (0.69, 0.98)	0.72 (0.62, 0.85)	1.21 (0.38, 3.91)
		Prior history of event		0.83 (0.71, 0.96)		0.81 (0.68, 0.97)	
		Sex: Female	0.62 (0.44,0.87)	0.58 (0.41,0.83)	0.80 (0.63,1.00)	0.67 (0.54,0.82)	1.30 (0.30,5.51)
		Sex: Male	0.69 (0.54,0.89)	1.20 (0.99,1.47)	0.93 (0.78,1.10)	0.97 (0.83,1.13)	0.80 (0.11,5.80)
	Composite venous	Age group: <40		0.94 (0.61, 1.45)		0.85 (0.37, 1.94)	
		Age group: 40-59		0.60 (0.39, 0.93)		0.57 (0.35, 0.93)	
		Age group: 60-79		0.39 (0.28, 0.56)		0.44 (0.30, 0.65)	
		Age group: >=80		0.52 (0.30, 0.90)		0.31 (0.15, 0.65)	
		Ethnicity: White		0.60 (0.48, 0.75)		0.58 (0.45, 0.76)	
		Ethnicity: Black or Black British		0.32 (0.04, 2.48)		0.77 (0.17, 3.42)	
		Ethnicity: Asian or Asian British		0.83 (0.25, 2.78)		0.40 (0.06, 2.68)	
		Ethnicity: Other Ethnic Groups		0.92 (0.18, 4.69)		0.58 (0.05, 6.45)	
		Ethnicity: Mixed		0.61 (0.49, 0.75)		0.57 (0.44, 0.73)	
		Ethnicity: Unknown		0.39 (0.12, 1.28)		0.21 (0.03, 1.47)	
		No prior history of COVID-19		0.61 (0.49, 0.76)		0.54 (0.42, 0.71)	
		Prior history of COVID-19		0.54 (0.31, 0.95)		0.54 (0.27, 1.08)	
		No prior history of event		0.62 (0.47, 0.81)		0.67 (0.48, 0.93)	
		Prior history of event		0.61 (0.44, 0.83)		0.48 (0.32, 0.71)	
		Sex: Female		0.94 (0.61, 1.45)		0.85 (0.37, 1.94)	
		Sex: Male		0.60 (0.39, 0.93)		0.57 (0.35, 0.93)	

[10] Stratified analyses for primary course mRNA-1273 are not presented, nor are booster with mRNA-1273 only, due to the small number of individuals who received mRNA-1273.

Supplementary Table 30: Associations of COVID-19 vaccination brand and dose with myocarditis and pericarditis until the CDC's Announcement on 17 May 2021.

Event	Vaccination course	Week 1	Week 2	Weeks 3-4	Weeks 5-12	Weeks 13-24
					Week 5-24	
**Myocarditis**	**Dose 1: ChAdOx1**	1.42 (0.83, 2.45)	0.53 (0.23, 1.21)	1.13 (0.72, 1.76)	0.77 (0.52, 1.12)	0.69 (0.32, 1.46)
	**Dose 1: BNT-162b2**	1.85 (0.92, 3.72)	1.20 (0.52, 2.74)	0.87 (0.43, 1.76)	0.74 (0.47, 1.17)	0.74 (0.38, 1.46)
	**Doses 1&2: ChAdOx1**	0.55 (0.14, 2.20)	0.64 (0.15, 2.73)	0.76 (0.22, 2.55)	1.15 (0.23, 5.77)	
	**Doses 1&2: BNT-162b2**	0.95 (0.27, 3.38)	1.41 (0.42, 4.76)	0.60 (0.17, 2.08)	1.21 (0.41, 3.54)	
**Pericarditis**	**Dose 1: ChAdOx1**	0.88 (0.44, 1.74)	1.57 (0.92, 2.68)	1.21 (0.76, 1.93)	1.08 (0.76, 1.54)	0.65 (0.31, 1.34)
	**Dose 1: BNT-162b2**	0.43 (0.11, 1.78)	0.95 (0.34, 2.64)	1.19 (0.62, 2.30)	1.15 (0.75, 1.76)	1.89 (1.13, 3.16)
	**Doses 1&2: ChAdOx1**	1.18 (0.51, 2.70)	1.19 (0.48, 2.93)	0.57 (0.22, 1.47)	0.41 (0.09, 1.82)	
	**Doses 1&2: BNT-162b2**	0.31 (0.07, 1.33)	1.23 (0.48, 3.12)	1.35 (0.62, 2.97)	1.34 (0.63, 2.89)	

Supplementary Figures

Supplementary Figure 1: Consort Flow Diagram for Population Inclusion/Exclusion Criteria[11].

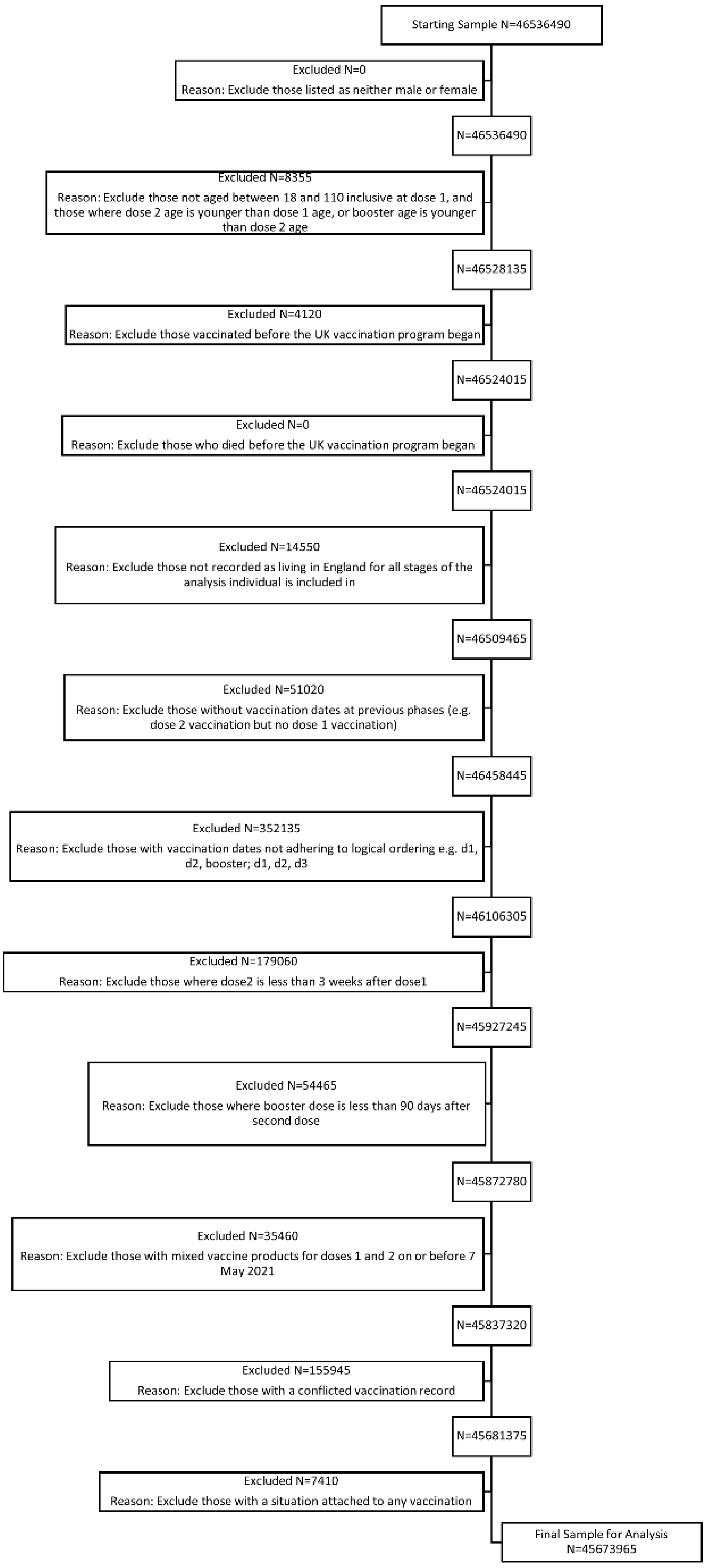

[11] Counts rounded to the nearest 5. Number excluded at each stage calculated from the rounded counts. 14,550 excluded due to having a latest non-NULL LSOA recorded as non-English, although latest LSOAs assigned as NULL (due to having no latest non-NULL LSOA on record, or due to having conflicting latest LSOAs) were included.

Supplementary Figure 2: Subgroup-specific maximally-adjusted hazard ratios for a composite arterial event by time since dose 1 vaccination.

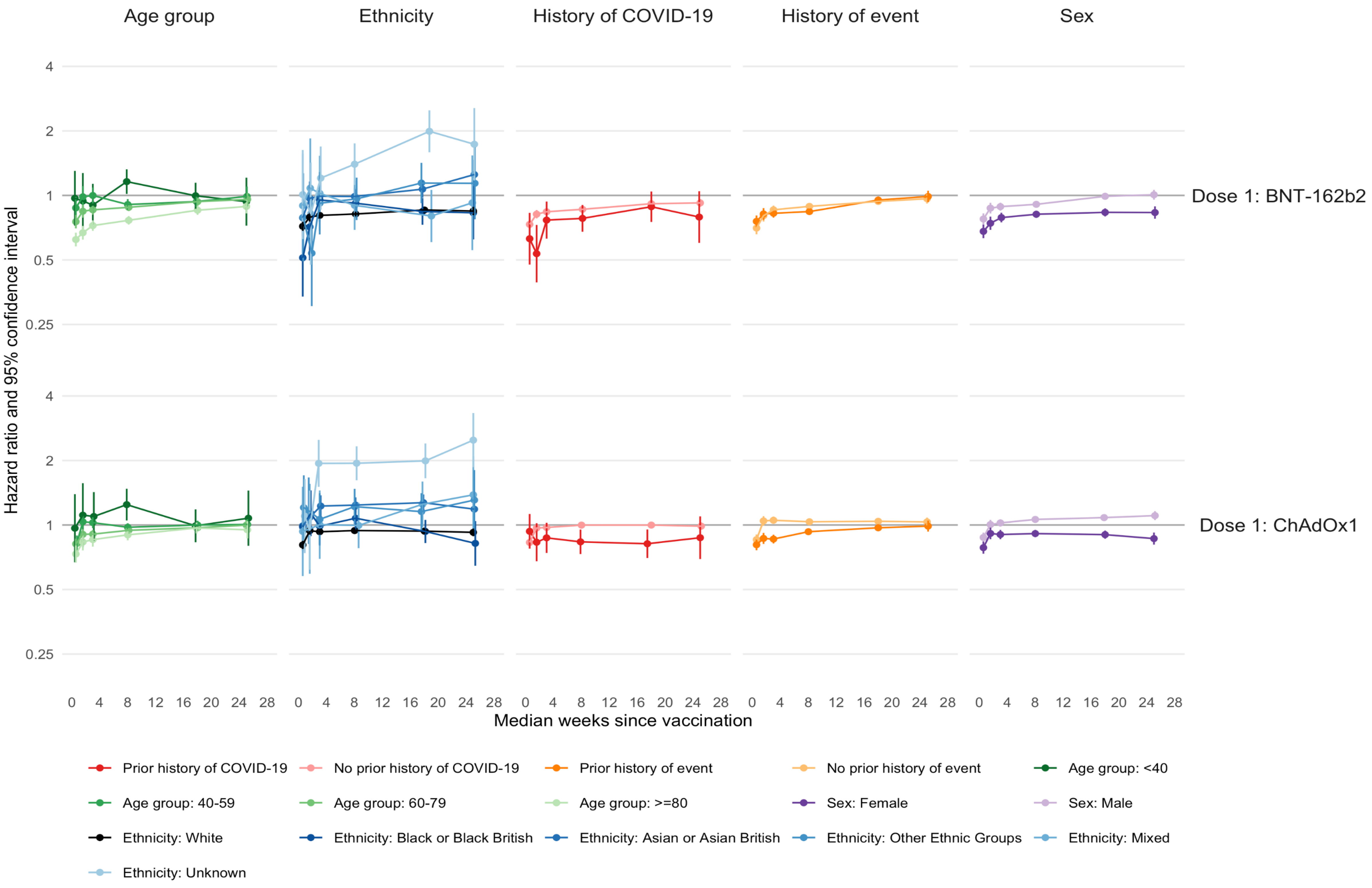

The number of people eligible for first dose analyses was 45,673,965.

The number of people who received a first dose of ChAdOx1, BNT-162b2 were 19,317,985 and 16,846,995 respectively.

The numerical values of hazard ratios and 95% CIs are displayed in Supplementary Tables 26.

Supplementary Figure 3: Subgroup-specific maximally-adjusted hazard ratios for a composite venous event by time since dose 1 vaccination.

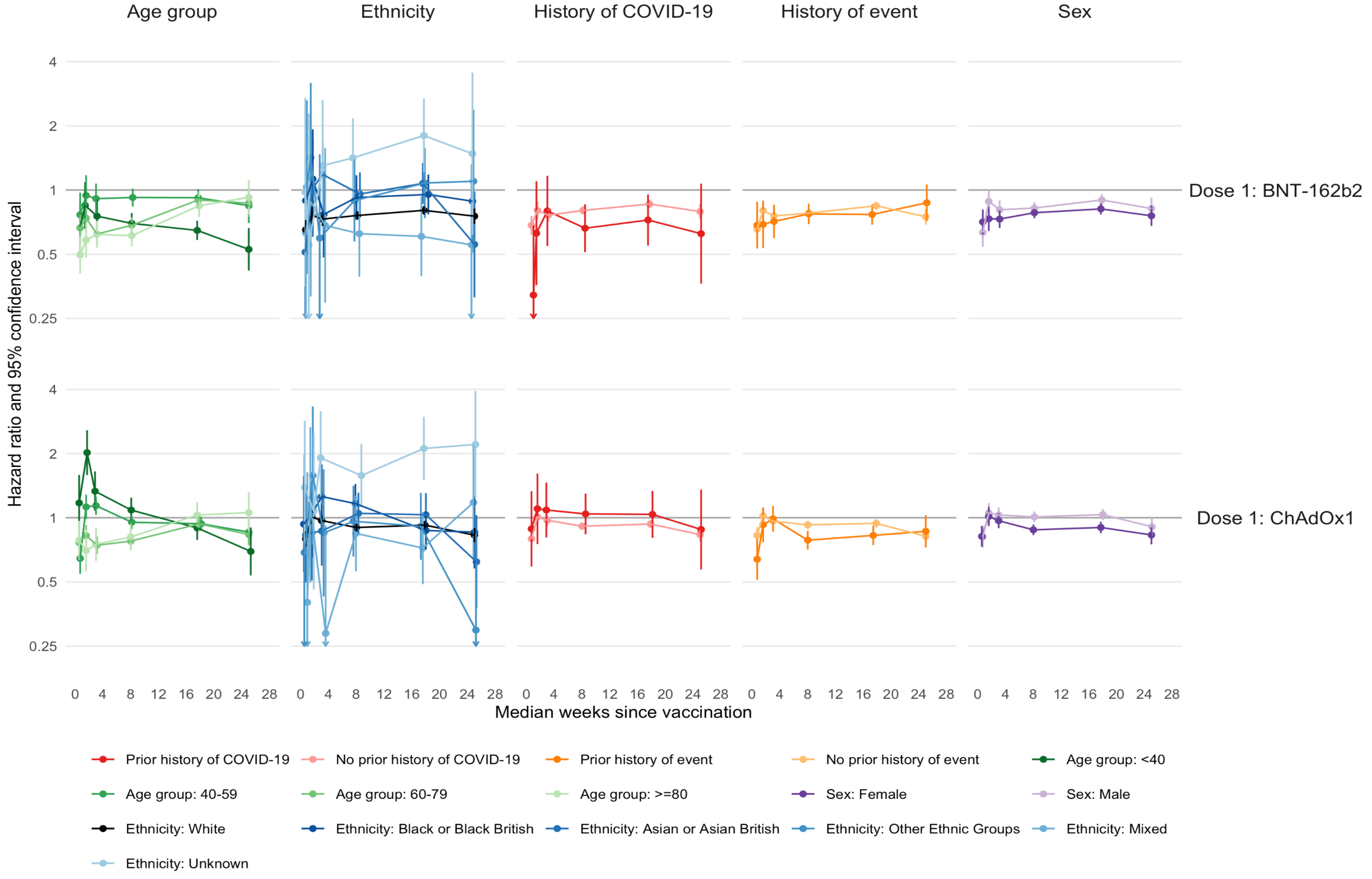

The number of people eligible for first dose analyses was 45,673,965.

The number of people who received a first dose of ChAdOx1, BNT-162b2 were 19,317,985 and 16,846,995 respectively.

The numerical values of hazard ratios and 95% CIs are displayed in Supplementary Tables 26.

Supplementary Figure 4: Subgroup-specific maximally-adjusted hazard ratios for a composite arterial event by time since second dose vaccination.

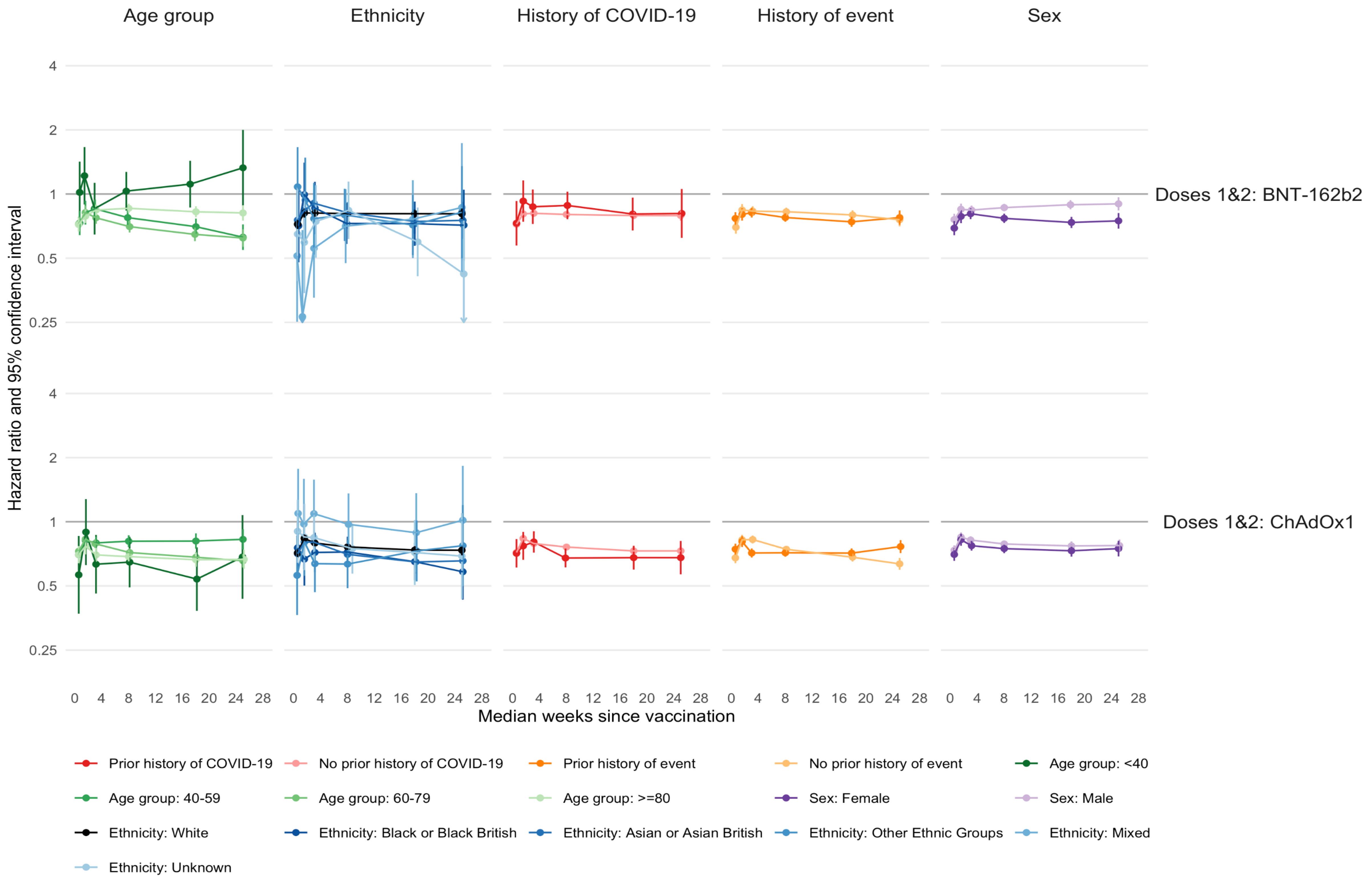

The number of people eligible for second dose analyses was 37,249,850.

The number of people who received a second dose of ChAdOx1, BNT-162b2 were 18,920,225 and 15,961,330 respectively.

The numerical values of hazard ratios and 95% CIs are displayed in Supplementary Tables 27

Supplementary Figure 5: Subgroup-specific maximally-adjusted hazard ratios for a composite venous event by time since second dose vaccination.

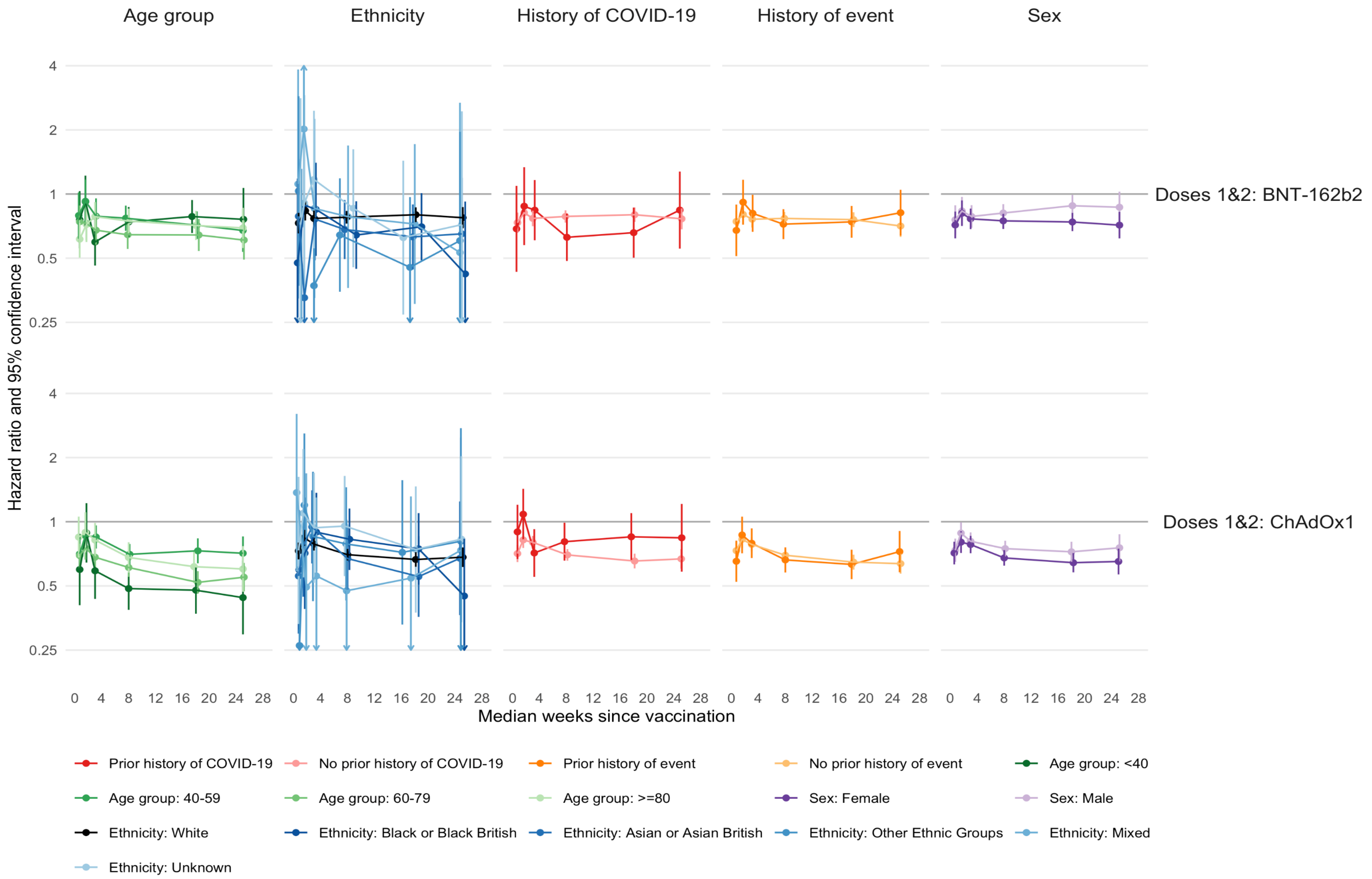

The number of people eligible for second dose analyses was 37,249,850.

The number of people who received a second dose of ChAdOx1, BNT-162b2 were 18,920,225 and 15,961,330 respectively.

The numerical values of hazard ratios and 95% CIs are displayed in Supplementary Tables 27.

Supplementary Figure 6: Subgroup-specific maximally-adjusted hazard ratios for a composite arterial event by time since booster vaccination[12].

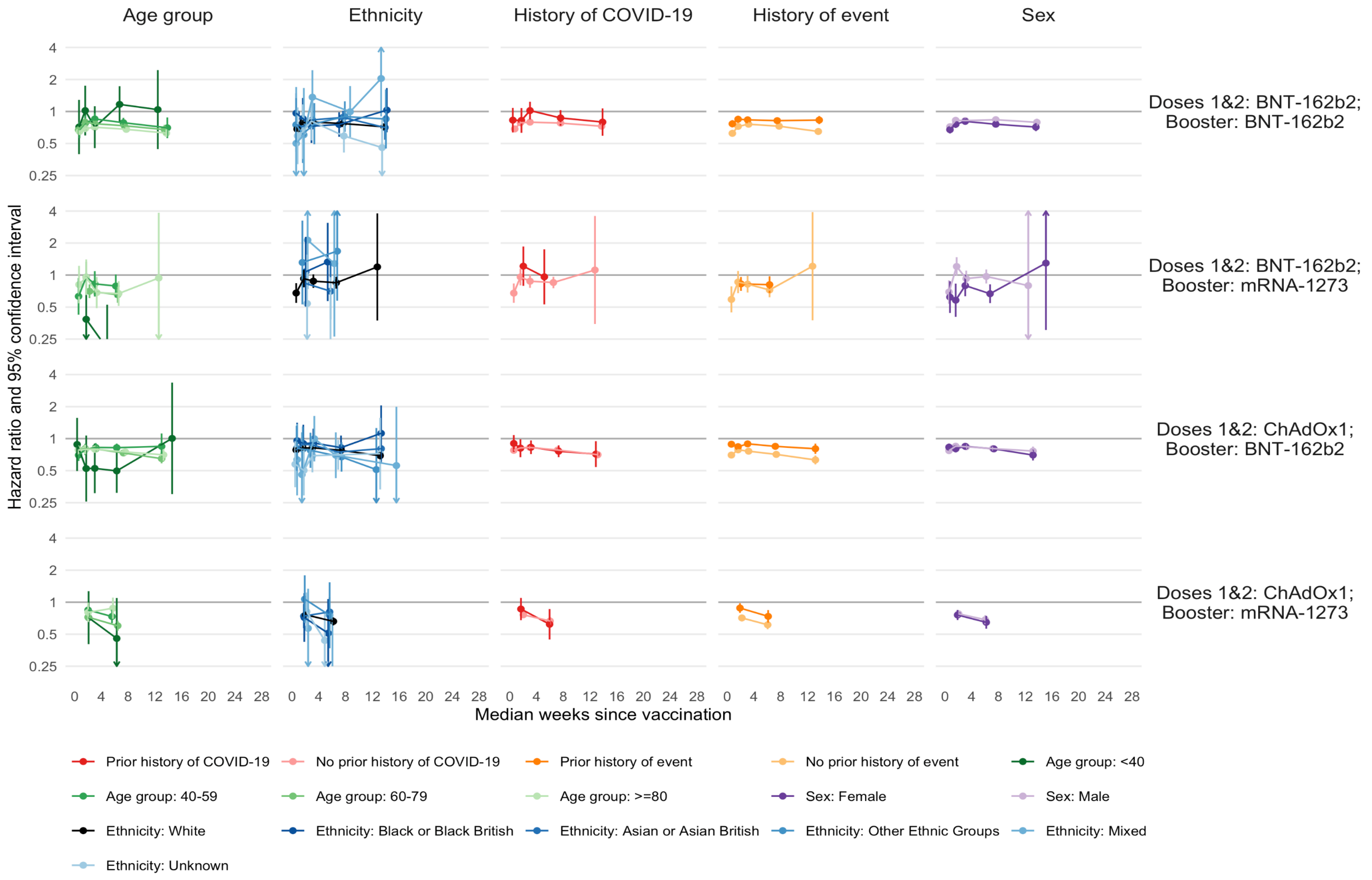

The number of people eligible for booster dose analyses was 35,853,120.

The number of people who received a booster dose of BNT-162b2 and mRNA-1273 following a primary course of ChAdOx1 were 11,964,635and 4,153,760 respectively; a booster dose of BNT-162b2 and mRNA-1273 following a primary course of BNT-162b2 were 9,821,835 and 1,914,925 respectively.

The numerical values of hazard ratios and 95% CIs are displayed in Supplementary Tables 28, 29.

[12] The aHR for weeks 12-24 for Age group: 40-59, Doses 1&2: BNT-162b2/mRNA-1273; Booster: mRNA-1273 could not be estimated due to small counts.

Supplementary Figure 7: Subgroup-specific maximally-adjusted hazard ratios for a composite venous event by time since booster vaccination.

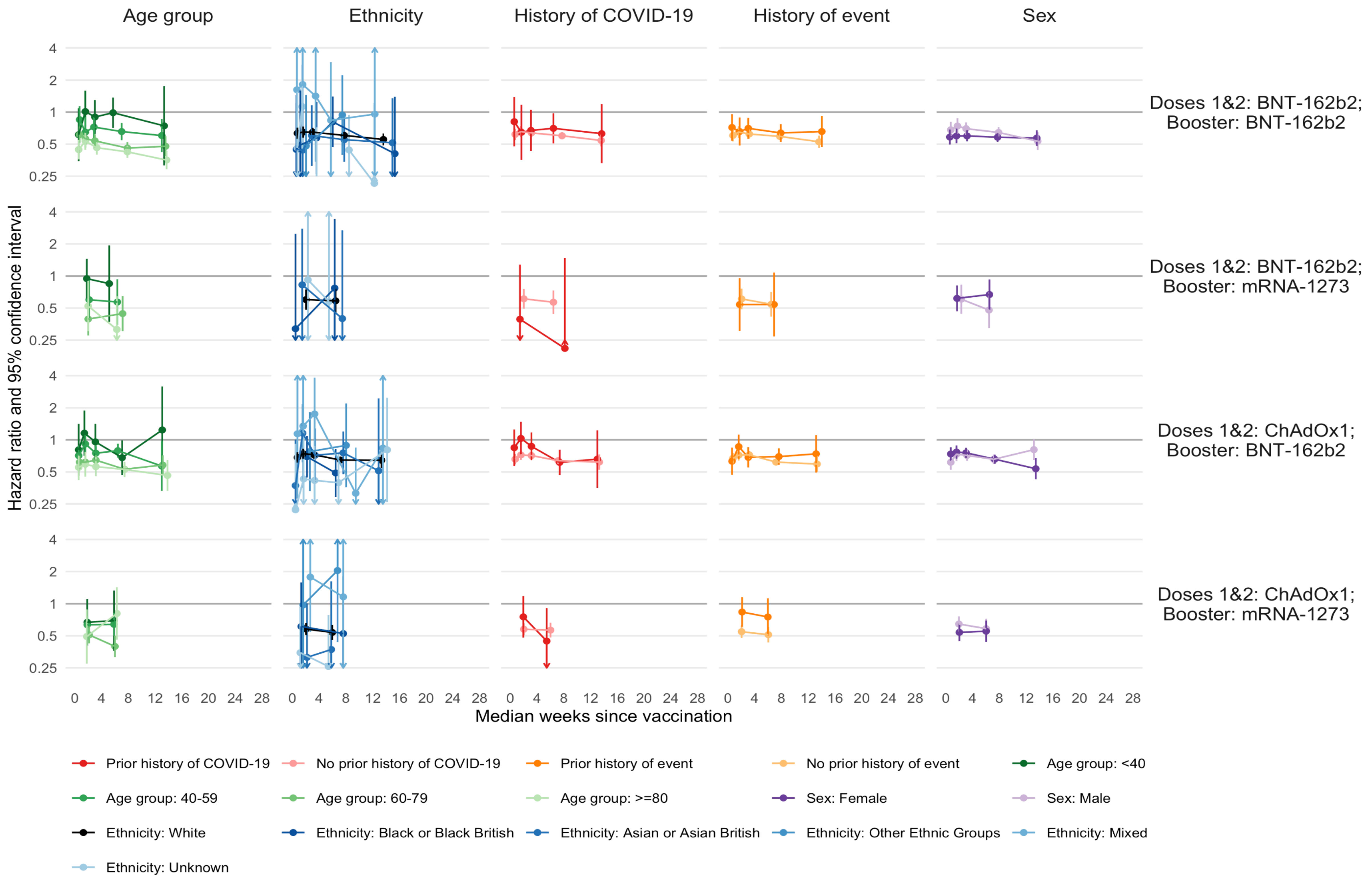

The number of people eligible booster dose analyses was 35,853,120.

The number of people who received a booster dose of BNT-162b2 and mRNA-1273 following a primary course of ChAdOx1 were 11,964,635and 4,153,760 respectively; a booster dose of BNT-162b2 and mRNA-1273 following a primary course of BNT-162b2 were 9,821,835 and 1,914,925 respectively.

The numerical values of hazard ratios and 95% CIs are displayed in Supplementary Tables 28, 29.

Supplementary Figure 8: Associations of COVID-19 vaccination brand and dose with myocarditis and pericarditis until the CDC's Announcement on 17 May 2021.

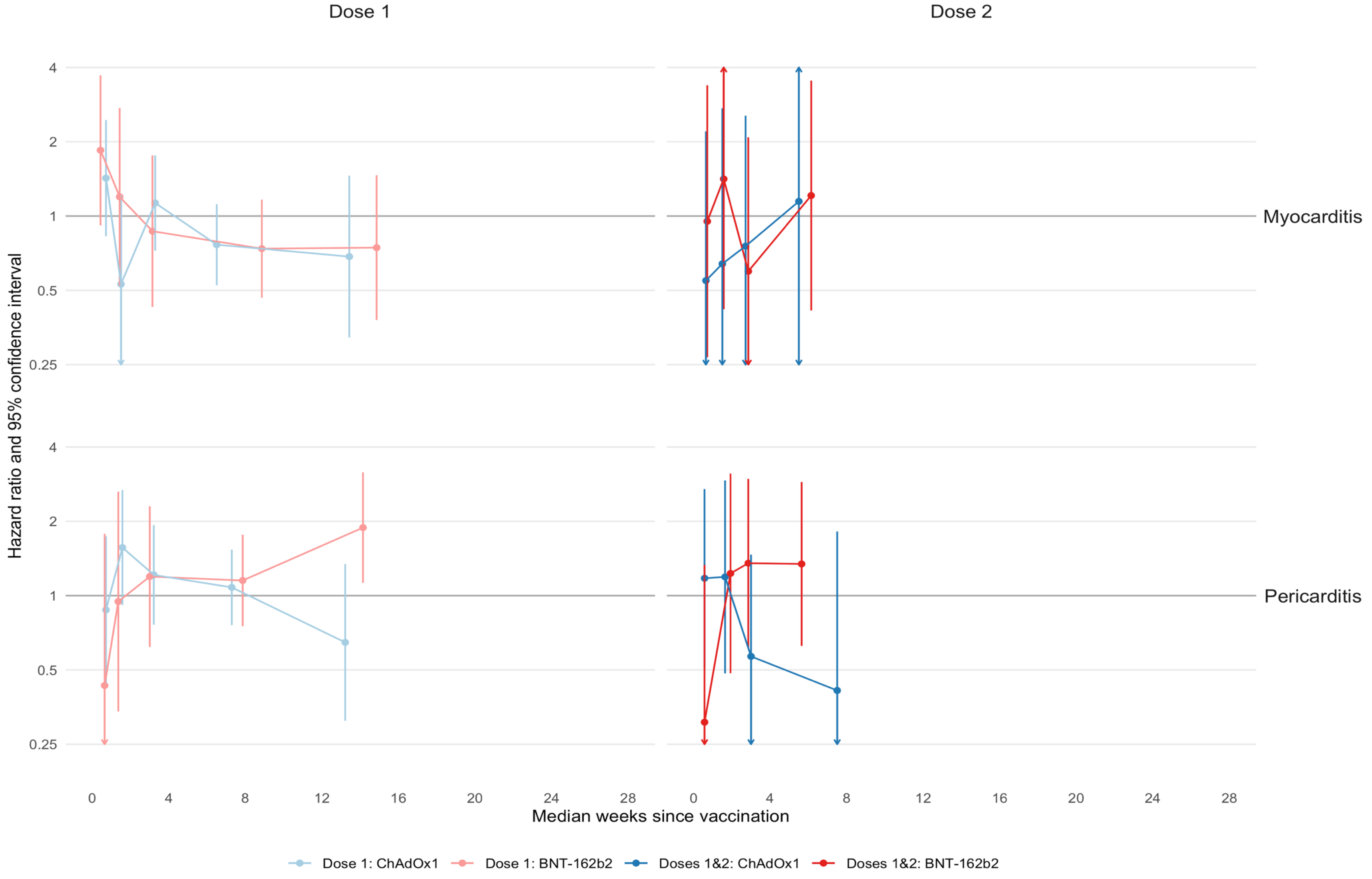

The number of people eligible for first and second dose analyses were 45,673,965; 28,549,265 respectively, until 17 May 2021.

The number of people who received a first dose of ChAdOx1, BNT-162b2 were 18,959,470 and 9,391,815, respectively; a second dose of ChAdOx1, BNT-162b2 were 7,992,035 and 8,133,695, respectively.

The numerical values of hazard ratios and 95% CIs are displayed in Supplementary Tables 30.
