# Supplementary material for: Cohort study of cardiovascular safety of different COVID-19 vaccination doses among 46 million adults in England
Source: Nat Commun. 2024 Jul 31;15:6085. doi: 10.1038/s41467-024-49634-x (PMC11291640; doi:10.1038/s41467-024-49634-x)
Supplement: Supplementary file 3 — Reporting Summary [file 41467_2024_49634_MOESM3_ESM.pdf]

nature portfolio

Corresponding author(s): Dr Samantha Ip

Last updated by author(s): Jun 1, 2024

Reporting Summary

Nature Portfolio wishes to improve the reproducibility of the work that we publish. This form provides structure for consistency and transparency in reporting. For further information on Nature Portfolio policies, see our Editorial Policies and the Editorial Policy Checklist.

Statistics

For all statistical analyses, confirm that the following items are present in the figure legend, table legend, main text, or Methods section.

n/a	Confirmed	
☐	☒	The exact sample size (n) for each experimental group/condition, given as a discrete number and unit of measurement
☐	☒	A statement on whether measurements were taken from distinct samples or whether the same sample was measured repeatedly
☒	☐	The statistical test(s) used AND whether they are one- or two-sided<br>*Only common tests should be described solely by name; describe more complex techniques in the Methods section.*
☐	☒	A description of all covariates tested
☐	☒	A description of any assumptions or corrections, such as tests of normality and adjustment for multiple comparisons
☐	☒	A full description of the statistical parameters including central tendency (e.g. means) or other basic estimates (e.g. regression coefficient) AND variation (e.g. standard deviation) or associated estimates of uncertainty (e.g. confidence intervals)
☒	☐	For null hypothesis testing, the test statistic (e.g. F, t, r) with confidence intervals, effect sizes, degrees of freedom and P value noted<br>*Give P values as exact values whenever suitable.*
☒	☐	For Bayesian analysis, information on the choice of priors and Markov chain Monte Carlo settings
☐	☒	For hierarchical and complex designs, identification of the appropriate level for tests and full reporting of outcomes
☒	☐	Estimates of effect sizes (e.g. Cohen's d, Pearson's r), indicating how they were calculated

Our web collection on statistics for biologists contains articles on many of the points above.

Software and code

Policy information about availability of computer code

Data collection	https://github.com/BHFDSC/CCU002_06/; Analyses used SQL and Python (in Databricks, version 3.68), and RStudio (Professional) Version 1.3.1093.1 driven by R Version 4.0.3 (10th October 2020)
Data analysis	https://github.com/BHFDSC/CCU002_06/; Analyses used SQL and Python (in Databricks, version 3.68), and RStudio (Professional) Version 1.3.1093.1 driven by R Version 4.0.3 (10th October 2020)

For manuscripts utilizing custom algorithms or software that are central to the research but not yet described in published literature, software must be made available to editors and reviewers. We strongly encourage code deposition in a community repository (e.g. GitHub). See the Nature Portfolio guidelines for submitting code & software for further information.

Data

Policy information about availability of data

All manuscripts must include a data availability statement. This statement should provide the following information, where applicable:
- Accession codes, unique identifiers, or web links for publicly available datasets
- A description of any restrictions on data availability
- For clinical datasets or third party data, please ensure that the statement adheres to our policy

Data used in this study are available in NHS England's Secure Data Environment service for England for England, but as restrictions apply are not publicly available (https://digital.nhs.uk/services/secure-data-environment-service). The CVD-COVID-UK/COVID-IMPACT programme led by the BHF Data Science Centre (https://

bhfdatasciencecentre.org/) received approval to access data in the NHS England's SDE service for England from the Independent Group Advising on the Release of Data (IGARD) (https://digital.nhs.uk/about-nhs-digital/corporate-information-and-documents/independent-group-advising-on-the-release-of-data) via an application made in the Data Access Request Service (DARS) Online system (reference: DARS-NIC-381078-Y9C5K; https://digital.nhs.uk/services/data-access-request-service-dars/dars-products-and-services). The CVD-COVID-UK/COVID-IMPACT Approvals & Oversight Board (https://bhfdatasciencecentre.org/areas/cvd-covid-uk-covid-impact/) subsequently granted approval to this project to access the data within NHS England's SDE service for England. The de-identified data used in this study were made available to accredited researchers only. Those wishing to access the data should contact bhfdsc@hdruk.ac.uk.

Research involving human participants, their data, or biological material

Policy information about studies with human participants or human data. See also policy information about sex, gender (identity/presentation), and sexual orientation and race, ethnicity and racism.

Reporting on sex and gender

Sex is recorded in electronic health records.
Our analyses were adjusted for sex. We also present subgroup analyses by sex.

Reporting on race, ethnicity, or other socially relevant groupings

Ethnicity is recorded in electronic health records.
Our analyses were adjusted for ethnicity. We also present subgroup analyses by ethnicity.

Population characteristics

Characteristics (number [%]) of the population under study for the dose 1, dose 2 and booster cohorts are detailed in Tables 1, Supplementary Table 1 and 2, respectively.
The characteristics described include: age, sex, ethnic group, Index of Multiple Deprivation (2010 IMD deciles grouped into Deciles 1-4, 5-6, 7-10/missing), smoking status (never/ever, with missing classified as "never"), medical history (including acute myocardial infarction (AMI), diabetes, depression, obesity, cancer, chronic obstructive pulmonary disease (COPD), liver disease, chronic kidney disease, dementia, all stroke, all venous thromboembolic events and thrombophilia), major surgery in the last year, number of unique medical conditions in the last year, prior COVID-19 at index date, medications taken in the last 90 days (including antiplatelets, blood pressure lowering, lipid-lowering, oral anticoagulants, combined oral contraceptives (COCP) and hormone replacement therapy (HRT)) and clinical vulnerability (clinically extremely vulnerable/ clinically vulnerable/neither). The medical history covariates were defined as a diagnosis of the condition before the index date except for diabetes which was additionally defined as a record of diabetic medication in the GDPPR data in the 90 days before the index date. Clinical vulnerability was defined on 8th December 2020. People were flagged as "clinically extremely vulnerable" using the SNOMED code 1300561000000107, and "clinically vulnerable" by identifying component conditions as applied in Table 3 of the COVID-19 chapter of the Green Book. Except for sex, clinical vulnerability and ethnic group, all other covariates were updated at individual-specific index dates for the dose 1, dose 2 and booster analyses. History of confirmed COVID-19 diagnosis was ascertained using established algorithms that combine information from SGSS, HES-APC, SUS, ONS deaths registry.

Recruitment

Cohorts are selected from linked electronic health records.

Ethics oversight

The North East – Newcastle and North Tyneside 2 research ethics committee provided ethical approval for the CVD-COVID-UK/COVID-IMPACT research programme (REC No 20/NE/0161) to access, within secure trusted research environments, unconsented, whole-population, de-identified data from electronic health records collected as part of patients' routine healthcare.

Note that full information on the approval of the study protocol must also be provided in the manuscript.

Field-specific reporting

Please select the one below that is the best fit for your research. If you are not sure, read the appropriate sections before making your selection.

☒ Life sciences ☐ Behavioural & social sciences ☐ Ecological, evolutionary & environmental sciences

For a reference copy of the document with all sections, see nature.com/documents/nr-reporting-summary-flat.pdf

Life sciences study design

All studies must disclose on these points even when the disclosure is negative.

Sample size

No sample size calculation was done as the study included the entire adult population of England that met the data exclusion criteria, totaling 45.7 million adults.

Data exclusions

People were included in the study if they were alive on 8th December 2020; aged 18-110 years inclusive; had a record in the GDPPR; recorded as male or female; and living in England. People with missing Lower-layer Super Output Area (LSOA) data were assumed to live in England. People were excluded if they were vaccinated before 8th December 2020; (2) they were recorded as having a second dose and/or a booster or third dose, before or without records of first and second dose vaccinations respectively; (3) the interval between their first and second vaccination was less than 21 days; (4) they had mixed first and second vaccine brands where the second dose was given on or before 7th May 2021; (5) the interval between their second and booster vaccinations was less than 90 days; (6) they had conflicting vaccination records or a situation code attached to any vaccination indicating that the vaccination was not given. We applied general quality checks, including removing people from the analysis who had nonsensical dates of birth or death (for details see https://github.com/BHFDSC/CCU002_06).

All eligible people were considered for the first dose analysis. Those who received a first dose of ChAdOx1, BNT-162b2, or mRNA1 were

	included in the second dose analyses. People who received the same vaccine brand for their first and second doses were included in the booster vaccination analyses.
Replication	The study's data are available within NHS England's Secure Data Environment, and the analytical code and codelists are accessible via our GitHub repository at BHFDSC/CCU002_06, enabling replication of our findings.
Randomization	As an observational study of health records, there was no experimental randomization of participants into groups. The data analysis compared health outcomes before and after vaccination across the entire dataset. Potential confounders was addressed through statistical methods appropriate for observational data.
Blinding	In the context of an observational study of existing health records, blinding is not applicable as the study does not involve experimental intervention or group allocation by the investigators. The investigators did not influence group allocation or data collection, which was retrospectively analyzed from health records.

Reporting for specific materials, systems and methods

We require information from authors about some types of materials, experimental systems and methods used in many studies. Here, indicate whether each material, system or method listed is relevant to your study. If you are not sure if a list item applies to your research, read the appropriate section before selecting a response.

Materials & experimental systems

n/a	Involved in the study	
☒	☐	Antibodies
☒	☐	Eukaryotic cell lines
☒	☐	Palaeontology and archaeology
☒	☐	Animals and other organisms
☐	☒	Clinical data
☒	☐	Dual use research of concern
☒	☐	Plants

Methods

n/a	Involved in the study	
☒	☐	ChIP-seq
☒	☐	Flow cytometry
☒	☐	MRI-based neuroimaging

Clinical data

Policy information about clinical studies

All manuscripts should comply with the ICMJE guidelines for publication of clinical research and a completed CONSORT checklist must be included with all submissions.

Clinical trial registration	NA
Study protocol	https://github.com/BHFDSC/CCU002_06/tree/main/protocol
Data collection	We analysed pseudonymised data, made available for the BHF Data Science Centre's CVD-COVID-UK/COVID-IMPACT Consortium within the NHS England Secure Data Environment (7,27), which is a secure, privacy-protecting platform. This data consists of linked datasets including General Practice Extraction Service Extract for Pandemic Planning and Research (GDPPR), hospital admission data from Secondary Uses Service (SUS), Hospital Episode Statistics for admitted patient care (HES-APC), national laboratory COVID-19 testing data from the Public Health England Second Generation Surveillance System (SGSS), Office for National Statistics (ONS) Civil Registration of Deaths (ONS deaths registry), medicines dispensed in primary care data and COVID-19 vaccination data. The study spanned from 8th December 2020, the start of the UK's vaccine rollout, to 23rd January 2022, the latest available data at the start of our analysis.
Outcomes	Eleven cardiovascular outcomes were analysed: AMI, ischaemic stroke, lower limb deep venous thrombosis (DVT), pulmonary embolism (PE), intracranial venous thrombosis (ICVT), mesenteric thrombus, portal vein thrombosis (PVT), any thrombocytopenia, subarachnoid haemorrhage & haemorrhagic stroke (SAH & HS), myocarditis and pericarditis. In addition, two composite outcomes were analysed: composite arterial (AMI, ischaemic stroke and other arterial embolism) and composite venous (PE, DVT, ICVT and PVT). We selected the earliest date of outcome event on or after index date from GDPPR, SUS, HES-APC and ONS deaths registry. We considered only the first/primary position from HES-APC and SUS and used the underlying cause from the death data, to differentiate acute, new events, from prevalent conditions. Further, we had previously found that aHRs for outcomes recorded as primary or secondary reason for admission or death were consistent with those from analyses of outcomes in the primary position.

Plants

Seed stocks	*Report on the source of all seed stocks or other plant material used. If applicable, state the seed stock centre and catalogue number. If plant specimens were collected from the field, describe the collection location, date and sampling procedures.*
Novel plant genotypes	*Describe the methods by which all novel plant genotypes were produced. This includes those generated by transgenic approaches, gene editing, chemical/radiation-based mutagenesis and hybridization. For transgenic lines, describe the transformation method, the number of independent lines analyzed and the generation upon which experiments were performed. For gene-edited lines, describe the editor used, the endogenous sequence targeted for editing, the targeting guide RNA sequence (if applicable) and how the editor was applied.*
Authentication	*Describe any authentication procedures for each seed stock used or novel genotype generated. Describe any experiments used to assess the effect of a mutation and, where applicable, how potential secondary effects (e.g. second site T-DNA insertions, mosiacism, off-target gene editing) were examined.*
